# Sequential appetite suppression by oral and visceral feedback to the brainstem

**DOI:** 10.1038/s41586-023-06758-2

**Published:** 2023-11-22

**Authors:** Truong Ly, Jun Y. Oh, Nilla Sivakumar, Sarah Shehata, Naymalis La Santa Medina, Heidi Huang, Zhengya Liu, Wendy Fang, Chris Barnes, Naz Dundar, Brooke C. Jarvie, Anagh Ravi, Olivia K. Barnhill, Chelsea Li, Grace R. Lee, Jaewon Choi, Heeun Jang, Zachary A. Knight

**Affiliations:** 1 Department of Physiology, University of California, San Francisco, San Francisco, CA USA; 2 Kavli Institute for Fundamental Neuroscience, University of California, San Francisco, San Francisco, CA USA; 3 Neuroscience Graduate Program, University of California, San Francisco, San Francisco, CA USA; 4 Howard Hughes Medical Institute, University of California, San Francisco, San Francisco, CA USA

**Keywords:** Hypothalamus, Neurophysiology

## Abstract

The termination of a meal is controlled by dedicated neural circuits in the caudal brainstem. A key challenge is to understand how these circuits transform the sensory signals generated during feeding into dynamic control of behaviour. The caudal nucleus of the solitary tract (cNTS) is the first site in the brain where many meal-related signals are sensed and integrated^1–4^, but how the cNTS processes ingestive feedback during behaviour is unknown. Here we describe how prolactin-releasing hormone (PRLH) and GCG neurons, two principal cNTS cell types that promote non-aversive satiety, are regulated during ingestion. PRLH neurons showed sustained activation by visceral feedback when nutrients were infused into the stomach, but these sustained responses were substantially reduced during oral consumption. Instead, PRLH neurons shifted to a phasic activity pattern that was time-locked to ingestion and linked to the taste of food. Optogenetic manipulations revealed that PRLH neurons control the duration of seconds-timescale feeding bursts, revealing a mechanism by which orosensory signals feed back to restrain the pace of ingestion. By contrast, GCG neurons were activated by mechanical feedback from the gut, tracked the amount of food consumed and promoted satiety that lasted for tens of minutes. These findings reveal that sequential negative feedback signals from the mouth and gut engage distinct circuits in the caudal brainstem, which in turn control elements of feeding behaviour operating on short and long timescales.

## Abstract (web summary)

Genetically distinct neural circuits in the caudal brainstem receive feedback from the mouth and gut to regulate feeding behaviour on short and long timescales.

## Main

The cNTS is the direct target of vagal afferents that innervate the gastrointestinal (GI) tract and detect GI stretch and intestinal nutrients^1–9^. These negative feedback signals are thought to gradually intensify as a meal progresses, thereby activating cNTS circuits that promote the termination of feeding. Consistently, the cNTS contains neurons that are important for satiation^10–17^, and these cells can be activated by meal-related signals, as measured by *Fos* expression^5,8,17^ and recordings in anaesthetized animals^7,18,19^ or brain slices^20^.

Nevertheless, the role of the cNTS in feeding behaviour has not been tested by recording the activity of these circuits in an awake animal. Thus, it remains unknown how slow feedback from the stomach and intestines—which accumulates over tens of minutes during and after feeding—is utilized by the brain to steer moment-by-moment decisions about behaviour. Nor is it known whether the cNTS detects other types of ingestive cues that also regulate feeding behaviour. Addressing these questions requires defining how the sensory signals generated during a meal are encoded in the circuits that are the direct recipients of visceral feedback.

The cNTS contains a diversity of genetically distinct cell types^10–17,21^, and one attractive model is that these cell types are tuned to sense different visceral signals, which, in turn, control different aspects of feeding behaviour. Although recordings in anaesthetized animals found that cNTS cell types show little specificity in their responses to different GI stimuli^7^, these anaesthetized preparations lack most of the sensory and motor feedback that is generated during natural ingestion. This raises the possibility that cNTS neurons may exhibit greater functional specificity in awake animals, as observed in other sensory systems^22^. We therefore investigated the natural dynamics of satiety-promoting cNTS cell types during a meal.

**Extended Data Fig. 1:**
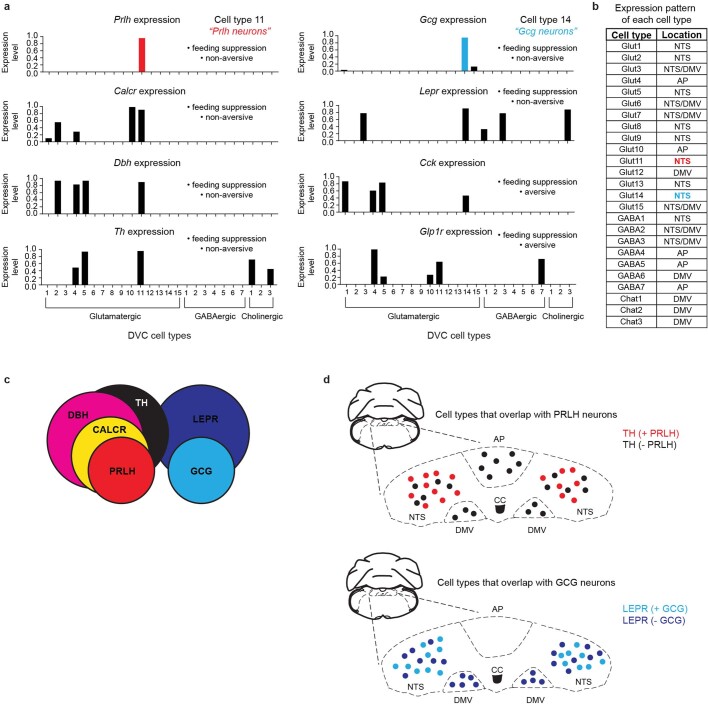
PRLH and GCG neurons are distinct cNTS cell types that regulate the non-aversive suppression of feeding. **a**, Unique cell types in the dorsal vagal complex (DVC) identified by single-cell RNA sequencing (Data are adapted from Ludwig et al. 2021^21^) that are known to regulate feeding. *Prlh* and *Gcg* expression label distinct cell types that are known to control the non-aversive suppression of feeding. **b**, Table describing where glutamatergic, GABAergic, and cholinergic cell types identified by single-cell sequencing are found in the DVC^21^. AP = area postrema. DMV = dorsal motor vagus. **c**, Schematic showing overlap in RNA expression between cell types that express *Dbh*, *Th*, *Calcr*, *Prlh*, *Lepr*, and *Gcg* based on data from panel a. These cell types have been shown to be activated by natural feeding and/or suppress food intake without inducing conditioned taste aversion (CTA). **d**, Schematic showing the location of cell types – which regulate non-aversive satiety – within the DVC based on data from panel b. Cells expressing *Prlh* (“PRLH neurons”) and *Gcg* (“GCG neurons”) are specifically located in the NTS, whereas other cell types show broad expression in the AP and DMV.

**Extended Data Fig. 2:**
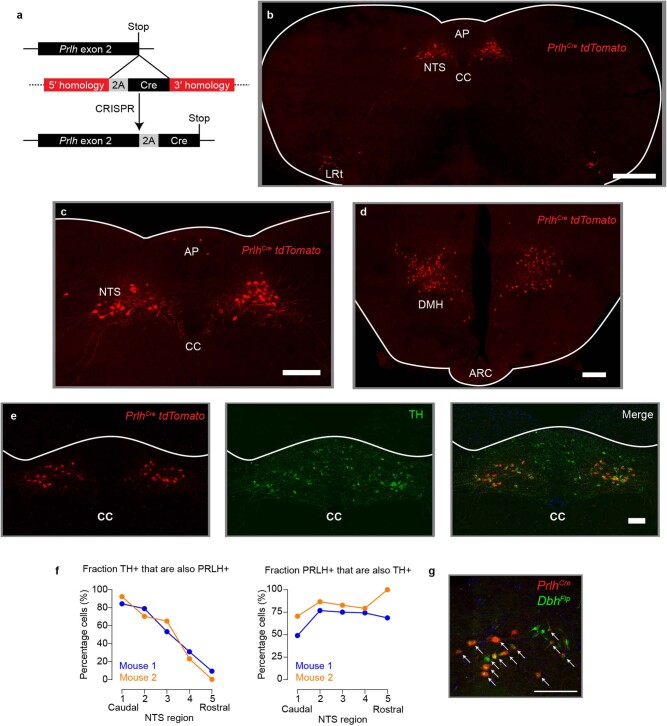
PRLH neurons largely overlap with TH neurons in the cNTS. **a**, Schematic for generating *Prlh*^*Cre*^ knock-in mice. **b**, Coronal slice at the level of the area postrema (AP) showing *Prlh*^*Cre*^ recombination. The AP, central canal (CC), NTS, and the lateral reticular formation (LRt) are labelled. The recombination pattern here and in the next panel (DMH) closely matches published reports of PRLH expression^15^. Scale bar = 500 µm. **c**, Zoom-in of AP/NTS. Scale bar = 200 µm. **d**, Recombination in the dorsomedial hypothalamus (DMH). The arcuate nucleus (ARC) is labelled for reference. Scale bar = 200 µm. **e**, *Prlh*^*Cre*^ recombination (red) and immunohistochemistry for tyrosine hydroxylase (TH) in the NTS at the level of the AP. This shows extensive co-localization, consistent with reports that PRLH and A2 neurons are overlapping in the cNTS. Scale bar = 100 µm. **f**, Co-localization between PRLH and TH across the rostrocaudal axis of the cNTS. Each region corresponds to 200 µm between −6.5 mm to −7.5 mm relative to bregma. **g**, *Prlh* and *Dbh* are co-localized in the cNTS. *Prlh*^*Cre*^ and *Dbh*^*Flp*^ mice were crossed to reporter mice to co-label neurons (Methods). Scale bar = 50 µm.

**Extended Data Fig. 3:**
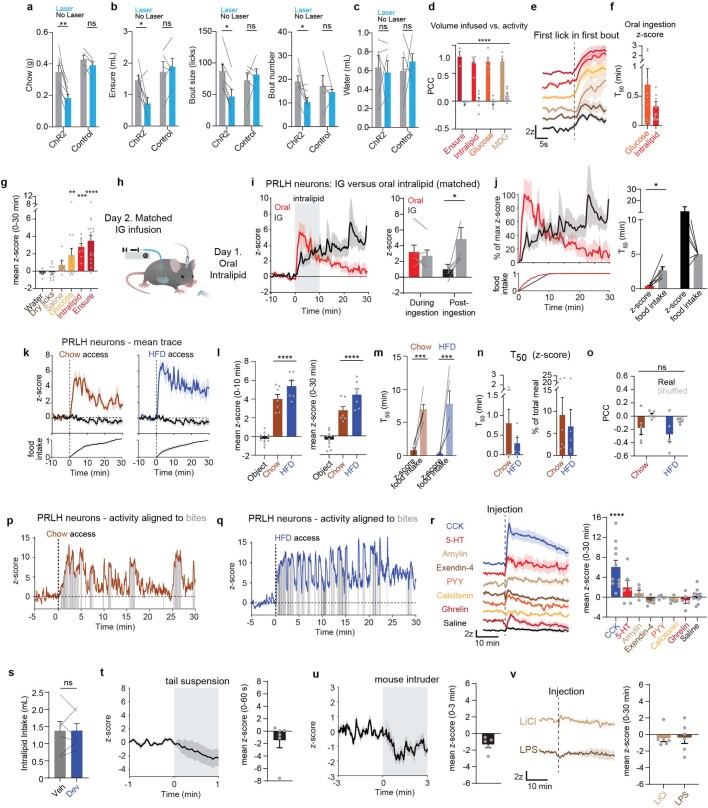
Regulation of PRLH neurons by ingestive and non-ingestive signals. **a**, Mean chow consumption (g) after open-loop stimulation (30 min; food-deprived mice) of PRLH neurons or no laser trials for ChR2-expressing mice or control mice. **b**, Left, mean Ensure consumption (mL) after open-loop stimulation (60 min; dark phase) of PRLH neurons or no laser trials. Middle, mean bout size (licks) after open-loop stimulation. Right, mean bout number after open-loop stimulation. **c**, Mean water consumption (mL) after open-loop stimulation (30 min; water-deprived) of PRLH neurons or no laser trials. **d**, Mean Pearson Correlation Coefficient (PCC) for the cumulative infusion volume vs. the z-scored change in activity during infusions. Real data (color) is compared vs. shuffled controls (gray). **e**, PSTH of PRLH neuron responses during just the first lick bout of the trial for the indicated solutions. **f**, Mean time to 50% of maximum z-score (T50) over the entire 30 min trial during oral ingestion of glucose or Intralipid. **g**, Mean z-scores (0–30 min) after lickometer access to tastants. **h**, Mice were fasted overnight before given access to Intralipid for 10 min of self-paced consumption (day 1). Two days later (day 2), fasted mice were given an IG infusion of Intralipid based on the amount consumed on day 1. **i**, Left, PSTH of PRLH neuron responses to volume-matched oral ingestion or IG infusion of Intralipid. Right, mean z-scores during oral ingestion or IG infusion (0–10 min) or post-ingestion (10–30 min). **j**, Left, PTSH of the percentage of max z-score during oral ingestion (red) or IG infusion (black) of Intralipid (volume-matched), with the percentage of total intake on the bottom panel. Right, mean time to reach 50% of the max z-score (”z-score”) versus mean time to consume 50% of total Intralipid (“food intake”) for oral ingestion (red) and IG infusion (black). **k**, Top, PSTH of PRLH neuron responses during self-paced chow or HFD consumption, or presentation of a non-food object (black), aligned to moment of food presentation. Bottom, cumulative fraction of total bites during the trial. **l**, Left, mean z-scores (0–10 min) after food/object access. Right, mean z-scores (0–30 min) after food/.object access. **m**, Mean time to reach 50% of the max z-score (“z-score”) versus mean time to consume 50% of total bites (“food intake”) for chow (brown) and HFD (blue). **n**, Left, mean time to 50% of maximum z-score (T50) over the entire 30 min trial during oral ingestion of chow or HFD. Right, mean percentage of total bites (“meal”) performed at the earliest time point with >50% of the max z-score (T50). **o**, Mean PCC for the relationship between the cumulative bites of chow or HFD and the z-scored change in activity across the entire 30 min trial. **p**, Example trace of calcium dynamics from PRLH neurons during chow consumption (individual bites are shown in gray). **q**, Example trace of calcium dynamics from PRLH neurons during HFD consumption (individual bites are shown in gray). **r**, Left, PSTH of PRLH neuron responses to IP injection of 5-HT, CCK, amylin, Exendin-4, PYY, calcitonin, ghrelin, and saline. Right, mean z-scores (0–30 min) after IP injection of each gut peptide. **s**, Devazepide pretreatment does not change total Intralipid consumption in PrlhCre mice (from Fig. 1l). **t**, Left, PSTH of PRLH neuron responses to tail suspension. Right, mean z-scores (0–60 s) during tail suspension. **u**, Left, PSTH of PRLH neuron responses to presentation of a same-sex mouse intruder. Right, mean z-scores (0–3 min) during intruder presentation. **v**, Left, PSTH of PRLH neuron responses to IP injection of LiCl (84 mg/kg) or LPS (100 ug/kg). Right, mean z-scores (0–30 min) after IP injection of LiCl or LPS. NS, *P < 0.05, **P < 0.01, ***P < 0.001, ****P < 0.0001. Data are mean ± sem. Statistics are shown in Supplementary Table 1.

We first investigated PRLH neurons, a cNTS cell type^21^ that is directly innervated by vagal afferents^14,20^, expresses *Fos* in response to ingestion^23^ and inhibits feeding without inducing conditioned taste avoidance^15^. For these reasons, these neurons are considered to be crucial for non-aversive satiety^3^ (Extended Data Fig. 1). We generated and validated *Prlh*^*cre*^ knock-in mice^17,23^ (616 ± 84 cNTS neurons per mouse (s.e.m.); Extended Data Fig. 2a–g) and showed that optogenetic stimulation of PRLH neurons in these mice inhibited food but not water intake (Extended Data Fig. 3a–c), confirming that these cells specifically regulate feeding.

**Fig. 1:**
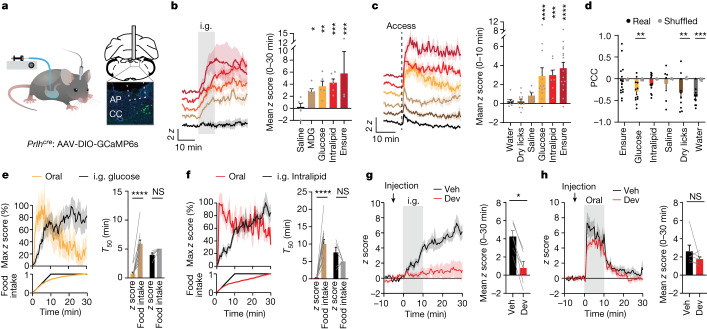
PRLH neurons show different responses to oral ingestion compared with i.g. infusion. **a**, Left, schematic of fibre photometry during i.g. infusions. Right, image of fibre placement and GCaMP6s expression in PRLH neurons. AP, area postrema; CC, central canal. **b**, Left, peri-stimulus time histogram (PSTH) of PRLH neuron responses for i.g. infusions (0–30 min, 1.5 ml) of indicated solutions (colours per the graph on the right). Right, *z* scores (0–30 min). Statistical comparisons are relative to the baseline prior to infusion. **c**, Left, PRLH neuron responses aligned to lickometer access for self-paced consumption (colours per the graph on the right). Right, *z* scores (0–10 min). **d**, PCC for the cumulative licks performed for each tastant compared with the *z*-scored change in activity across the 30-min trial. Real data (R; colour) are compared with shuffled controls (S; grey). **e**, Left, the percentage of maximum *z* scores during oral ingestion (orange) or i.g. infusion (black) of glucose (1.5 ml). The percentage of total intake is shown on the bottom. Right, the time to reach 50% of the maximum *z*-score plotted adjacent to the time required to consume 50% of total glucose (food intake, brown) or receive 50% of the total i.g. infusion (food intake, grey). **f**, As in **e**, except that data are for oral (red) versus i.g. Intralipid (black). **g**, Left, PRLH neuron responses to Intralipid i.g. infusion (0–10 min, 1.5 ml) after an i.p. injection of devazepide (Dev) or vehicle (Veh). Right, *z* scores (0–30 min). **h**, Left, PRLH neuron responses for Intralipid oral consumption after an i.p. injection of devazepide or vehicle. Right, *z* scores (0–30 min). NS, not significant, *P < 0.05, **P < 0.01, ***P < 0.001, ****P < 0.0001. Data are the mean ± s.e.m. Statistics are shown in Supplementary Table 1.

We next prepared mice for fibre photometry recordings of PRLH neurons in awake animals (Fig. 1a). Because PRLH neurons can be activated by GI feedback^23^, we equipped mice with intragastric (i.g.) catheters and measured responses to direct infusion of nutrients into the stomach (Fig. 1a). Infusion of the liquid diet Ensure (1.5 ml) resulted in a ramping activation (latency of 3.5 ± 0.6 min) that correlated with the amount infused, peaked several minutes after infusion ended (16 ± 4 min) and then remained elevated for the duration of the session (Fig. 1b, Extended Data Fig. 3d and Supplementary Video 1). Similar ramping activation was observed in response to infusions of glucose, fat (Intralipid) or MDG (an agonist of the intestinal glucose sensor SGLT1 (ref. ^24^)) but not saline (Fig. 1b; time to peak, 21 ± 2 min for glucose and 22 ± 4 min for Intralipid). Thus, PRLH neurons are progressively activated over tens of minutes following i.g. infusions of nutrients in a manner consistent with intestinal feedback^24^.

## Oral intake renders GI cues dispensable

We next fasted mice overnight and measured responses to self-paced feeding (30 min; Fig. 1c). In contrast to i.g. infusion of the same substances, PRLH neurons were activated within seconds by oral consumption of nutritive solutions (3.5 ± 0.6 *z* score (*z*) during Ensure consumption, *P* < 0.0001; Fig. 1c, Extended Data Fig. 3e–g and Supplementary Video 2). A similar rapid activation was observed during consumption of chow or a high-fat diet (HFD) but not water or saline (Extended Data Fig. 3k–q). Notably, PRLH neuron activation during oral ingestion did not further increase as the trial progressed and, as a result, PRLH neuron activity during natural feeding did not track cumulative food intake (in contrast to the response to i.g. infusions; Fig. 1d–f and Extended Data Fig. 3o). This discrepancy between oral and i.g. responses persisted when we precisely matched the amount and duration of nutrient delivery to the mouth and stomach (Extended Data Fig. 3h–j).

To test the necessity of GI signals during oral ingestion, we designed an experiment in which GI feedback could be blocked while mice consumed food by mouth. We did this by taking advantage of the fact that many gut–brain signals of fat ingestion are mediated by cholecystokinin (CCK)^19,24–26^. First, we confirmed that intraperitoneal (i.p.) injection of CCK activated PRLH neurons, consistent with *Fos* studies^27,28^ (Extended Data Fig. 3r). Next, we showed that PRLH neuron activation following i.g. infusion of Intralipid was abolished by prior injection of the CCKAR antagonist devazepide^19,25,26,29^, indicating that CCK is required for the response to i.g. infusion of fat (Fig. 1g). Finally, we repeated this experiment with mice consuming Intralipid by mouth (Fig. 1h). The activation of PRLH neurons by oral Intralipid was unaffected by devazepide pretreatment, even though this abolished the response to i.g. Intralipid in the same animals (Fig. 1g,h and Extended Data Fig. 3s). This reveals that, although all food consumed by mouth reaches the stomach, some feedback mechanisms that activate PRLH neurons following i.g. infusion become dispensable during a normal meal.

## PRLH neurons track ingestion dynamics

**Fig. 2:**
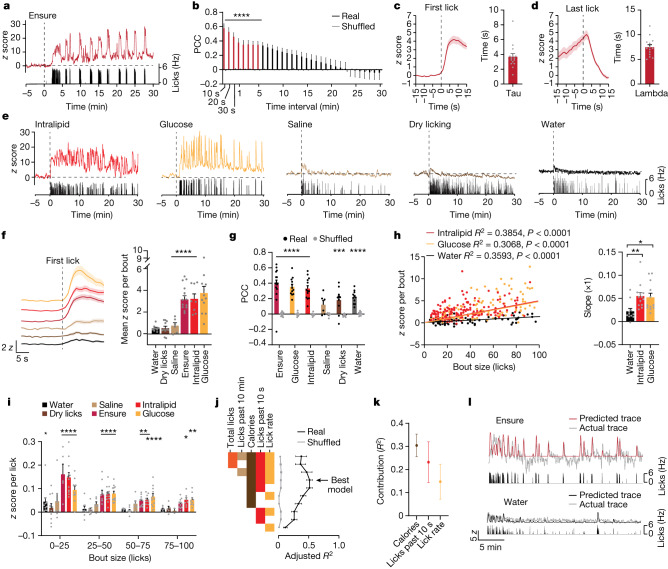
PRLH neurons track the dynamics of ingestion. **a**, Example traces of calcium dynamics of PRLH neurons during self-paced Ensure consumption. The lick rate is shown below. **b**, PCC for the relationship between the cumulative licks performed in the preceding time interval (indicated by red bars) and the *z*-scored change in activity during Ensure consumption. **c**, Left, PRLH neuron responses aligned to the first lick of the bout for Ensure consumption. Right, time constant (tau) when 63.8% of the *z*-scored activity change is reached. **d**, Left, PRLH neuron responses aligned to the last lick of the bout for Ensure consumption. Right, time constant (lambda) when the *z* score has decayed to 37% of its value during the last lick of the bout. **e**, Example traces of calcium dynamics during consumption of the indicated solutions or dry licking at an empty sipper. Dashed line indicates sipper access. **f**, PRLH neuron activity aligned to the first lick for all tastants (colours per the graph on the right). Right, response (0–10 s) after the first lick. **g**, PCC for the relationship between the instantaneous lick rate during consumption and the z-scored change in activity. Statistical comparisons are between real and shuffled data. **h**, Left, scatterplot for bout size versus the z score for all bouts during Intralipid, glucose or water consumption. Right, slope for Intralipid, glucose and water consumption. **i**, The *z* score per lick stratified by bout size for each animal. **j**, A GLM was constructed for each animal using subsets of variables (*n* = 10 mice). See Methods for details. Adjusted *R*^2^ (black line) is plotted against the shuffled controls (grey line). **k**, Contribution of individual variables to the variance explained (*R*^2^) by the best model. **l**, Examples of a predicted *z*-score trace using the GLM versus the actual *z*-score trace during Ensure or water consumption. *P < 0.05, **P < 0.01, ***P < 0.001, ****P < 0.0001. Data are the mean ± s.e.m. Statistics are shown in Supplementary Table 1.

**Extended Data Fig. 4:**
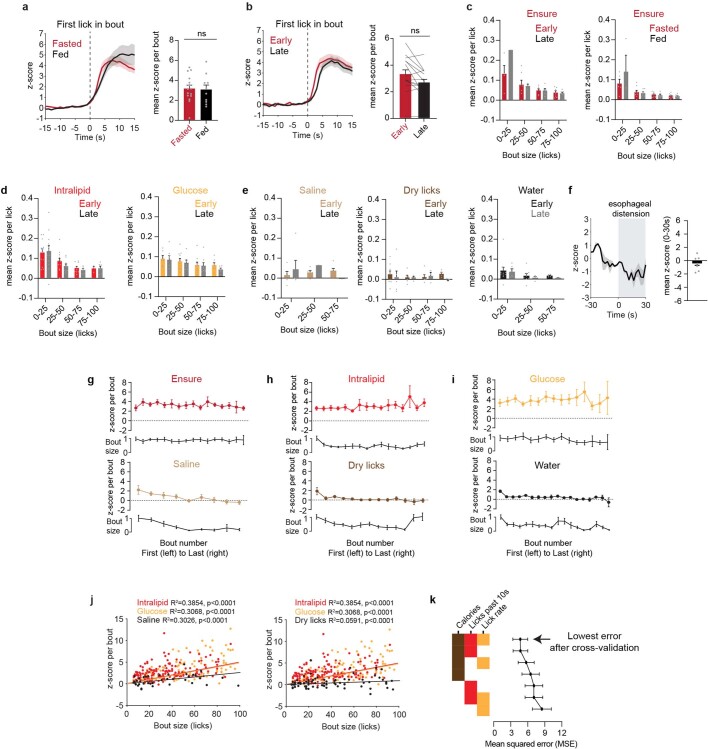
PRLH neurons track the moment-to-moment dynamics of ingestion. **a**, Left, PSTH of PRLH neuron activity aligned to the first lick of all lick bouts from overnight fasted mice (“fasted”) or ad libitum fed mice (“fed”) during a 30 min Ensure consumption test. Right, mean z-scores (0–10 s after first lick in bout) from fasted and fed mice. **b**, Left, PSTH of PRLH neuron activity aligned to the first lick of all lick bouts in the first 15 min of the trial (“early”) or the last 15 min of the trial (“late”) during a 30 min Ensure consumption test. Right, mean z-scores (0–10 s after first lick in bout) from early and late time intervals. **c**, Left, mean z-score per lick from Ensure consumption during early (first 15 min) vs. late (last 15 min) periods of a 30 min trial. Right, mean z-score per lick during Ensure consumption from overnight fasted (“fasted”) or ad libitum fed animals. **d**, Left, mean z-score per lick from Intralipid consumption during early (first 15 min) vs. late (last 15 min) periods of a 30 min trial. Right, mean z-score per lick from glucose consumption during early (first 15 min) vs. late (last 15 min) periods of a 30 min trial. **e**, Left, mean z-score per lick from saline consumption during early (first 15 min) vs. late (last 15 min) periods of a 30 min trial. Middle, mean z-score per lick from dry licking during early (first 15 min) vs. late (last 15 min) periods of a 30 min trial. Right, mean z-score per lick from water consumption during early (first 15 min) vs. late (last 15 min) periods of a 30 min trial. **f**, Left, PSTH of PRLH neuron responses during feeding tube insertion into the esophagus (0–30 s). Right, mean z-scores (0–30 s) during feeding tube insertion. **g**, Top, mean z-score for each bout of Ensure consumption (0–10 s) from the first bout (left) to the last bout (right), with the percentage of the max bout size (licks) shown below. Bottom, mean z-score for each bout of saline consumption (0–10 s) from the first bout to the last bout. **h**, Top, mean z-score for each bout of Intralipid consumption (0–10 s) from the first bout to the last bout. Bottom, mean z-score for each bout of dry licking (0–10 s) from the first bout to the last bout. **i**, Top, mean z-score for each bout of glucose consumption (0–10 s) from the first bout to the last bout. Bottom, mean z-score for each bout of water consumption (0–10 s) from the first bout to the last bout. **j**, Left, scatterplot showing the relationship between bout size (# of licks in the first 10 s of each bout) and mean z-score (0–10 s of each bout) for all bouts during Intralipid, glucose, or saline consumption. Each dot represents a single lick bout. Right, scatterplot showing the relationship between bout size and mean z-score for all bouts during Intralipid or glucose consumption, or dry licking at an empty bottle. **k**, The mean squared error (MSE) is plotted for each model after performing cross-validation. To perform cross-validation, 80% of the data was used to train the GLM coefficients and calculate the MSE from the remaining 20% of the data. This was performed for 100 iterations to obtain an average MSE value for each animal. NS, *P < 0.05, **P < 0.01, ***P < 0.001, ****P < 0.0001. Data are mean ± sem. Statistics are shown in Supplementary Table 1.

PRLH neuron activity was most strongly correlated with intake in the preceding 10 s, which indicates that these cells are regulated by oral contact with food or its close correlate (Pearson’s correlation coefficient (PCC) = 0.60 ± 0.01, *P* < 0.0001 compared with shuffled controls; Fig. 2a,b). Consistently, the activation of these neurons during ingestion of liquid diets was time-locked to licking (Fig. 2a and Supplementary Video 3). For example, during Ensure consumption, PRLH neuron activity rapidly increased after the first lick in each bout (*τ* = 3.7 ± 0.4 s; Fig. 2c) and then declined to baseline after licking stopped (*λ* = 7.5 ± 0.5 s; Fig. 2d) in a manner that did not vary with satiety state or trial progression (Extended Data Fig. 4a–c). Glucose and Intralipid consumption induced similar time-locked activation of PRLH neurons (Fig. 2e–g and Extended Data Fig. 4d), indicating that this rapid response is not linked to a single macronutrient. By contrast, these responses were substantially smaller when mice drank water or saline (indicating that fluid consumption is insufficient), when they performed dry licks at an empty sipper (indicating that motor signals are insufficient) or following insertion of an oral gavage needle into the oesophagus (indicating that oesophageal distension has a limited role) (Fig. 2e,f and Extended Data Fig. 4e–i). We also observed limited responses to social or stressful stimuli or sickness-inducing agents (Extended Data Fig. 3t–v). Thus, PRLH neurons are specifically and rapidly activated by oral contact with food.

The stronger activation of PRLH neurons by food relative to water could be a direct consequence of its sensory properties (for example, taste or nutrient content) or secondary to differences in behaviour (for example, faster ingestion of nutritive solutions). There was a small increase in the neural response per bout for larger bouts of water consumption (Fig. 2h), indicating that the ingestion rate influences PRLH neuron activity independent of nutrients. However, consumption of glucose and Intralipid resulted in larger PRLH neuron responses at all bout sizes (Fig. 2h,i and Extended Data Fig. 4j). To further characterize the contribution of these variables, we built a generalized linear model (GLM) of PRLH neuron dynamics during consumption of multiple tastants (Fig. 2j–l). The model that explained the most variance incorporated a constant variable that indicated whether the tastant had calories, cumulative intake in the preceding 10 s and the instantaneous lick rate (Fig. 2j,k and Extended Data Fig. 4k). Thus PRLH neurons are activated by a signal linked to the chemical properties of food, which then interacts with the ingestion rate.

## PRLH neurons are activated by food tastes

**Extended Data Fig. 5:**
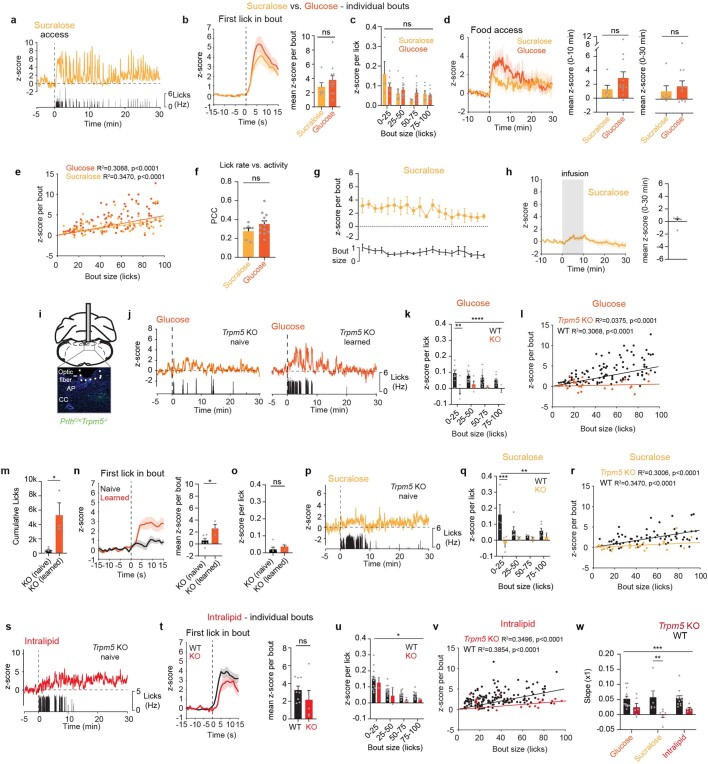
PRLH neurons are activated by food tastes. **a**, Example trace of calcium dynamics from PRLH neurons during sucralose consumption. **b**, Left, PSTH of PRLH neuron activity aligned to the first lick of all lick bouts during sucralose or glucose consumption. Right, mean response (0–10 s after first lick) averaged across all lick bouts for sucralose and glucose consumption. **c**, Mean z-score per lick stratified by bout size for sucralose and glucose consumption. **d**, Left, PSTH of PRLH neuron responses to sucralose or glucose consumption. Middle, mean z-scores (0–10 min) during sucralose or glucose consumption. Right, mean z-scores (0–30 min) during sucralose or glucose consumption. **e**, Scatterplot showing the relationship between bout size (# of licks in the first 10 s of each bout) and mean z-score (0–10 s of each bout) for all bouts during sucralose or glucose consumption. **f**, Mean PCC for the relationship between the instantaneous lick rate each second during consumption of sucralose or glucose and the z-scored change in activity across the entire 30 min trial. **g**, Mean z-score for each bout of sucralose consumption (0–10 s) from the first bout (left) to the last bout (right), with the percentage of the max bout size (licks) shown below. **h**, Left, PSTH of PRLH neuron responses to IG infusion of sucralose (1.5 mL). Right, mean z-scores (0–30 min) during IG infusion of sucralose. **i**, Fiber placement and GCaMP6s expression in PRLH neurons of a *Trpm5*^*−/−*^ mouse. **j**, Left, example trace of calcium dynamics from PRLH neurons during glucose consumption from a naïve *Trpm5*^*−/−*^ mouse. Right, Left, example trace from a learned *Trpm5*^*−/−*^ mouse. **k**, Mean z-score per lick stratified by bout size for glucose consumption in WT and *Trpm5*^*−/−*^ mice. **l**, Scatterplot showing the relationship between bout size (# of licks in the first 10 s of each bout) and mean z-score (0–10 s of each bout) for all bouts during glucose consumption from WT and *Trpm5*^*−/−*^ mice. **m**, Mean cumulative licks performed during a glucose consumption test by naive and learned *Trpm5*^*−/−*^ mice. Animals were defined as “learned” if they performed at least 1000 licks during the second test (Methods). **n**, PSTH of PRLH neuron responses across all glucose lick bouts in naive and learned *Trpm5*^*−/−*^ mice. **o**, Mean z-score per lick for glucose consumption in naive and learned *Trpm5*^*−/−*^ mice. **p**, Example trace of calcium dynamics from PRLH neurons during sucralose consumption from a *Trpm5*^*−/−*^ mouse. **Q**, Mean z-score per lick stratified by bout size for sucralose consumption in WT and *Trpm5*^*−/−*^ mice. **R**, Scatterplot showing the relationship between bout size and mean z-score for all bouts during sucralose consumption from WT and *Trpm5*^*−/−*^ mice. **S**, Example trace of calcium dynamics from PRLH neurons during Intralipid consumption from a *Trpm5*^*−/−*^ mouse. **T**, Left, PSTH of PRLH neuron responses across all Intralipid lick bouts in WT and *Trpm5*^*−/−*^ mice. Right, mean z-scores (0–10 s) for all lick bouts during Intralipid consumption. **U**, Mean z-score per lick stratified by bout size for Intralipid consumption in WT and *Trpm5*^*−/−*^ mice. **V**, Left, scatterplot showing the relationship between bout size and mean z-score for all bouts during Intralipid consumption from WT and *Trpm5*^*−/−*^ mice. **W**, mean slope (coefficient x1) for glucose, sucralose, and Intralipid consumption from WT and *Trpm5*^*−/−*^ mice. NS, *P < 0.05, **P < 0.01, ***P < 0.001, ****P < 0.0001. Data are mean ± sem. Statistics are shown in Supplementary Table 1.

The preferential activation of PRLH neurons by caloric foods could be due to their nutrient content or their taste. Consumption of the non-caloric sweetener sucralose caused strong, time-locked activation during licking that was similar to glucose in dynamics and magnitude, whereas i.g. infusions of sucralose did not activate PRLH neurons (Extended Data Fig. 5a–h). Thus, sweet taste alone is sufficient to activate PRLH neurons during normal ingestion.

**Fig. 3:**
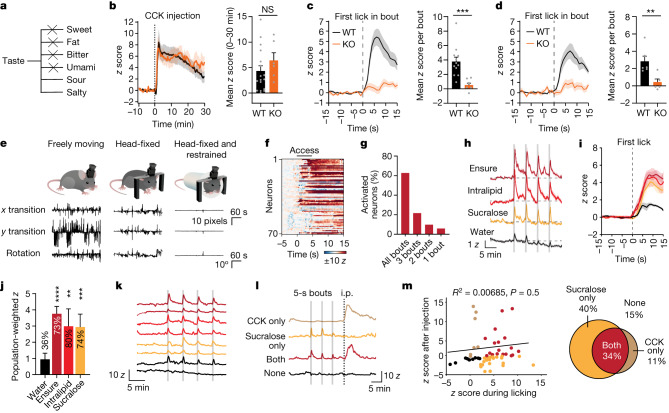
PRLH neurons are activated by the taste of food. **a**, Taste impairments in taste-blind *Trpm5*^*−/−*^ mice. **b**, Left, PRLH neuron responses aligned to i.p. injection of CCK in *Trpm5*^*−/−*^ mice and WT controls. Right, *z* scores (0–30 min). **c**, Left, PRLH neuron responses across all glucose lick bouts in WT and *Trpm5*^*−/−*^ mice. Right, z scores (0–10 s). **d**, Left, PRLH neuron responses across all sucralose lick bouts in WT and *Trpm5*^*−/−*^ mice. Right, *z* scores (0–10 s). **e**, Top, schematic of microendoscopy imaging of PRLH neurons in a freely moving, head-fixed or head-fixed and restrained mouse. Bottom, example trace of movement artefacts detected using Mosaic analysis software in each configuration during imaging. **f**, Heatmap of individual neuron responses to the first bout of brief access (5 s) of Ensure consumption. **g**, Percentage of neurons activated by all four bouts, only three bouts, only two bouts or only-lick bout during Ensure consumption. **h**, PRLH neuron responses aligned to brief access consumption of Intralipid, sucralose and water (averaged across all neurons). **i**, PRLH neuron responses aligned to the first lick of all lick bouts (averaged across all lick bouts and neurons). **j**, Population-weighted *z* score (calculated as the fraction of neurons activated multiplied by their *z*-scored activity change) for consumption of the indicated solutions. The percentage of neurons activated are listed above each bar. Statistical comparisons are relative to consumption of water. **k**, Example traces of calcium dynamics in representative neurons during consumption of the indicated solutions (colours per **j**). **l**, Example traces of calcium dynamics in representative neurons responding to sucralose consumption, a CCK injection, both stimuli or neither. **m**, Left, scatterplot of *z* scores during brief access sucralose consumption (averaged across all lick bouts) versus *z* scores after CCK injection. Right, Venn diagram showing the percentage of cells activated by these stimuli. *P < 0.05, **P < 0.01, ***P < 0.001, ****P < 0.0001. Data are the mean ± s.e.m. Statistics are shown in Supplementary Table 1.

To test whether taste is required for the lick-triggered activation of PRLH neurons, we crossed *Prlh*^*cre*^ mice into the background of ‘taste-blind’ *Trpm5* knockout mice (*Prlh*^*cre*^*Trpm5*^−/−^), so that we could perform photometry recordings of PRLH neurons in mice that have a substantially reduced ability to taste (Fig. 3a and Extended Data Fig. 5i). CCK injection activated PRLH neurons to a similar extent in both taste-blind mice and wild-type (WT) controls (Fig. 3b), but responses to glucose ingestion were greatly reduced in taste-blind animals (3.8 ± 0.6 *z* in WT mice compared with 0.6 ± 0.3 *z* in taste-blind mice, *P* = 0.0004; Fig. 3c and Extended Data Fig. 5j–l,w). These differences persisted after accounting for the number of licks in each bout (Extended Data Fig. 5k) and were not fully rescued by long-term exposure to glucose (Extended Data Fig. 5m–o). We observed a similar loss of neural responses to ingestion of sucralose, but not Intralipid, in taste-blind mice (Fig. 3d and Extended Data Fig. 5p–w). Thus the activation of PRLH neurons by sweet substances requires taste signalling, whereas fat may be detected by TRPM5-independent pathways^30^.

**Extended Data Fig. 6:**
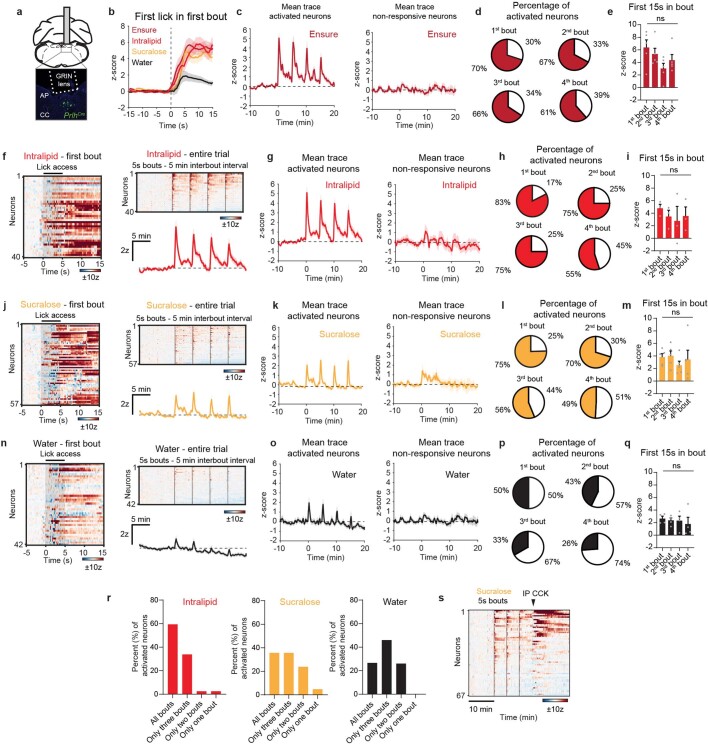
Individual PRLH neurons are activated by food tastes. **a**, GRIN lens placement and GcaMP6s expression in PRLH neurons. **b**, PSTH of PRLH neuron responses during the first lick bout of the trial for the indicated solutions. **c**, Left, PTSH of activated PRLH neurons (mean z-score > 1 across all 5 s bouts) during Ensure consumption from Fig. 3. Right, PTSH of non-responsive PRLH neurons (mean z-score <1 across all 5 s bouts). **d**, Percentage of activated and non-responsive neurons during each Ensure lick bout. **e**, Mean z-score for each 5 s bout during Ensure consumption. Each data point is the averaged value from a single animal. **f**, Left, heatmap of individual neuron responses to the first bout of Intralipid consumption. Right, heatmap of individual neuron responses to brief access (5 s) Intralipid consumption at 5 min intervals over 20 min. Bottom, PTSH aligned to brief access Intralipid consumption (averaged across all neurons). **g**, Left, PTSH of activated PRLH neurons (mean z-score > 1 across all 5 s bouts) during Intralipid consumption from Fig. 3. Right, PTSH of non-responsive PRLH neurons (mean z-score <1 across all 5 s bouts). **h**, Percentage of activated and non-responsive neurons during each Intralipid lick bout. **i**, Mean z-score for each 5 s bout during Intralipid consumption. **j**, Left, heatmap of individual neuron responses to the first bout of sucralose consumption. Right, heatmap of individual neuron responses to brief access (5 s) sucralose consumption. Bottom, PTSH aligned to brief access sucralose consumption (averaged across all neurons). **k**, Left, PTSH of activated PRLH neurons (mean z-score > 1 across all 5 s bouts) during sucralose consumption from Fig. 3. Right, PTSH of non-responsive PRLH neurons (mean z-score <1 across all 5 s bouts). **l**, Percentage of activated and non-responsive neurons during each sucralose lick bout. **m**, Mean z-score for each 5 s bout during sucralose consumption. **n**, Left, heatmap of individual neuron responses to the first bout of water consumption. Right, heatmap of individual neuron responses to brief access (5 s) water consumption. Bottom, PTSH aligned to brief access water consumption (averaged across all neurons). **o**, Left, PTSH of activated PRLH neurons (mean z-score > 1 across all 5 s bouts) during water consumption from Fig. 3. Right, PTSH of non-responsive PRLH neurons (mean z-score <1 across all 5 s bouts). **p**, Percentage of activated and non-responsive neurons during each water lick bout. **q**, Mean z-score for each 5 s bout during water consumption. **r**, Left, percentage of neurons activated by all four bouts, three bouts, two bouts, or only one lick bout for Ensure consumption. Right, percentage of neurons activated by all four bouts, three bouts, two bouts, or only one lick bout for Intralipid consumption. **s**, Heatmap of individual neuron responses to brief access sucralose consumption before an IP injection of CCK. NS, *P < 0.05, **P < 0.01, ***P < 0.001, ****P < 0.0001. Data are mean ± sem. Statistics are shown in Supplementary Table 1.

Fibre photometry records population responses but cannot reveal the activity of individual neurons. Previous efforts to perform single-cell imaging in the cNTS of awake animals have been hindered by large motion artefacts^7^. However, we found that combining head-fixation with lower body restraint was sufficient to enable stable single-cell recordings of PRLH neurons while mice consumed liquid diets (Fig. 3e, Extended Data Fig. 6a and Supplementary Videos 4–6). Mice were deprived of food overnight, acclimated to the restraint and then given brief access to different tastants. Ensure consumption rapidly activated most PRLH neurons (70% of cells) in a manner that was triggered by contact with food, reached a peak near the end of the bout (*τ* = 5.8 ± 0.4 s) and then gradually decayed when the sipper was removed (Fig. 3f,g, Extended Data Fig. 6b–e and Supplementary Video 7). Intralipid or sucralose consumption produced similar responses, whereas fewer cells were activated by water consumption and the magnitude of their activation was smaller (Fig. 3h–k and Extended Data Fig. 6f–r). Thus individual PRLH neurons are activated by tastes associated with food but only weakly by ingestion per se.

The high percentage of cells activated by consumption of sweet solutions (74% of all neurons with sucralose) and fatty solutions (80% of all neurons with Intralipid) implies that most PRLH neurons are not specialized to respond to a single taste. To examine whether gustatory and post-ingestive signals activate the same neurons, mice were allowed to briefly lick sucralose before receiving an injection of CCK. We observed strong, sustained activation of many PRLH neurons in response to CCK (Fig. 3l and Extended Data Fig. 6s), but the overlap between these CCK-activated cells and those that responded to sucralose was not different from what would be expected by chance (*P* = 0.784, Fisher’s exact test), and the magnitude of the individual cell responses to these two stimuli was not correlated (Fig. 3m). Thus gustatory and visceral signals each activate a large and partially overlapping subset of PRLH neurons.

## PRLH neurons pace food ingestion

**Fig. 4:**
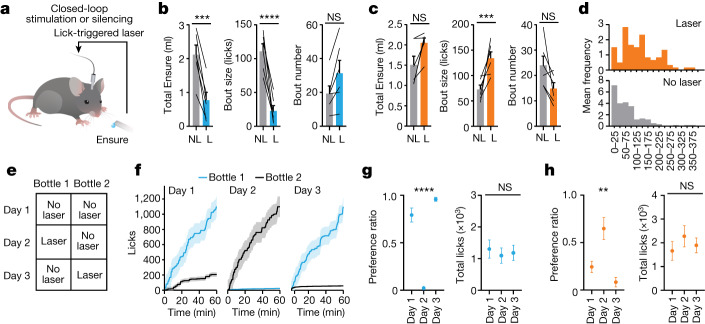
PRLH neurons pace food ingestion. **a**, Schematic of closed-loop optogenetic stimulation or silencing of PRLH neurons expressing ChR2 or GtACR1. **b**, Left, Ensure consumption during closed-loop optogenetic stimulation (60-min trial) of PRLH neurons expressing ChR2 in either laser (L) or no laser (NL) trials. Middle, bout size (licks). Right, bout number. **c**, Left, Ensure consumption during closed-loop silencing (60 min) of PRLH neurons expressing GtACR1 in either laser or no laser trials. Middle, bout size. Right, bout number. **d**, Distribution of bout sizes (bins are 25 lick increments) for trials in which PRLH neurons received closed-loop silencing (top) or no laser trials (bottom). **e**, Schematic for two-bottle preference test in which animals are presented with two identical bottles containing Ensure. Day 1 establishes initial preference. On day 2, the preferred or less preferred bottle is paired with optogenetic stimulation or silencing, respectively. The pairing is switched on day 3. **f**, Cumulative licks of Ensure for the preferred (bottle 1) versus the non-preferred (bottle 2) bottle on days 1–3. **g**, Preference ratio for bottle 1 (licks from bottle 1/total licks) and total Ensure consumption (licks from bottle 1 + licks from bottle 2) from days 1 to 3 of the closed-loop stimulation paradigm. Statistical comparisons are relative to day 1. **h**, Preference ratio for bottle 1 and total Ensure consumption from days 1 to 3 of the closed-loop silencing paradigm. *P < 0.05, **P < 0.01, ***P < 0.001, ****P < 0.0001. Data are the mean ± s.e.m. Statistics are shown in Supplementary Table 2. Genotype controls (no opsin or Cre ± laser) for all experiments are shown in Extended Data Fig. 7.

**Extended Data Fig. 7:**
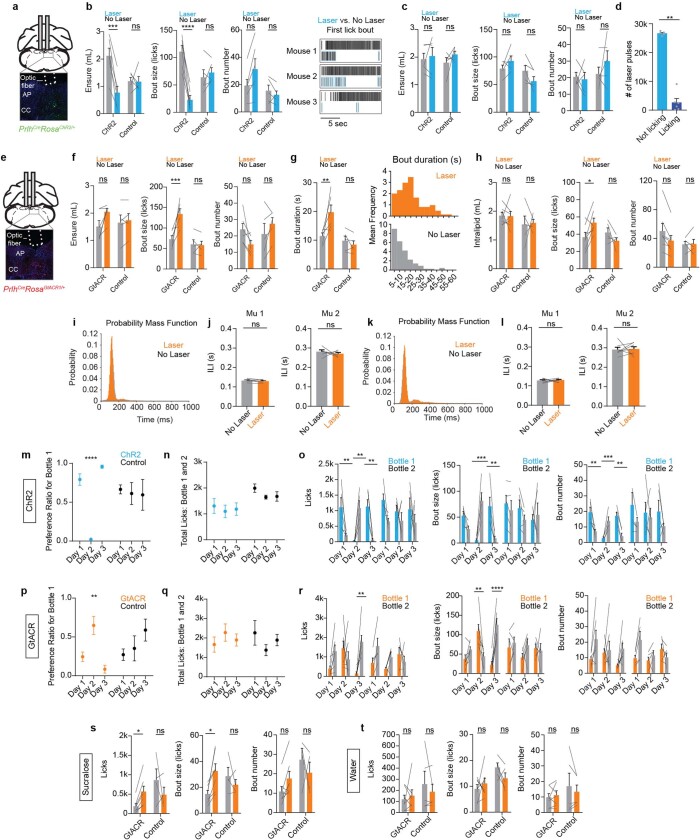
PRLH neurons pace food ingestion by modulating valence associated with food tastes. This figure extends the data from Fig. 4 by showing, for each experiment, the response of genetic controls (”controls” – mice that lack opsin or Cre expression) to the given laser stimulation protocol. The data from opsin-expressing mice (”ChR2” or “GtACR”) are also reproduced here to enable direct comparison with controls. **a**, Fiber placement and ChR2-GFP expression in PRLH neurons of the cNTS. **b**, Left, mean Ensure consumption (mL) after closed-loop stimulation (60 min; dark phase) of PRLH neurons (when animals are licking) or sham trials. Middle, mean bout size (licks) after closed-loop stimulation. Right, mean bout number after closed-loop stimulation. Example raster plots from three animals showing individual licks of Ensure. **c**, Left, mean Ensure consumption (mL) during closed-loop stimulation (60 min; dark phase feeding) of PRLH neurons (when animals are not licking) or no laser trials. Middle, mean bout size (licks) during closed-loop stimulation. Right, mean bout number during closed-loop stimulation. **d**, Mean number of laser pulses received by individual animals receiving closed-loop stimulation during licking or when not actively licking. **e**, Fiber placement and GtACR1-FusionRed expression in PRLH neurons of the cNTS. **f**, Left, mean Ensure consumption (mL) during closed-loop silencing (60 min; dark phase feeding) of PRLH neurons or no laser trials. Middle, mean bout size (licks) during closed-loop silencing. Right, mean bout number during closed-loop silencing for Ensure consumption. **g**, Left, mean bout duration (s) during closed-loop silencing. Left, distribution of bout durations (bins are 5 s) for trials in which PRLH neurons received closed-loop silencing (top) or no laser trials (bottom). **h**, Left, mean Intralipid consumption (mL) during closed-loop silencing (60 min; dark phase feeding) of PRLH neurons or no laser trials. Middle, mean bout size (licks) during closed-loop silencing. Right, mean bout number during closed-loop silencing. **i**, Probability mass function (PMF) for interlick interval (ILI) between 0-1 s during closed-loop silencing of PRLH neurons or no laser trials (Ensure consumption). **j**, Left, mean values for mu1 constant (left peak on PMF). Right, mean values for mu2 constant (right peak on PMF). **k**, Probability mass function (PMF) for interlick interval (ILI) between 0-1 s during closed-loop silencing of PRLH neurons or no laser trials (Intralipid consumption). **l**, Left, mean values for mu1 constant (left peak on PMF). Right, mean values for mu2 constant (right peak on PMF). **m**, Preference ratio for bottle 1 (licks from bottle 1/licks from bottle 1 + licks from bottle 2) from day 1–3 of closed-loop stimulation paradigm (when animals are licking). **n**, Total Ensure consumption (licks from bottle 1 + licks from bottle 2) from day 1–3 of closed-loop stimulation paradigm. **o**, Left, mean number of licks for bottle 1 and bottle 2 from day 1–3 of closed-loop stimulation paradigm (Ensure consumption). Middle, mean bout size (licks) for bottle 1 and 2 from day 1–3. Right, mean bout number for bottle 1 and 2 from day 1–3. **p**, Preference ratio for bottle 1 from day 1–3 of closed-loop silencing paradigm. **q**, Total Ensure consumption from day 1–3 of closed-loop silencing paradigm. **r**, Left, mean number of licks for bottle 1 and 2 from day 1–3 of closed-loop silencing paradigm (Ensure consumption). Middle, mean bout size (licks) for bottle 1 and 2 from day 1–3. Right, mean bout number for bottle 1 and 2 from day 1–3. **s**, Left, mean number of licks for bottle 1 and 2 from closed-loop silencing paradigm (sucralose consumption). Middle, mean bout size (licks) for bottle 1 and 2. Right, mean bout number for bottle 1 and 2. **t**, Left, mean number of licks for bottle 1 and 2 from closed-loop silencing paradigm (water consumption). Middle, mean bout size (licks) for bottle 1 and 2. Right, mean bout number for bottle 1 and 2. NS, *P < 0.05, **P < 0.01, ***P < 0.001, ****P < 0.0001. Data are mean ± sem. Statistics are shown in Supplementary Table 2.

Stimulation of PRLH neurons inhibits food intake (Extended Data Fig. 3a,b), but this tonic activation does not match the natural, ingestion-triggered activity of these cells. We therefore selectively manipulated PRLH neuron activity during licking using closed-loop optogenetics (Fig. 4a). Stimulation during licking (lick-on) decreased Ensure consumption through a reduction in bout size (111 ± 11 licks without laser stimulation compared with 23 ± 8 licks with laser stimulation, *P* < 0.0001) with no effect on bout number (Fig. 4b and Extended Data Fig. 7a,b). By contrast, stimulation of PRLH neurons only when mice were not licking (lick-off) had no effect on ingestion, even though mice received ten times more laser pulses compared with the lick-on test (Extended Data Fig. 7c,d). Thus, stimulation of PRLH neurons only inhibits food intake when mice are actively licking.

To test whether the natural bursts in PRLH neuron activity during licking are required for the regulation of feeding, we targeted the optogenetic silencer GtACR1 to PRLH neurons and performed closed-loop inhibition (Fig. 4c,d and Extended Data Fig. 7e–g). Silencing PRLH neurons during licking increased the bout size for consumption of both Ensure and Intralipid, as measured by the number of licks and duration of licking (Fig. 4c,d and Extended Data Fig. 7f–h). Thus, PRLH neurons influence feeding primarily by regulating the size of ingestion bursts.

We next investigated the mechanism by which PRLH neurons restrain the size of individual bouts. Several observations suggest that PRLH neurons do not directly control motor circuits (Extended Data Fig. 7i–l and Supplementary Discussion). On the other hand, the size or duration of a lick bout is correlated with the palatability of an ingested solution and can reflect hedonic motivation or ‘liking’^31^. This suggests that PRLH neurons may modulate feeding bursts by rapidly altering the valence of ingestion. To test this idea, we examined whether PRLH neuron stimulation or silencing could bias the real-time preference of an animal for one of two sippers containing identical solutions (Fig. 4e). Pairing the preferred sipper with PRLH neuron stimulation caused an almost complete switch in sipper preference, such that animals drank only from the bottle that lacked stimulation (preference ratio of 0.8 ± 0.1 on day 1 compared with 0.02 ± 0.004 on day 2, *P* < 0.0001; Fig. 4f,g and Extended Data Fig. 7m). Switching the sipper that was paired with optogenetic stimulation again reversed the sipper preference to the other side (Fig. 4f,g and Extended Data Fig. 7m). These changes in sipper preference occurred without any changes in total intake (Fig. 4g and Extended Data Fig. 7n,o), indicating that activation of these cells does not produce lasting satiety or aversion.

Pairing one sipper with closed-loop silencing of PRLH neurons produced the opposite result, such that animals preferentially drank out of the sipper coupled to silencing without any change in total consumption (Fig. 4h and Extended Data Fig. 7p–r). We observed a similar response to closed-loop silencing during sucralose ingestion, whereas silencing during water consumption had no effect (consistent with the activation of PRLH neurons by sucralose but not water ingestion; Extended Data Fig. 7s,t). Taken together, these data support a model in which PRLH neurons are activated by food tastes, which in turn rapidly modulates food palatability, thereby restraining the pace of ingestion. This negative feedback function would operate in parallel with, and partially counteract, the well-known effect of appetitive tastes to promote food consumption.

## GCG neurons track visceral feedback

The fact that PRLH neurons control ingestion on a seconds timescale in response to gustatory cues raises the question of which cNTS cells regulate ingestion in response to GI feedback, which is the traditional function ascribed to this structure^1–4^. To address this question, we examined GCG neurons, a distinct cell type that has been extensively studied alongside PRLH neurons for its role in non-aversive satiety^10,11,13,32^. GCG neurons express the anorexigenic peptide GLP-1 (ref. ^13^), are activated by ingestion (as measured by *Fos* staining)^10,33^ and inhibit food intake when stimulated without inducing conditioned taste avoidance^13,32^.

**Fig. 5:**
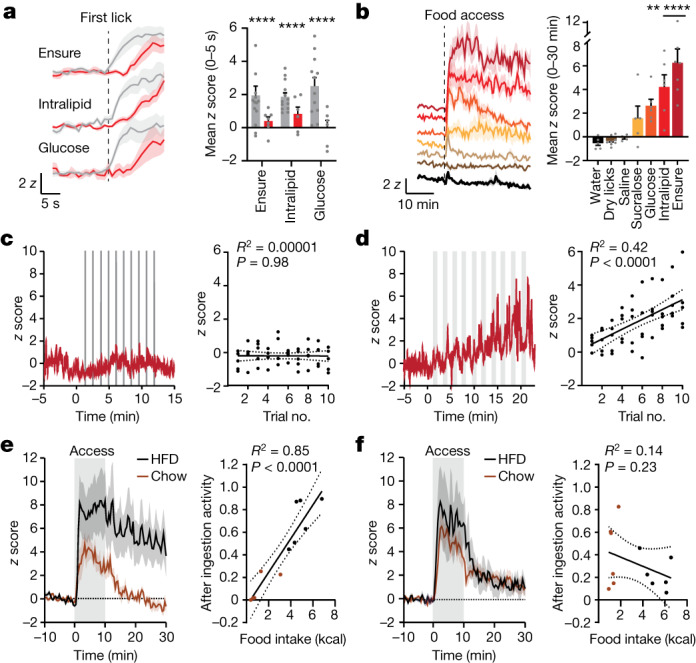
GCG neurons track cumulative intake on a timescale of minutes. **a**, Left, PRLH (grey) and GCG (red) neuron responses during the first lick bout. Right, *z* scores (0–5 s after the first lick in the first bout) Statistical comparisons are relative to the baseline prior to licking. **b**, Left, GCG neuron responses aligned to lickometer access for all tastants. Right, *z* scores (0–30 min). **c**, Mice were given access to Ensure at defined time intervals. Left, example trace of calcium dynamics from GCG neurons during 5 s of access to Ensure every 60 s (10 trials). Right, *z* scores during each brief access (0–5 s) versus the number of trials. **d**, Left, example trace of calcium dynamics from GCG neurons during 60 s of brief access to Ensure every 2 min (10 trials) with 60-s interbout. Same animal from **c**. Right, *z* scores during each brief access (0–60 s) versus the number of trials. **e**, Left, GCG neuron responses in mice given access to chow or a HFD from 0 to 10 min (grey). Right, correlation between total food intake during 10 min of access (kcal) and post-ingestive activity. Post-ingestive activity = mean *z* score after food removal (10–30 min)/mean *z* score during food access (0–10 min). **f**, Left, PRLH neuron responses in mice given access to chow or a HFD (0–10 min). Right, correlation between total food intake during 10 min of access (kcal) and post-ingestive activity. *P < 0.05, **P < 0.01, ***P < 0.001, ****P < 0.0001. Data are the mean ± s.e.m. Statistics are shown in Supplementary Table 1.

**Extended Data Fig. 8:**
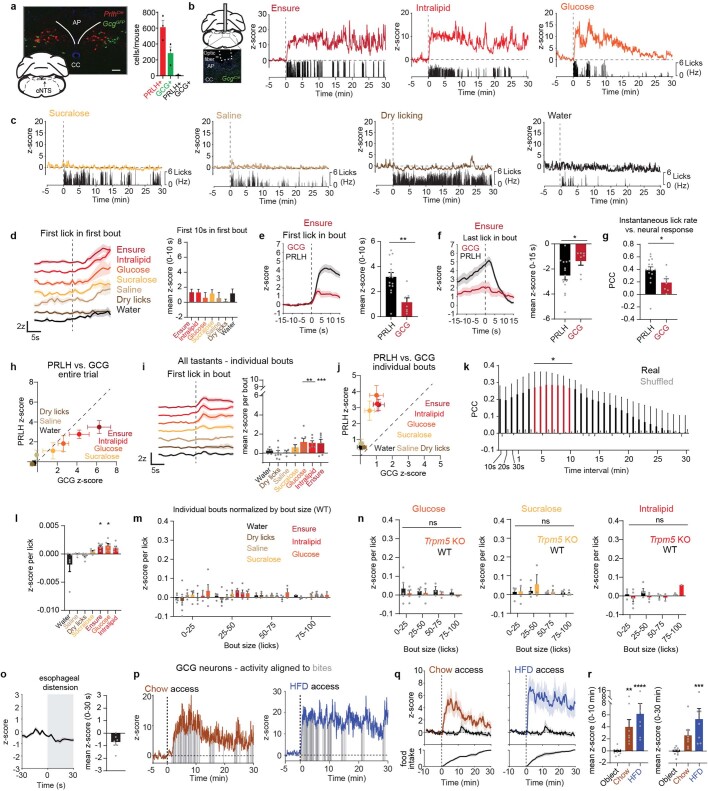
Regulation of GCG neurons by oral signals of ingestion. **a**, Left, PRLH (*Prlh*^*Cre*^*Rosa*^*Tom*^, red) and GCG (*Gcg*^*GFP*^, green) neurons are intermingled but non-overlapping in the cNTS. Scale bar = 100 µm. Right. Quantification of overlap for *Prlh*^*Cre*^*Rosa*^*Tom*^ (red) and *Gcg*^*GFP*^ (green) cells in the cNTS (n = 3 mice). **b**, Left, fiber placement and GcaMP6s expression in GCG neurons. Right, example traces of calcium dynamics from GCG neurons during self-paced Ensure, Intralipid, or glucose consumption. **c**, Example traces of calcium dynamics from GCG neurons during self-paced sucralose, saline, or water consumption, or dry licking at an empty sipper. **d**, Left, PSTH of GCG neuron responses during just the first lick bout of the trial, aligned to the first lick of the trial, for the indicated solutions. Right, mean z-scores (0–10 s) during the first lick bout. **e**, Left, PSTH aligned to the first lick of the bout (averaged across all lick bouts) during Ensure consumption for GCG and PRLH neurons. Right, mean z-scores (0–10 s) during Ensure consumption for GCG and PRLH neurons. **f**, Left, PSTH aligned to the last lick of the bout (averaged across all lick bouts) during Ensure consumption for GCG and PRLH neurons. Right, mean decrease in z-score (0–15 s after last lick of each bout) during Ensure consumption for GCG and PRLH neurons. **g**, Mean Pearson Correlation Coefficient (PCC) for the relationship between the instantaneous lick rate each second during Ensure consumption and the z-scored change in activity across the entire 30 min trial for GCG and PRLH neurons. **h**, Comparison of the mean z-scores (0–30 min after lickometer access) for PRLH neurons (from Fig. 1) and GCG neurons (from Fig. 5). **i**, Left, PSTH of GCG neuron activity aligned to the first lick (averaged across all lick bouts). Right, mean response (0–10 s after first lick) averaged across all licking bouts. **j**, Comparison of the mean z-scores (0–10 s after first lick in bout) for PRLH neurons (from Fig. 2) and GCG neurons (from Fig. 5). **k**, Mean PCC for the relationship between the cumulative licks performed in the preceding time intervals and the z-scored change in activity across the entire 30 min trial of self-paced Ensure consumption. **l**, Mean z-score per lick (mean z-score over 30 min trial/total number of licks) for each tastant. **m**, Mean z-score per lick (mean z-score 0–10 s of each bout divided by the number of licks in the same time frame) stratified by bout size for all tastants from GCG neurons. Each data point is an averaged value from a single animal. **n**, Left, mean z-score per lick by bout size for glucose consumption in WT and *Trpm5*^*−/−*^ mice (GCG neurons). Middle, mean z-score per lick by bout size for sucralose consumption in WT and *Trpm5*^*−/−*^ mice. Right, mean z-score per lick by bout size for Intralipid consumption in WT and *Trpm5*^*−/−*^ mice. **o**, Left, PSTH of GCG neuron responses during feeding tube insertion into the esophagus (0–30 s). Right, mean z-scores (0–30 s) during feeding tube insertion. **p**, Left, example trace of calcium dynamics from GCG neurons during self-paced chow consumption. Right, example trace of calcium dynamics from GCG neurons during self-paced HFD consumption. **q**, Top, PSTH of PRLH neuron responses during self-paced chow or HFD consumption, or presentation of a non-food object (black), aligned to moment of food presentation. Bottom, cumulative fraction of total bites during the trial. **r**, Left, mean z-scores (0–10 min) after food/object access. Right, mean z-scores (0–30 min) after food/object access. NS, *P < 0.05, **P < 0.01, ***P < 0.001, ****P < 0.0001. Data are mean ± sem. Statistics are shown in Supplementary Table 1.

**Extended Data Fig. 9:**
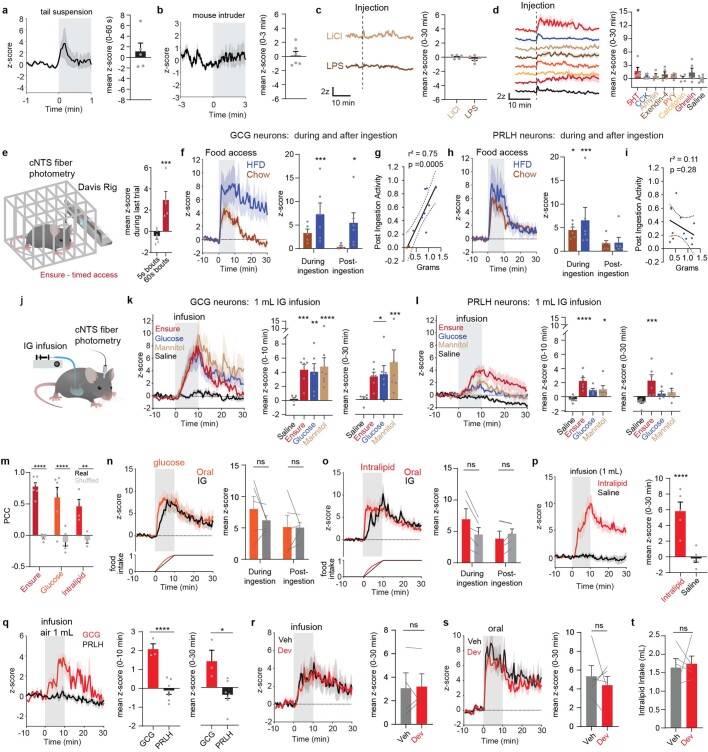
Regulation of GCG neurons by non-ingestive and GI signals. **a**, Left, PSTH of GCG neuron responses to tail suspension. Right, mean z-scores (0–60 s) during tail suspension. **b**, Left, PSTH of GCG neuron responses to presentation of a same-sex mouse intruder. Right, mean z-scores (0–3 min) during intruder presentation. **c**, Left, PSTH of GCG neuron responses to IP injection of LiCl (84 mg/kg) or LPS (100 ug/kg). Right, mean z-scores (0–30 min) after IP injection of LiCl or LPS. **d**, Left, PSTH of GCG neuron responses to IP injection of 5HT, CCK, amylin, Exendin-4, PYY, calcitonin, ghrelin, and saline. Right, mean z-scores (0–30 min) after IP injection of each gut peptide. **e**, A Davis Rig gustometer was used to give mice access to Ensure at defined time intervals. Right, mean z-score during the last trial of 5 s or 60 s brief access Davis rig experiments. **f**, Left, PSTH of GCG neuron responses in mice given access to chow or HFD from 0–10 min (gray shaded). Right, mean z-scores during 0–10 min food access (“during ingestion”) or 10–30 min (“post ingestion”). **g**, There is a positive correlation between total food intake during 10 min access (grams) and post-ingestive activity of GCG neurons. Post-ingestive activity was calculated as the average z-score after food removal (10–30 min) divided by the average z-score during food access (0–10 min). Colored dots indicate chow (brown) or HFD (blue). **h**, Left, PSTH of PRLH neuron responses in mice given access to chow or HFD from 0–10 min (gray shaded). Right, mean z-scores during 0–10 min food access (“during ingestion”) or 10–30 min (“post ingestion”). **i**, There is no correlation between total food intake during 10 min access (grams) and post-ingestive activity of PRLH neurons. **j**, cNTS photometry recordings were performed while mice received intragastric (IG) infusions of various solutions. **k**, Left, PSTH of GCG neuron responses during and after IG infusions (infusion 0–10 min; 1 mL) of Ensure, glucose, mannitol, or saline. Middle, mean z-scores during IG infusions (0–10 min). Right, mean z-scores during and after IG infusions (0–30 min). **l**, Left, PSTH of PRLH neuron responses during after IG infusions (0–10 min; 1 mL) of Ensure, glucose, mannitol, or saline. Middle, mean z-scores during IG infusions (0–10 min). Right, mean z-scores during and after IG infusions (0–30 min). **m**, Mean PCC for the relationship between the volume infused (1 mL) over time and the z-scored change in activity during the 10 min IG infusion of Ensure, glucose, or Intralipid. **n**, Left, PSTH of GCG neuron responses to volume-matched oral ingestion or IG infusion of glucose. Bottom panel shows mean trace for percentage of total food consumption. Right, mean z-scores during oral ingestion or IG infusion (0–10 min), or post-ingestion (10–30 min). **o**, Left, PSTH of GCG neuron responses to volume-matched oral ingestion or IG infusion of Intralipid. Right, mean z-scores during oral ingestion or IG infusion (0–10 min), or post-ingestion (10–30 min). **p**, Left, PTSH of GCG neuron responses to IG infusion of Intralipid or saline (1 mL) in *Trpm5*^*−/−*^ mice. Right, mean z-scores (0–30 min) after infusion of Intralipid or saline. **q**, Left, PTSH of PRLH and GCG neuron responses to IG infusion of air (1 mL) from 0–10 min. Right, mean z-scores during IG infusion of air (0–10 min) or during the entire trial (0–30 min). **r**, Left, PSTH of GCG neuron responses during and after IG infusions of Intralipid (0–10 min; 1.5 mL) in mice that received prior IP injection of devazepide or vehicle. Right, mean z-scores during and after IG infusions (0–30 min). **s**, Left, PSTH of GCG neurons during and after oral consumption of Intralipid, following injection of either devazepide or vehicle. Right, mean z-scores (0–30 min) during Intralipid consumption. **t**, Devazepide pretreatment does not change total Intralipid consumption in GcgiCre mice (from panel r). NS, *P < 0.05, **P < 0.01, ***P < 0.001, ****P < 0.0001. Data are mean ± sem. Statistics are shown in Supplementary Table 1.

GCG neurons were spatially intermingled with PRLH neurons in the cNTS but did not overlap (290 ± 74 cells per mouse; Extended Data Fig. 8a). We prepared mice for photometry recordings of GCG neurons and measured neural responses to Ensure consumption (Fig. 5a,b and Extended Data Fig. 8a, b). In contrast to PRLH neurons, which were activated coincident with the first lick, GCG neurons responded after a delay of several seconds (Fig. 5a, Extended Data Fig. 8d and Supplementary Video 8), with ramping activation (*T*_50_ = 2.4 ± 0.9 min) that then remained above baseline for the duration of the session (6.3 ± 1 *z* across the 30-min trial, *P* < 0.0001; Fig. 5b). A similar sustained activation was observed following consumption of glucose, Intralipid, chow or HFD, but not in response to non-food or aversive stimuli (Fig. 5b and Extended Data Figs. 8b–d,o–r and 9a–c). Thus, GCG neurons are strongly and specifically activated by consumption of food.

Several lines of evidence indicated that, in contrast to PRLH neurons, GCG neuron activity is not driven primarily by gustatory or other pre-gastric cues. First, the time-locked activation of GCG neurons during each lick bout was weaker than for PRLH neurons for all liquid diets tested (Extended Data Fig. 8e–g and Supplementary Videos 3 and 9). Second, GCG neurons were not activated by ingestion of sucralose, and their activation by glucose was not impaired in taste-blind mice (Fig. 5b and Extended Data Fig. 8h–j,l–n), which indicates that taste is neither necessary nor sufficient. Indeed, GCG neuron activity was most strongly correlated with Ensure consumption over longer timescales (4–10 min; Extended Data Fig. 8k), which suggests that these cells are regulated by feedback from the stomach and intestines.

We performed two experiments to test the hypothesis that GCG neurons track cumulative food intake on a timescale of minutes. First, we controlled the rate at which mice were allowed to ingest Ensure and then measured the neural response to different ingestion volumes (Fig. 5c,d). Consumption of Ensure for 5 s every 60 s (repeated 10 times) failed to activate GCG neurons (Fig. 5c), a result that was in contrast to the strong activation of PRLH neurons in similar brief-access taste tests (Fig. 3). However, increasing the access duration to 60 s resulted in a clear ramping activation of GCG neurons that correlated with the amount consumed (*R*^2^ = 0.42, *P* < 0.0001; Fig. 5d and Extended Data Fig. 9e), suggesting that they are progressively activated by GI fill. Second, to characterize how post-prandial GCG neuron activity relates to the amount of food consumed, fasted mice were given access to either chow or HFD for 10 min, and GCG neuron responses were measured during and after consumption (Fig. 5e). Post-ingestive activity of GCG neurons scaled linearly with the amount of food consumed during the preceding 10 min of access, confirming that these cells track cumulative food intake (*R*^2^ = 0.85, *P* < 0.0001; Fig. 5e and Extended Data Fig. 9f,g). By contrast, there was no correlation between the post-ingestive activity of PRLH neurons and the amount consumed, consistent with the fact that PRLH neurons track short-term orosensory cues (Fig. 5f and Extended Data Fig. 9h,i).

To confirm the sufficiency of GI signals for GCG neuron activation, we performed i.g. infusions of nutritive solutions, which triggered a strong, ramping activation of GCG neurons that correlated with the amount infused (Extended Data Fig. 9j–m). Of note, we observed robust responses in GCG neurons to infusions of only 1.0 ml, whereas PRLH neuron responses at this volume were weak (Extended Data Fig. 9j–l,p), indicating that GCG neurons are inherently more sensitive to GI feedback. Furthermore, GCG neuron responses to nutrient infusion into the stomach closely resembled GCG neuron responses to the same nutrient consumed by mouth (Extended Data Fig. 9n,o), whereas PRLH neurons showed substantial differences depending on the route of ingestion (Fig. 1). Thus, GI feedback is sufficient to explain the activation of GCG neurons during natural ingestion.

The activation of GCG neurons by post-ingestive feedback could be due to signals of GI stretch, nutrient sensing or both^5,24^. To test the sufficiency of GI stretch, we infused into the stomach the non-nutritive sugar mannitol, which is not absorbed and therefore induces significant intestinal distension^34^. Mannitol infusion strongly activated GCG neurons (5.0 ± 1.7 *z*, *P* = 0.0002), but not PRLH neurons (0.6 ± 0.6 *z*, *P* = 0.71; Extended Data Fig. 9j–l). Moreover, i.g. infusions of air (1.0 ml), a pure mechanosensory signal, also activated GCG neurons but not PRLH neurons (Extended Data Fig. 9q). In contrast to these strong responses to distension, GCG neurons were broadly insensitive to gut peptides released in response to intestinal nutrients, including CCK (Extended Data Fig. 9d,r–t). These data indicate that GCG neurons respond preferentially to GI stretch, although a modulatory role for nutritive signals is possible.

## GCG neurons promote long-lasting satiety

**Fig. 6:**
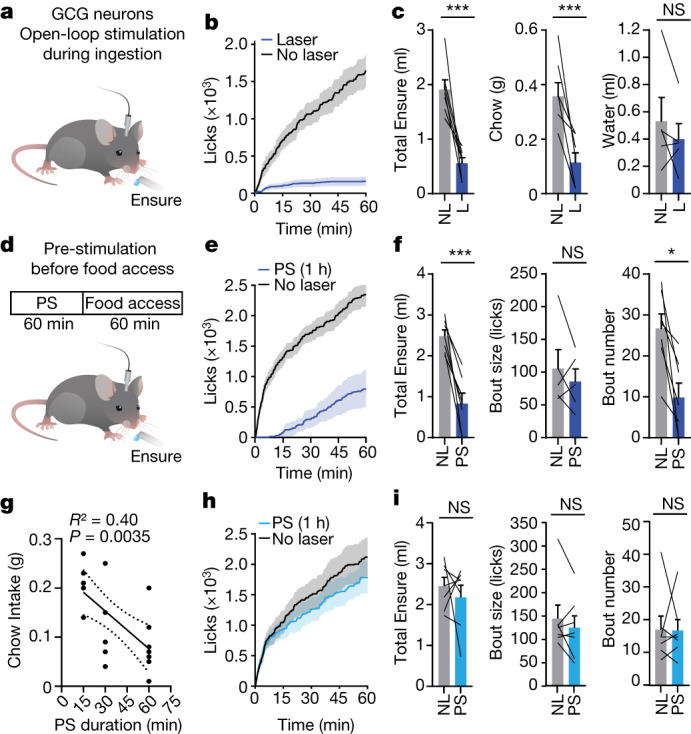
GCG neuron activation promotes long-lasting satiety. **a**, Schematic of experiment. Mice received continuous stimulation of GCG neurons expressing ChR2. **b**, Cumulative licks of Ensure during open-loop stimulation (60 min) of GCG neurons. **c**, Left, Ensure consumption in **b**. Middle, chow consumption during open-loop stimulation (30 min; food-deprived mice). Right, water consumption during open-loop stimulation (30 min; water-deprived mice). **d**, Schematic of experiment. Mice were pre-stimulated (PS) in the absence of food (60 min) and then given access to Ensure (60 min). **e**, Cumulative licks of Ensure after pre-stimulation (60 min) of GCG neurons. **f**, Left, Ensure consumption in **e**. Middle, bout size (two animals consumed zero bouts after pre-stimulation and therefore were not included for bout size analysis). Right, bout number. **g**, Negative correlation between total chow intake after pre-stimulation and the pre-stimulation duration. **h**, Cumulative licks of Ensure after pre-stimulation (60 min) of PRLH neurons. **i**, Left, Ensure consumption in **h**. Middle, bout size. Right, bout number. *P < 0.05, **P < 0.01, ***P < 0.001, ****P < 0.0001. Data are mean ± sem. Statistics are shown in Supplementary Table 2. Genotype controls (no opsin or Cre ± laser) for all experiments are shown in Extended Data Fig. 10.

**Extended Data Fig. 10:**
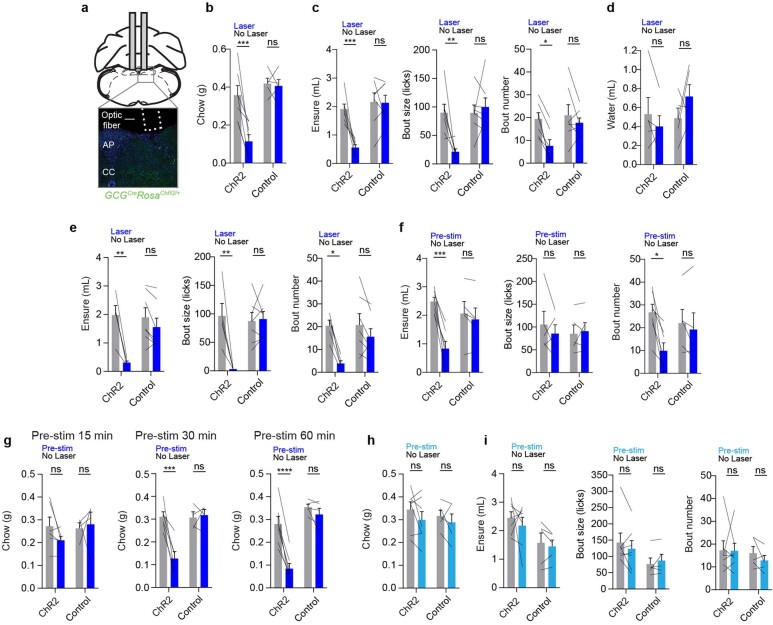
GCG neuron activation promotes long-lasting satiety. This figure extends the data from Fig. 6 by showing, for each experiment, the response of genetic controls (“controls” - mice that lack opsin or iCre expression) to the given laser stimulation protocol. The data from opsin-expressing mice (“ChR2”) are also reproduced here to enable direct comparison with controls. **a**, Fiber placement and ChR2-GFP expression in GCG neurons of the cNTS. **b**, Mean chow consumption (g) after open-loop stimulation (30 min; food-deprived mice) of GCG neurons or no laser trials for ChR2-expressing mice or control mice. **c**, Left, mean Ensure consumption (mL) after open-loop stimulation (60 min; dark phase) of GCG neurons or no laser trials. Middle, mean bout size (licks) after open-loop stimulation. Right, mean bout number after open-loop stimulation. **d**, Mean water consumption (mL) after open-loop stimulation (30 min; water-deprived) of GCG neurons or no laser trials. **e**, Left, mean Ensure consumption (mL) after closed-loop stimulation (60 min; dark phase) of GCG neurons (when animals are licking) or sham trials. Middle, mean bout size (licks) after closed-loop stimulation. Right, mean bout number after closed-loop stimulation. **f**, Left, mean Ensure consumption (mL) after pre-stimulation (60 min) of GCG neurons or no laser trials. Middle, mean bout size (licks) after pre-stimulation. Right, mean bout number after pre-stimulation. **g**, Mean chow consumption (g) after pre-stimulation of GCG neurons for 15 min (left), 30 min (middle), or 60 min (right). **h**, Mean chow consumption (g) after pre-stimulation of PRLH neurons for 60 min. **i**, Left, mean Ensure consumption (mL) after pre-stimulation (60 min) of PRLH neurons or no laser trials. Middle, mean bout size (licks) after pre-stimulation. Right, mean bout number after pre-stimulation. NS, *P < 0.05, **P < 0.01, ***P < 0.001, ****P < 0.0001. Data are mean ± sem. Statistics are shown in Supplementary Table 2.

Continuous optogenetic stimulation of GCG neurons inhibited the consumption of solid and liquid food but not water (Fig. 6a–c and Extended Data Fig. 10a–d), confirming that these neurons are involved in regulating feeding but not drinking. The fact that GCG neuron activity was not strongly time-locked to bouts of ingestion suggests that, unlike PRLH neurons, these cells do not specifically control the moment-by-moment dynamics of feeding. Indeed, closed-loop stimulation of GCG neurons during licking not only inhibited ongoing consumption but also reduced the initiation of later bouts (Extended Data Fig. 10e).

This long-lasting effect, combined with the observation that GCG neurons remain activated throughout feeding (Fig. 5), raises the possibility that GCG neuron activity may be integrated over time to influence the duration of post-prandial satiety. To test this idea, we used a pre-stimulation protocol^35,36^ (Fig. 6d) in which we stimulated GCG neurons in the absence of food (thereby mimicking the activation that would occur during and immediately after a meal), then turned the laser off and made food available. Pre-stimulation of GCG neurons (1 h) caused a strong reduction in subsequent food intake that persisted long after the offset of laser stimulation (2.5 ± 0.1 ml without pre-stimulation compared to 0.8 ± 0.2 ml with pre-stimulation, *P* = 0.0008; Fig. 6e,f and Extended Data Fig. 10f). This long-lasting effect was due to a reduction in the initiation of feeding bouts (Fig. 6f and Extended Data Fig. 10f), with no effect on bout size. This effect was observed with both solid and liquid food and, importantly, was dose-dependent, with longer pre-stimulation causing greater inhibition of subsequent feeding (Fig. 6g and Extended Data Fig. 10g). By contrast, pre-stimulation of PRLH neurons had no effect in any feeding assay (Fig. 6h,i and Extended Data Fig. 10h,i), confirming that PRLH neurons control behaviour on shorter timescales.

## Discussion

**Extended Data Fig. 11:**
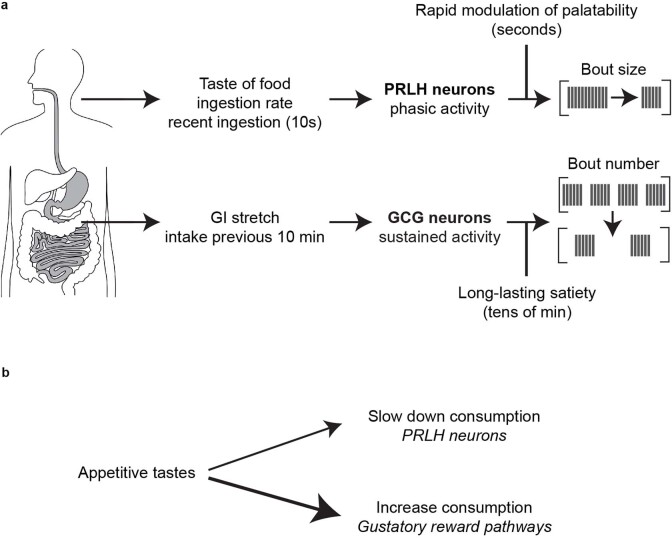
Separate circuits for the oral and gastrointestinal control of ingestion. **a**, Food intake generates both fast orosensory and slower GI signals that feed back to the cNTS to modulate appetite. Orosensory signals, including taste, preferentially target PRLH neurons, which are phasically activated during bouts of ingestion and function to acutely restrain bout size, thereby slowing down the pace of ingestion. Mechanosensory signals from the GI tract preferentially target GCG neurons, which show sustained activity during feeding and transmit a long-lasting satiety signal that delays reinitiation of feeding. **b**, Our data suggest that appetitive tastes, such as sweet and fat, are used by different brain systems for opposing purposes. Activation of well-known gustatory reward pathways by palatable tastes functions to increase food consumption. In parallel, activation of PRLH neurons by palatable tastes feeds back to slow down the rate of ingestion by limiting bout size. Although it may seem counterintuitive that palatable tastes would be used by some brain systems to inhibit ingestion, the existence of this mechanism is supported by several lines of evidence. This evidence includes the results of sham feeding studies in rats^41,42^, which showed that a pre-gastric signal (likely involving taste) slows down ingestion.

The cNTS is the first site in the brain where many meal-related signals are sensed and integrated, including almost all GI signals transmitted by the vagus nerve. Thus, it is important to establish how ingestive feedback is represented in the cNTS and used to control behaviour. Here we focused on PRLH and GCG neurons, which are the two principal cell types in the cNTS that have been implicated in non-aversive suppression of feeding^10–17^ (Extended Data Fig. 1). PRLH neuron activity was synchronized to bouts of ingestion and controlled the duration of  seconds-timescale feeding bursts, whereas GCG neurons were activated by slower post-ingestive feedback and promoted satiety that lasts for tens of minutes. These findings reveal that negative feedback signals from the mouth and gut engage genetically distinct circuits in the caudal brainstem, which in turn control feeding behaviour operating on short and long timescales (Extended Data Fig. 11).

PRLH neurons receive abundant feedback from the vagus nerve^14,20^ and remain activated for tens of minutes after nutrient infusion into the stomach, but this sustained activation by visceral feedback is substantially reduced during normal feeding. Instead, PRLH neuron activity is dominated by time-locked responses to orosensory cues, including taste. Because the cNTS does not receive direct gustatory feedback^1^, these orosensory signals are probably relayed by forebrain structures or premotor areas that innervate the cNTS^15^. Consistent with their primary regulation by orosensory rather than visceral cues, we found that PRLH neurons function to limit the size of ingestion bursts, with little effect on total intake, thereby restraining the pace of ingestion. This response may be important for preventing the GI distress that occurs when food is consumed too quickly^37^.

In contrast to PRLH neurons, GCG neurons responded strongly to mechanosensory feedback from the gut, consistent with results from *Fos* studies^5^ and rabies tracing^11^. The fact that optogenetic pre-stimulation of GCG neurons caused dose-dependent, long-lasting satiety suggests that release of GLP-1 can be integrated over time in downstream circuits to produce sustained reductions in appetite. A similar long-lasting effect may be important for the clinical efficacy of GLP-1 receptor agonists in reducing food intake^38^.

## Methods

Experimental protocols were approved by the Institutional Animal Care and Use Committee of the University of California, San Francisco, following the National Institutes of Health guidelines for the Care and Use of Laboratory Animals.

### Mouse strains

Experimental animals (>6 weeks old, both sexes) were maintained in temperature-controlled and humidity-controlled facilities with a 12-h light–dark cycle and ad libitum access to water and standard chow (PicoLab, 5053). The following mice were obtained from the Jackson Laboratory: WT (C57BL/6J; 000664); *Gcg*^*icre*^ (B6;129S-Gcg^tm1.1(iCre)Gkg/J^, 030663); Ai14 (B6.CgGt(ROSA)26 Sor^tm14(CAGtdTomato) Hze^/J; 007914), Ai213 (B6; 129S6-Igs7^tm213(CAG-EGFP,CAG-mOrange2,CAGmKate2) Hze^/J; 034113); *Trpm5*^−/−^ (B6.129P2-Trpm5^tm1Dgen/J^; 005848); Ai32 (B6.Cg-Gt(ROSA)26Sor^tm32(CAG-COP4*H134R/EYFP)Hze^/J; 024109); and R26-LNL-GtACR1-Fred-Kv2.1 (B6;129S6-Gt(ROSA)26Sor^tm3Ksvo/J^; 033089). GCG–GFP mice were a gift from H. Yoshitaka^39^. Nano-L10 mice have been previously described^40^. *Dbh*^*2A-FlpO*^ (B6.129S7(FVB) Dbh^em2.1(flpo)Rray/^Mmucd) mice were obtained from MMRRC (041575-UCD). *Prlh*^*cre*^ knock-in mice were crossed to Ai14, GCG–GFP and Nano-L10 mice to generate quadruple mutants (Extended Data Fig. 8a). *Prlh*^*cre*^ knock-in mice were crossed with *Dbh*^*2A-FlpO*^ and Ai213 mice to generate triple mutants (Extended Data Fig. 2g). *Prlh*^*cre*^ knock-in mice or *Gcg*^*icre*^ mice were crossed with *Trpm5*^−/−^ mice to generate triple mutants (*Prlh*^*cre*^*Trpm5*^−/−^ and *Gcg*^*icre*^*Trpm5*^−/−^ mice). *Prlh*^*cre*^ knock-in mice were crossed with R26-LNL-GtACR1-Fred-Kv2.1 mice to generate double mutants (*Prlh*^*cre/+*^*Rosa*^*GtACR1*/+^ mice). *Prlh*^*cre*^ knock-in mice or *Gcg*^*icre*^ mice were crossed with Ai32 mice to generate double mutants (*Prlh*^*cre/+*^ *Rosa*^*ChR2/+*^ and *Gcg*^*icre/+*^*Rosa*^*ChR2/+*^ mice, respectively). All transgenic or knock-in mice used in these studies were on a pure C57BL/6J background, except for *Prlh*^*cre*^ mice, which were partially backcrossed to C57BL/6J (from FVB).

### Generation of *Prlh*^*cre*^ mice

*Prlh*^*cre*^ mice were generated by homologous recombination at the endogenous *Prlh* locus, aided by targeted CRISPR endonuclease activity. The targeting vector was constructed to contain a T2A-Cre cassette inserted immediately upstream of the endogenous stop codon, a 1 kb upstream homology arm and a 2 kb downstream homology arm. A sgRNA was selected (CAGCACTTTTATTAGATCAG) to introduce CRISPR double-strand breaks near the stop codon, and the corresponding PAM sequence was mutated in the targeting vector (AGG to AGC) to prevent vector cleavage. Super-ovulated female FVB/N mice were mated to FVB/N stud males, and fertilized zygotes were collected from oviducts. Cas9 protein (100 ng µl^–1^), sgRNA (250 ng µl^–1^) and targeting vector DNA (100 ng ml^–1^) were mixed and injected into the pronucleus of fertilized zygotes. Zygotes were implanted into oviducts of pseudopregnant CD1 female mice. Screening of the pups by qPCR identified five independent founder lines that contained insertion of Cre but not sequences from the targeting vector (that is, knock-ins). These founders were crossed to Ai14 reporter mice, and all five lines showed a brain-wide recombination pattern that was identical to previous reports of *Prlh* expression (that is, restricted to the NTS, lateral reticular nucleus (LRt) and dorsomedial hypothalamus (DMH)) (Extended Data Fig. 2a–d). One *Prlh*^*cre*^ line was selected to maintain and further characterized by showing that recombination in the cNTS was highly overlapping with tyrosine hydroxylase (TH) and dopamine beta-hydroxylase (DBH) but not GCG neurons, as previously reported (Extended Data Fig. 2e–g and Extended Data Fig. 8a).

### Intracranial surgery

#### General procedures

Animals were anaesthetized with 2% isoflurane and placed in a stereotaxic head frame on a heating pad. Ophthalmic ointment was applied to the eyes and subcutaneous injections of meloxicam (5 mg kg^–1^) and sustained-release buprenorphine (1.5 mg kg^–1^) were given to each mouse before surgery. The scalp was shaved, scrubbed (betadine and alcohol three times), local anaesthetic applied (bupivacaine 0.25%) and then an incision was made through the midline. A craniotomy was made using a dental drill (0.5 mm). Virus was injected at a rate of 150 nl min^–1^ using a glass pipette connected to a 10 µl Hamilton syringe (WPI), controlled using a Micro4 microsyringe pump controller (WPI). The needle was kept at the injection site for 2 min before withdrawal. Fibre optic cannulas or a GRIN lens were implanted after virus injection during the same surgery, and these were secured to the skull using Metabond (Patterson Dental Supply, 07-5533559, 07-5533500; Henry Schein, 1864477) and Flow-It (Patterson Dental Supply, 07-6472542).

#### Fibre photometry recordings in the cNTS

*Prlh*^*cre*^ (*n* = 31), *Prlh*^*ce*^*Trpm5*^−/−^ (*n* = 6), *Gcg*^*icre*^ (*n* = 12) and *Gcg*^*icre*^*Trpm5*^−/−^ (*n* = 5) mice were prepared for photometry recordings by injecting AAV1-CAG-Flex-GCaMP6s (400 nl; 1.7 × 10^13^ viral genome copies (vg) per ml; Addgene) or AAV8-Syn-DIO-GCaMP6s (300 nl; 4.7 × 10^13^ vg per ml; Janelia Vector Core) into the cNTS (1.3 mm anterior–posterior (AP), ±0.3 mm medial–lateral (ML) and −4.3 mm dorsal–ventral (DV) relative to the occipital crest with 20° in the AP direction). In the same surgery, an optic fibre (Doric Lenses, MFC_400/430- 0.48_6.5mm_MF2.5_FLT) and sleeve (Doric Lenses, SLEEVE_BR_2.5) were installed 0.1–0.15 mm above the injection site. Mice were allowed to recover for a minimum of 3 weeks before the first photometry experiment. In subsequent surgeries, mice were equipped with an i.g. catheter.

#### Microendoscopy in the cNTS

*Prlh*^*cre*^ mice (*n* = 6) were prepared for imaging by injecting AAV1-CAG-Flex-GCaMP6s (200 nl; 1.5 × 10^12^ vg per ml; Addgene) into the cNTS (1.3 mm AP, ±0.3 mm ML and −4.3 mm DV relative to the occipital crest with 20° in the AP direction) and installing a GRIN lens (8 × 0.5 mm in length; Inscopix, 1050-004611) 0.15 mm above the injection site in the same surgery. After at least 2 weeks of recovery from the lens implantation surgery, mice were anaesthetized, and head bars were affixed to the skull using Metabond. A baseplate (Inscopix 100-000279) was placed above the lens and affixed using Metabond. When mice were not being used for imaging experiments, a baseplate cover (Inscopix 100-000241) was attached to prevent damage to the GRIN lens.

#### Optogenetics in the cNTS

*Prlh*^*cre/+*^, *Prlh*^*cre/+*^*Rosa*^*ChR2/+*^, *Gcg*^*icre/+*^, *Gcg*^*icre/+*^*Rosa*^*ChR2/+*^, *Rosa*^*ChR2/+*^ and *Prlh*^*cre/+*^*Rosa*^*GtACR1*/+^ mice were prepared for optogenetic experiments by installing a dual fibre optic cannula (Doric, DFC_200/245-0.37_6.5mm_DF0.8_FLT) above the cNTS (1.3 mm AP, 0 mm ML and −3.95 mm DV relative to the occipital crest with 20° in the AP direction). Mice were allowed to recover for a minimum of 1 week before optogenetic experiments.

### Intragastric catheter surgery

Mice were deeply anaesthetized with 2% isoflurane and surgical sites were shaved and cleaned with betadine and ethanol. Subcutaneous injections of meloxicam (5 mg kg^–1^) and sustained-release buprenorphine (1.5 mg kg^–1^) were given to each mouse before surgery. A midline abdominal skin incision was made, extending from the xyphoid process about 1.5 cm caudally, and a secondary incision of 1 cm was made between the scapulae for externalization of the catheter. The skin was separated from the subcutaneous tissue using blunt dissection, such that a subcutaneous tunnel was formed between the two incisions along the left flank to facilitate catheter placement. A small incision was made in the abdominal wall and the catheter (Instech, C30PU-RGA1439) was pulled through the intrascapular skin incision and into the abdominal cavity using a pair of curved haemostats. The stomach was externalized using atraumatic forceps and a purse string stitch was made in the middle of the forestomach using a 7-0 non-absorbable Ethilon suture. A puncture was then made in the centre of the purse string, and the end of the catheter was inserted and secured by the purse string suture. For the gastric implant, 2–5 mm of the catheter end was fixed within the stomach.

At the end of the surgery, the abdominal cavity was irrigated with 1 ml of sterile saline and the stomach was replaced. The abdominal incision was closed in two layers, and the catheter was sutured to the muscle layer at the interscapular site. The interscapular incision was then closed and the external portion of the catheter capped using a 22-gauge PinPort (Instech, PNP3F22). Mice received Baytril (5 mg kg^–1^) and warm saline at the end of surgery and were allowed to recover for 1 week before photometry experiments.

### Fibre photometry

#### Photometry setup

Mice were tethered to a patch cable (Doric Lenses, MFP_400/460/900-0.48_2m_FCM-MF2.5). Continuous 6 mW blue LED (470 nm) and UV LED (405 nm) served as excitation light sources. These LEDs were driven by a multichannel hub (Thorlabs), modulated at 305 Hz and 505 Hz, respectively, and delivered to a filtered minicube (Doric Lenses, FMC6_AE(400-410)_E1(450-490)_F1(500-540)_E2(550-580)_ F2(600-680)_S) before connecting through optic fibres (Doric Lenses, MFP_400/460/900-0.48_2m_FCM-MF2.5). GCaMP calcium GFP signals and UV isosbestic signals were collected through the same fibres back to the dichroic ports of the minicube into a femtowatt silicon photoreceiver (Newport, 2151). Digital signals sampled at 1.0173 kHz were then demodulated, lock-in amplified and collected through a processor (RZ5P, Tucker-Davis Technologies). Data were then collected using the software Synapse (TDT), exported using Browser (TDT) and downsampled to 4 Hz in MATLAB before analysis.

#### Behaviour

For all recordings, mice were placed in sound-isolated behavioural chambers (Coulbourn, Habitest Modular System; Med Associates, Davis Rig) without water or food access unless otherwise specified. Chambers were cleaned between experiments to remove olfactory cues from previous experiments. Mice were habituated for one night in the chambers before experiments. On the next day, mice were attached to photometry patch cords and habituated to the chambers for a second session. Before each recording, photometry implants on individual mice were cleaned with 70% ethanol using connector cleaning sticks (MCC-S25) and connected to a photometry patch cable immediately afterwards. For all photometry experiments, mice were acclimated to the behaviour chamber for 20 min with recording before presentation of a stimulus.

For i.g. infusion experiments, mice were deprived of food overnight before the experiment. Solutions—saline (0.9%), glucose (24%), Intralipid (20%), Ensure (21%), MDG (16%) or sucralose (6.25 mM)—were delivered using a syringe pump (Harvard Apparatus, 70–2001) over 10 min. The infusion rate was 100 μl min^−1^ or 150 μl min^−1^ for a total infusion volume of 1 or 1.5 ml, respectively. Before mice were placed into the Coulbourn behavioural chambers for habituation, the i.g. catheter was attached to the syringe pump using plastic tubing and adapters (AAD04119, Tygon; LS20, Instech).

For the lick response experiments, mice were deprived of food overnight before the experiments before receiving access to a lickometer containing the appropriate solution for the entire 30-min session. Solutions were prepared using deionized water at the following concentrations: 0.24 g ml^–1^ glucose (1.33 M, 24%); 0.009 g ml^–1^ saline (0.15 M, 0.9%); 0.21 g ml^–1^ Ensure original vanilla nutrition powder (21%); and 0.8 mg ml^–1^ sucralose (in saline). Intralipid 20% (Sigma, I141-100ML; Medline, BHL2B6064H) was used without dilution. To measure lick responses to Ensure in fed mice, ad libitum fed mice were given access to Ensure (21%) for 30 min in the dark phase (after 17:00). All mice were habituated initially to the lickometer and photometry setup by receiving access to a bottle containing Ensure for 1 h with the photometry patch cord attached.

For chow and HFD experiments, mice were deprived of food overnight before the experiment. Mice were then given access to a non-food object (metal cage), standard chow (PicoLab 5053) or a HFD (Research Diets, D12492) for the entire 30-min session, or for 10 min in restricted access experiments for chow and HFD. Bites—defined as individual time points when the mouse lowers its head to make contact with the food pellet—were manually scored by an experimenter blinded to the experimental conditions. Behavioural annotation was performed using behavioural observation research interactive software (http://www.boris.unito.it/).

For i.p. injection experiments, mice were injected with compounds at the following concentrations based on previously published reports: CCK octapeptide, 30 μg kg^–1^ (Bachem); devazepide, 1 mg kg^–1^ (R&D Systems); serotonin hydrochloride, 2 mg kg^–1^ (Sigma-Aldrich); PYY, 0.1 mg kg^–1^ (R&D Systems); exendin-4, 150 μg kg^–1^ (Bachem); salmon calcitonin, 150 μg kg^–1^ (Bachem); amylin, 10 μg kg^–1^ (Tocris); ghrelin, 2 mg kg^–1^ (R&D Systems); LiCl, 84 mg kg^–1^; and LPS, 100 µg kg^–1^. All of these compounds were dissolved in saline (0.9%), except devazepide, which was dissolved in 5% DMSO, 5% Tween-80 in saline (vehicle solution for devazepide). All compounds were injected at a volume of 10 µl g^–1^ of mouse body weight.

For Intralipid and devazepide experiments, mice were deprived of food before the experiment. For the i.g. infusion experiment, after receiving an i.p. injection of vehicle or devazepide, mice were given an i.g. infusion of Intralipid (20%) over 10 min. The infusion rate was 150 μl min^−1^ for a total infusion volume of 1.5 ml. For the oral ingestion experiment, mice received an i.p. injection of vehicle or devazepide before receiving access to a lickometer containing Intralipid (20%) for 10 min (same time frame as the i.g. infusion experiment).

For volume-matched glucose or Intralipid experiments, mice were deprived of food before the experiment. On day 1, mice were given access to a lickometer containing glucose or Intralipid for 10 min before removal and an additional 20 min of photometry recording. Two days later (day 2 of the experiment), overnight-fasted mice were given an i.g. infusion of glucose or Intralipid over 10 min at the same volume that each animal individually consumed on day 1. In summary, mice received the same volume of glucose or Intralipid solution with the same timing on day 1 and day 2 through oral ingestion or i.g. delivery.

For oesophageal distension experiments, mice were deprived of food overnight before the experiment. Mice were scruffed and restrained for 30 s before a 24-gauge reusable feeding tube (FST, 18061-24) was inserted and held in the oesophagus for 30 s.

For lick response experiments comparing *Prlh*^*cre*^ and *Prlh*^*cre*^*Trpm5*^−/−^ mice or *Gcg*^*icre*^ and *Gcg*^*icre*^*Trpm5*^−/−^ mice, mice were deprived of food overnight before gaining access to a lickometer containing glucose (24%), sucralose (6.25 mM) or Intralipid (20%). All solutions were prepared using deionized water except for Intralipid, which was not diluted. *Prlh*^*cre*^*Trpm5*^−/−^ mice were initially habituated to the lickometer and photometry setup for three sessions across multiple days, in which they were given ad libitum access to water after being deprived of water overnight. This was performed to train the taste-blind mice to perform licks with the lickometer in subsequent experiments, as they perform fewer licks than WT mice at baseline. After this initial habituation procedure, taste-blind mice were still naive to glucose, sucralose and Intralipid. For post-ingestive training of taste-blind mice with glucose, we gave the animals ab libitum access to a bottle containing glucose (24%) overnight twice, with a day of separation between these two exposure periods. We defined taste-blind mice as ‘learned’ if they performed more than 1,000 licks during a second 30-min test with glucose. Naive WT mice performed at least 1,000 licks in a 30-min test with glucose, whereas all naive taste-blind mice performed fewer licks when food-deprived.

For brief access taste tests using the Davis Rig (MED-DAV-160M, Med Associates), mice were deprived of food overnight. Mice were given 5 and 60 s of lick access to a bottle of Ensure for ten total trials. In all experiments, the end of a trial and the beginning of the next trial were separated by 1 min. Before the photometry experiments, mice were initially habituated to the Davis Rig and photometry setup for three sessions across multiple days, during which they were given ad libitum access to Ensure after being deprived of food overnight.

#### Analysis

GCaMP6s calcium responses at 470 nm excitation were normalized to the 405/415 nm isosbestic signal using a linear regression model of both signals during the baseline period to generate *F*_normalized_ (the fluorescence predicted using the signal obtained with 405/415 nm excitation). Data were analysed using the function *z* = *(F*_normalized_ *–* *μ)/σ*, where *F*_normalized_ is the normalized photometry signal, *μ* is the mean *F*_normalized_ during the baseline period before stimulus presentation and *σ* is the standard deviation of *F*_normalized_ during the same baseline period. For example traces from individual mice, the *z*-score trace and lick rates were smoothed using a moving average filter with spans of 20 and 5 data points. For data presentation only, plotted mean traces (30 min) were additionally downsampled by a factor of 100 (this was done to decrease the size of each graph).

For most experiments, the baseline period was the 10 min before stimulus presentation, a period in which the mouse was left undisturbed in the behaviour chamber. The photometry data from this period were used to calculate the baseline activity, which was then compared with the average *z* score during the selected epoch after stimulus presentation.

To determine *T*_50_ values, we determined the earliest time point at which 50% of the maximum *z* score over the entire 30-min trial was attained, or the time point at which animals consumed at least 50% of the total food during the 30-min period. For i.g. infusions, the *T*_50_ value is always 5 min because infusions were performed from 0 to 10 min.

To calculate the PCC for relationship between two variables, we used the coorcoef function in MATLAB with the instantaneous lick rate (Hz), cumulative intake from 0 to 10 min (i.g. infusion) or the entire 30-min trial, or the cumulative licks over preceding time intervals (from 10 s to 30 min) as an input vector. The movsum function in MATLAB was used to calculate the total licks in different preceding time windows. The *z*-scored change in activity (compared with the 10-min baseline period) over 0–10 min (i.g. infusion) or 0–30 min (all other comparisons) was used as the other input vector. A PCC value was calculated for each animal. To calculate the PCC for the shuffled controls, the input vector for food intake was scrambled for each animal.

For analysis of photometry responses time-locked to licking, the 15 s before the start of each lick bout was used to calculate the baseline activity. A lick bout was defined as any set of licks that last 4 s or more and are separated from the previous lick bout by at least 20 s. The *z* score per bout was calculated as the average *z* score in the first 10 s of each individual licking bout. The *z* score per lick was calculated by dividing the *z* score per bout (defined above) by the number of licks in the first 10 s of that particular licking bout. To calculate the *z* score per lick for individual animals at different bout size bins, *z* score per lick values falling into each bin of 5–25 licks, 26–50 licks, 51–75 licks or 76–100 licks were averaged. For example, all values from bouts containing 26–50 licks from a single animal would be averaged into a single value and used for statistical analyses.

To determine the tau time constant across all Ensure lick bouts, we determined the earliest time point at which 63.8% of the maximum *z* score within a bout (averaged across all bouts) was reached. To determine the lambda decay constant across all Ensure lick bouts, we determined the earliest time point at which the *z* score was 37% of the value during the last lick of the bout (averaged across all bouts).

For comparing licking responses in the early or late portion of the 30-min trial, photometry data in the first 15 min (early) or last 15 min (late) were separated for subsequent analyses. The 15 s before the start of each lick bout was always used to calculate the baseline activity.

To calculate the mean *z* score per bout for the first bout to the last bout (Extended Data Fig. 4g–i), the neural response during that bout number was averaged across all mice. The maximum bout size (in licks) was always the first lick bout. The percentage of this maximum bout size, from 0 = 0% to 1 = 100%, was plotted for each bout below the corresponding bout. Because the number of bouts per experiment was variable, we limited the analysis to bouts for which data from multiple animals were available.

To calculate the slope (×1 coefficient), we used the fitlm function in MATLAB to assess the relationship between bout size (licks) and the *z* score in a bout for the first 10 s in a bout. The ×1 value is equal to the change in *z* score per bout for each additional lick, and this was obtained for each individual animal (Fig. 2h).

To train coefficients for GLMs, we use the fitglm function in MATLAB with caloric density (kcal ml^–1^; vector containing constant value), licks in the past 10 s (at each second of a 30-min trial) and instantaneous lick rate (at each second of a 30-min trial) as the predictor variables. Caloric densities were 2 kcal ml^–1^ for Intralipid, 0.923 kcal ml^–1^ for Ensure, 0.96 kcal ml^–1^ for glucose, and zero for saline, dry licks or water. The response variable was the *z*-scored change in activity (relative to a 10-min baseline before lick access) at each second of a 30-min trial. A GLM was built for each animal using all photometry data from 30-min trials of Ensure, glucose, Intralipid, saline and water consumption, and dry licking at an empty bottle. The adjusted *R*^2^ value from the GLM for each animal was used to determine the average fraction of the variance in the *z* score (*t*) explained by models with different subsets of variables. To calculate the contribution of each variable, the *R*^2^ value of each model without that variable was subtracted from the *R*^2^ value of the full model and then averaged. To perform cross-validation, we used 80% of the photometry data to train the GLM coefficients and calculate the mean squared error (MSE) from the remaining 20% of the data. This was performed for 100 iterations to obtain an average MSE value for each animal.

For oesophageal distension experiments, photometry data collected in the 60 s before feeding tube insertion were used to calculate the baseline activity.

To calculate the average drop in *z* score at the end of a lick bout (Extended Data Fig. 8f), we determined the difference between the peak *z* score within the 15 s before the last lick and the mean *z* score after the last lick: ((mean *z* score 0–15 s) – (peak *z* score – 15–0 s)).

For brief access Davis rig experiments with Ensure (Fig. 5), lick responses were calculated as the average *z*-scored change of activity in the 5 or 60 s following the first lick of a trial. Photometry data from the 10-min baseline period was used to calculate the baseline activity. To calculate the *R*^2^ value in this experiment, we analysed the relationship between the mean *z* score in each trial and the trial number using a linear regression. The *z*-scored change of activity was averaged across the last trial (tenth) to generate a mean *z*-score value (Extended Data Fig. 9e).

For the 10-min food access experiments, ingestive and post-ingestive responses were defined as the average z-scored change in activity during the 10-min access period and the 20-min period following ingestion, respectively. Post-ingestive activity was calculated as (post-ingestive response/ingestive response) × 100%. This was done to normalize for differences in photometry signal across individual mice. Food intake (kcal) was calculated as the mass of food consumed (g) multiplied by the caloric density (kcal g^–1^).

### Microendoscopy

#### Behaviour

To habituate mice to the imaging setup, we head-fixed mice using a custom-built stage (ThorLabs) before applying additional restraint by placing the animal in a 50 ml conical tube (Fisher Scientific, 14-432-22). A disposable fluff underpad (MSC281230) was used to reduce limb movement while ensuring that animals were comfortable under restraint. All mice were initially habituated to the imaging setup by receiving access to a bottle containing Ensure with the microendoscopy camera attached for two sessions (2 h each) on two separate days.

Mice were deprived of food overnight before brief access experiments. On the day of the experiment, mice were head-fixed and restrained using the method described above and given 10 min for habituation, with the Inscopix camera turned on. After an additional 10 min of baseline recording, mice received brief 5 s of access to a sipper containing the appropriate solution (Ensure, Intralipid, sucralose or water; same concentrations as for the photometry experiments) at 5-min intervals over 20 min.

For experiments involving CCK, mice were given brief 5 s of access to sucralose at 5-min intervals over 15 min before receiving a subcutaneous injection of CCK (30 µg kg^–1^) near the shoulder area. We continued the recording for an additional 15 min to measure the neural response to CCK.

#### Data collection and analysis

Data were collected using Inscopix nVista and nVoke microscopes. Videos were acquired at 20 Hz (20% LED power, 8.0 gain and 2× spatial downsampling) using Inscopix software (data acquisition software v.151; https://support.inscopix.com/support/products/nvista-30-and-nvoke-20/data-acquisitionsoftware-v151). After acquisition, videos were first pre-processed, spatially (binning factor of 2) and temporally (binning factor of 5) downsampled, and motion-corrected using Inscopix software (v.1.7; http://support.inscopix.com/mosaic-workflow). Videos underwent additional motion correction using Mosaic software (v.1.7; http://support.inscopix.com/mosaic-workflow), which produced estimates of the motion artefacts when mice were freely moving, head-fixed, or head-fixed and restrained (Fig. 3). Activity traces for individual neurons were then extracted from these videos using the constrained non-negative matrix factorization (CNMF-E) pipeline in the Inscopix software. After initial CNMF-E segmentation, extracted neurons were manually refined to avoid potential confounding factors from uncorrected motion artefacts, region of interest duplication and over-segmentation of the same spatial components.

For each experiment, activity traces for individual neurons were extracted for each mouse and all responses were normalized using the function *z* = *(C*_raw_ *–* *μ)/σ*, where *C*_raw_ is an output of the Inscopix software, *μ* is the mean *C*_raw_ during the 10-min baseline period before stimulus presentation and *σ* is the standard deviation of *C*_raw_ during the same baseline period.

To calculate the tau time constant for Ensure lick bouts, we determined the earliest time point at which 63.8% of the maximum *z* score within a bout (averaged across all bouts and all neurons for each animal) was reached.

For analysis of single-cell responses during lick bouts, the 15 s before the start of each lick bout was used to calculate the baseline activity, which was used to calculate the *z* score from 0 to 15 s. We defined a neuron as activated if the mean *z* score was ≥1 *z*. Neurons with a mean *z* score of <1 *z* were defined as non-responsive. To calculate the population-weighted *z* score, we multiplied the fraction of neurons activated across all 4 bouts (+1 *z*) by their *z*-scored activity change (averaged across all neurons). We calculated the *z* score during each lick bout by averaging the *z*-scored change in activity of all activated neurons within that particular lick bout. To calculate the percentage of neurons activated during a particular number of bouts, we determined whether individual cells were activated (>1 *z*) across all four bouts (all bouts), three bouts, two bouts or only one bout.

To compare the *z* score during sucralose consumption to the *z* score after i.p. injection of CCK, we first measured the average *z*-scored change in activity across all three sucralose lick bouts (*z* score during licking). Next, we calculated the average *z*-scored change in activity in the 15 min after i.p. injection of CCK (*z* score after injection). Cells were classified as responsive to sucralose-only if the mean *z* score during licking was >1 *z*, whereas the mean *z* score after injection was ≤1 *z*, CCK-only if the mean *z* score after injection was >1 *z*, whereas the mean *z* score after during licking was ≤1 *z*, both if the mean *z* score for both stimuli was >1 *z*, and none if the mean *z* score for both stimuli was ≤1 *z*. We used the fitlm function in MATLAB to calculate the *R*^2^ value between the *z* score during licking and the *z* score after injection.

### Optogenetics

#### Laser parameters

For continuous stimulation, closed-loop stimulation and pre-stimulation experiments, the laser was modulated at 20 Hz for a 2-s on and 3-s off cycle with a 10-ms pulse width. For closed-loop stimulation or silencing experiments during ingestion, the laser was modulated for 2 s after each lick detected using Graphic State software, which was synchronized with a contact lickometer. This closed-loop modulation was either at 20 Hz (stimulation) or continuous (inhibition). Closed-loop stimulation when the animal was not actively licking (lick-off) was performed at 20 Hz for a 2-s on and 3-s off cycle, and each new lick performed would turn off this modulation for 2 s. Photostimulation or photoinhibition was delivered using a DPSS 473-nm laser (Shanghai Laser and Optics Century BL473-100FC) through a dual fibre optic patch cord (Doric, DFP_200/220/900-0.37_2m_DF0.8-2FC0) at a laser power of 10–15 mW (photostimulation) or 5–6 mW (photoinhibition), which was measured at the tip of the patch cable before the experiments for each day.

#### Behaviour

All experiments were fully counterbalanced for the order of stimulation and contained both within animal (±laser) and genotype (±opsin) controls. Genotype controls were typically littermates that lacked either the Cre or reporter allele. For all experiments, mice were placed in sound-isolated behavioural chambers (Coulbourn, Habitest Modular System; Med Associates, Davis Rig) without water or food access unless otherwise specified. Chambers were cleaned between experiments to remove olfactory cues from previous experiments. Mice were habituated for one night in the chambers before experiments. On the next day, mice were attached to optogenetic patch cords and habituated to the chambers for a second session. On the day of experiments, mice were acclimated to the behaviour chamber for 10 min before optogenetic manipulation and/or food access.

For open-loop stimulation experiments measuring chow consumption, animals were deprived of food overnight before the experiment was performed in the light phase. After habituation, animals received a pellet of standard chow (PicoLab 5053) for self-paced consumption over 15, 30 or 60 min, depending on the experiment. Animals also received open-loop stimulation (described above) during the entire session or no laser treatment.

For open-loop stimulation experiments measuring water consumption, animals were deprived of water overnight before the experiment was performed in the light phase. After habituation, animals were given access to a bottle containing water for 30 min alongside open-loop stimulation.

For experiments measuring single-bottle consumption of Ensure and Intralipid, animals were ad libitum fed and the experiments were performed in the dark phase (after 17:00). After habituation, animals were given access to a bottle of Ensure or Intralipid for 1 h as they received open-loop stimulation, closed-loop stimulation (during licking only or while not licking) or closed-loop silencing, depending on the experiment.

For two-bottle preference experiments with Ensure, animals were ad libitum fed and the experiments were performed in the dark phase. On day 1, mice were given access to two identical bottles of Ensure on opposite ends of the behavioural chamber for 1 h of self-paced consumption. For closed-loop stimulation experiments, the more preferred bottle (more licks than the other bottle) was designated as bottle 1, whereas the less preferred bottle was designated a bottle 1 for closed-loop silencing experiments. On day 2 of the experiment (the subsequent day), mice were again given access to two identical bottles of Ensure, whereby bottle 1 was paired with closed-loop stimulation or silencing. On the next day, mice once again received access to two bottles of Ensure, but bottle 2 was now paired with closed-loop stimulation of silencing. The location of bottles 1 and 2 was not changed during the experiment.

For two-bottle preference experiments with water or sucralose, mice were ad libitum fed and experiments were performed in the dark phase. Mice received access to two bottles of the identical solution for 1 h, whereby one bottle was randomly paired with closed-loop silencing.

For pre-stimulation experiments with Ensure, ad libitum fed animals were habituated to the chambers in the dark phase before receiving optogenetic stimulation for 1 h. After the pre-stimulation ended, mice were then given a bottle of Ensure for 1 h of self-paced consumption. For pre-stimulation experiments with chow, mice were deprived of food before receiving 15, 30 or 60 min of pre-stimulation in the light phase. Next, mice received a pellet of standard chow for 15 min of self-paced consumption.

#### Analysis

To measure chow consumption, the pellet was weighed before and after the experiment. For Ensure and Intralipid intake measurements, a bottle of Ensure (0.21 g ml^–1^) or Intralipid (20%) was weighed before and after consumption. Excess spillage from the lickometer was collected and added to the bottle weight after consumption. Mass was converted to volume using the density of 21% Ensure (about 1.07 g ml^–1^) and Intralipid (about 1 g ml^–1^).

For licking bout analyses in optogenetic experiments, a licking bout was defined as any set of licks containing at least three licks, in which no inter-lick interval was greater than 5 s. Bout size was calculated as the average number of licks from all lick bouts in the 1 h of the dark phase session. Bout duration was calculated as the average length of all lick bouts in seconds. The bout number was the total number of bouts across the entire 1-h trial.

To measure the number of laser pulses received by individual mice, the bout duration (seconds) values from all lick bouts were summed to calculate the total time (seconds) each animal was consuming Ensure. To calculate the number of laser pulses in the lick-on test, the summed value was multiplied by 20 because the laser was modulated at 20 Hz. To calculate the number of laser pulses in the lick-off test, the total time licking (seconds) for each animal was subtracted from the full trial (60 min or 3,600 s) and then multiplied by 8 because the laser was modulated at 20 Hz for a 2-s on 3-s off cycle (or 40 pulses over 5 s).

To measure the interlick interval within a bout (Extended Data Fig. 7), we measured the time between each lick between 0 and 1 s. To calculate the Mu1 and Mu2 constants, we used the fitgmdist function in Matlab, with *k* = 2 components fitted to the data based on the two peaks observed in the probability mass function (Extended Data Fig. 8i,k).

### Histology

Mice were anaesthetized under isoflurane and then transcardially perfused with PBS (10 ml) followed by formalin (10%, 15 ml). Brains were dissected, post-fixed in 10% formalin overnight at 4 °C and switched to 30% sucrose the next day. All tissues were kept in 30% sucrose at 4 °C for overnight cryo-protection and embedded in OCT before sectioning. Sections (50 μm) were prepared using a cryostat and collected in PBS or on Superfrost Plus slides. To visualize fluorescent labelling without staining, sections were mounted with DAPI Fluoromount-G (Southern Biotech) and then imaged by confocal microscopy (Zeiss, LSM 510).

For immunostaining, sections (50 μm) were washed 3 × 10 min with 0.1% PBST (0.1% Triton X-100 in PBS), blocked (5% NGS or NDS in 0.1% PBST) for 30 min at room temperature and incubated with primary antibodies (1:1,000 diluted in blocking solution) overnight at 4 °C. The next day, sections were washed 3 × 10 min with 0.1% PBST, incubated with secondary antibodies (1:500 diluted in blocking solution) for 2 h at room temperature, washed again 3 × 10 min with 0.1% PBST and mounted with DAPI Fluoromount-G (Southern Biotech). Primary antibodies used were chicken anti-GFP (Abcam, ab13970, 1:1,000) and rabbit anti-TH (Millipore, AB152).

### Statistics

All values are reported as the mean ± s.e.m. (error bars or shaded areas). Sample size is the number of animal subjects per group. In figures, asterisks denote statistical significance: *P < 0.05, **P < 0.01, ***P < 0.001, ****P < 0.0001. In figures with simple linear regressions, dashed lines represent the 95% confidence interval for the line of best fit. Except for linear regressions or two-way analysis of variance (ANOVA), nonparametric tests were uniformly used. *P* values for paired or unpaired comparisons were calculated using the Wilcoxon signed-rank test or Mann–Whitney *U*-test and corrected for multiple comparisons using the Holm–Šidák multiple comparisons test. *P* values for comparisons across multiple groups were calculated using the Kruskal–Wallis test or two-way ANOVA and corrected for multiple comparisons using Dunn’s multiple comparisons test and Šidák’s or Dunnett’s multiple comparisons test, respectively. Fisher’s exact test was used to assess whether overlapping neural responses were due to chance. To compare between groups, data from each animal were averaged for biological replicates. See Supplementary Tables 1 and 2 for a complete summary of all statistics. No statistical method was used to predetermine sample sizes. Randomization and blinding were not used.

We analysed fibre photometry data, behaviour data and microendoscopy imaging data using custom Matlab (v.R2017a, http://www.Mathworks.com/products/matlab) scripts.

### Reporting summary

Further information on research design is available in the Nature Portfolio Reporting Summary linked to this article.

## Online content

Any methods, additional references, Nature Portfolio reporting summaries, source data, extended data, supplementary information, acknowledgements, peer review information; details of author contributions and competing interests; and statements of data and code availability are available at 10.1038/s41586-023-06758-2.

### Supplementary information

Supplementary Information This file contains the Supplementary Discussion and Supplementary Tables 1 and 2.

Reporting Summary

Supplementary Video 1 Response of PRLH neurons to i.g. infusion of Ensure across a 30-min trial. Related to Fig. 1.

Supplementary Video 2 Response of PRLH neurons to the first bout of oral consumption of Ensure. Related to Fig. 1.

Supplementary Video 3 Response of PRLH neurons to oral consumption of Ensure after the first bout. Related to Fig. 2.

Supplementary Video 4 Microendoscopy imaging of individual PRLH neurons in a freely moving mouse. Related to Fig. 3.

Supplementary Video 5 Microendoscopy imaging of individual PRLH neurons in a head-fixed mouse. Related to Fig. 3.

Supplementary Video 6 Microendoscopy imaging of individual PRLH neurons in a head-fixed and restrained mouse. Related to Fig. 3.

Supplementary Video 7 Response of individual PRLH neurons to the first bout of oral consumption of Ensure. Related to Fig. 3.

Supplementary Video 8 Response of GCG neurons to the first bout of oral consumption of Ensure. Related to Fig. 5.

Supplementary Video 9 Response of GCG neurons to oral consumption of Ensure after the first bout. Related to Fig. 5.

## Data Availability

The data from this study are available from the corresponding author on reasonable request.

## Code availability

Links to the code used for data analyses are provided in the Methods.

## References

[1] Berthoud, H.-R. in *Neurobiology of Food and Fluid Intake* (eds Stricker, E. M. & Woods, S. C.) 195–240 (Springer, 2004).

[2] Chambers AP, Sandoval DA, Seeley RJ (2013). Integration of satiety signals by the central nervous system. Curr. Biol..

[3] Cheng W (2022). Hindbrain circuits in the control of eating behaviour and energy balance. Nat. Metab..

[4] Alcantara IC, Tapia APM, Aponte Y, Krashes MJ (2022). Acts of appetite: neural circuits governing the appetitive, consummatory, and terminating phases of feeding. Nat. Metab..

[5] Vrang N, Phifer CB, Corkern MM, Berthoud H-R (2003). Gastric distension induces c-Fos in medullary GLP-1/2-containing neurons. Am. J. Physiol. Regul. Integr. Comp. Physiol..

[6] Williams EK (2016). Sensory neurons that detect stretch and nutrients in the digestive system. Cell.

[7] Ran C, Boettcher JC, Kaye JA, Gallori CE, Liberles SD (2022). A brainstem map for visceral sensations. Nature.

[8] Zittel TT, De Giorgio R, Sternini C, Raybould HE (1994). Fos protein expression in the nucleus of the solitary tract in response to intestinal nutrients in awake rats. Brain Res..

[9] Schwartz GJ, Moran TH (1994). CCK elicits and modulates vagal afferent activity arising from gastric and duodenal sites. Ann. NY Acad. Sci..

[10] Holt MK (2019). Preproglucagon neurons in the nucleus of the solitary tract are the main source of brain GLP-1, mediate stress-induced hypophagia, and limit unusually large intakes of food. Diabetes.

[11] Brierley DI (2021). Central and peripheral GLP-1 systems independently suppress eating. Nat. Metab..

[12] D’Agostino G (2016). Appetite controlled by a cholecystokinin nucleus of the solitary tract to hypothalamus neurocircuit. eLife.

[13] Cheng W (2020). Leptin receptor-expressing nucleus tractus solitarius neurons suppress food intake independently of GLP1 in mice. JCI Insight.

[14] Cheng W (2020). Calcitonin receptor neurons in the mouse nucleus tractus solitarius control energy balance via the non-aversive suppression of feeding. Cell Metab..

[15] Cheng W (2021). NTS Prlh overcomes orexigenic stimuli and ameliorates dietary and genetic forms of obesity. Nat. Commun..

[16] Liu J (2017). Enhanced AMPA receptor trafficking mediates the anorexigenic effect of endogenous glucagon-like peptide-1 in the paraventricular hypothalamus. Neuron.

[17] Roman CW, Derkach VA, Palmiter RD (2016). Genetically and functionally defined NTS to PBN brain circuits mediating anorexia. Nat. Commun..

[18] Schwartz GJ, Moran TH (2002). Leptin and neuropeptide Y have opposing modulatory effects on nucleus of the solitary tract neurophysiological responses to gastric loads: implications for the control of food intake. Endocrinology.

[19] Li, M. et al. Gut–brain circuits for fat preference. *Nature*10.1038/s41586-022-05266-z (2022). 10.1038/s41586-022-05266-z PMC9605869 36070796

[20] Appleyard SM (2007). Visceral afferents directly activate catecholamine neurons in the solitary tract nucleus. J. Neurosci..

[21] Ludwig MQ (2021). A genetic map of the mouse dorsal vagal complex and its role in obesity. Nat. Metab..

[22] Kato HK, Chu MW, Isaacson JS, Komiyama T (2012). Dynamic sensory representations in the olfactory bulb: modulation by wakefulness and experience. Neuron.

[23] Kreisler AD, Davis EA, Rinaman L (2014). Differential activation of chemically identified neurons in the caudal nucleus of the solitary tract in non-entrained rats after intake of satiating vs. non-satiating meals. Physiol. Behav..

[24] Tolhurst G, Reimann F, Gribble FM (2012). Intestinal sensing of nutrients. Handb. Exp. Pharmacol..

[25] Beutler LR (2017). Dynamics of gut–brain communication underlying hunger. Neuron.

[26] Bai L (2022). Enteroendocrine cell types that drive food reward and aversion. eLife.

[27] Lawrence CB, Ellacott KLJ, Luckman SM (2002). PRL-releasing peptide reduces food intake and may mediate satiety signaling. Endocrinology.

[28] Takayanagi Y (2008). Endogenous prolactin-releasing peptide regulates food intake in rodents. J. Clin. Invest..

[29] Chang RS, Lotti VJ (1986). Biochemical and pharmacological characterization of an extremely potent and selective nonpeptide cholecystokinin antagonist. Proc. Natl Acad. Sci. USA.

[30] Laugerette F (2005). CD36 involvement in orosensory detection of dietary lipids, spontaneous fat preference, and digestive secretions. J. Clin. Invest..

[31] Berridge KC, Robinson TE (2016). Liking, wanting and the incentive-sensitization theory of addiction. Am. Psychol..

[32] Gaykema RP (2017). Activation of murine pre-proglucagon-producing neurons reduces food intake and body weight. J. Clin. Invest..

[33] Kreisler AD, Rinaman L (2016). Hindbrain glucagon-like peptide-1 neurons track intake volume and contribute to injection stress-induced hypophagia in meal-entrained rats. Am. J. Physiol. Regul. Integr. Comp. Physiol..

[34] Bai L (2019). Genetic identification of vagal sensory neurons that control feeding. Cell.

[35] Chen Y, Lin Y-C, Zimmerman CA, Essner RA, Knight ZA (2016). Hunger neurons drive feeding through a sustained, positive reinforcement signal. eLife.

[36] Chen Y (2019). Sustained NPY signaling enables AgRP neurons to drive feeding. eLife.

[37] National Institute of Diabetes and Digestive and Kidney Diseases. Dumping syndrome, https://www.niddk.nih.gov/health-information/digestive-diseases/dumping-syndrome (2023).

[38] Wilding JPH (2021). Once-weekly semaglutide in adults with overweight or obesity. N. Engl. J. Med..

[39] Hayashi Y (2009). Mice deficient for glucagon gene-derived peptides display normoglycemia and hyperplasia of islet α-cells but not of intestinal L-cells. Mol. Endocrinol..

[40] Ekstrand MI (2014). Molecular profiling of neurons based on connectivity. Cell.

[41] Smith GP (2001). Sham feeding in rats with chronic, reversible gastric fistulas. Curr. Protoc. Neurosci..

[42] Davis JD, Campbell CS (1973). Peripheral control of meal size in the rat. Effect of sham feeding on meal size and drinking rate. J. Comp. Physiol. Psychol..

