# Supplementary material for: Sequential appetite suppression by oral and visceral feedback to the brainstem
Source: Nature. 2023 Nov 22;624(7990):130–7. doi: 10.1038/s41586-023-06758-2 (PMC10700140; doi:10.1038/s41586-023-06758-2)
Supplement: Supplementary file 1 — This file contains the Supplementary Discussion and Supplementary Tables 1 and 2. [file 41586_2023_6758_MOESM1_ESM.pdf]

nature portfolio

https://doi.org/10.1038/s41586-023-06758-2

Supplementary information

Sequential appetite suppression by oral and visceral feedback to the brainstem

In the format provided by the authors and unedited

Supplementary Information

Supplementary Discussion

PRLH and GCG neurons are distinct cell types in the cNTS that regulate the non-aversive suppression of feeding

In this study, we focused on investigating the two principal cNTS cell types that have been implicated in "non-aversive satiety", i.e. the suppression of feeding in the absence of nausea or sickness[1–10]. One of these cell types is defined by the expression of prolactin-releasing hormone (encoded by the *Prlh* gene) and the other by the expression of glucagon-like peptide 1 (encoded by the *Gcg* gene). These two marker genes are unique in that they label transcriptionally homogeneous cNTS cell types[11] that are intermingled but non-overlapping[11,12], directly innervated by vagal afferents[2,5,13,14], activated by ingestion as measured by *Fos* expression[1,4,15], and functionally validated to inhibit feeding without inducing aversion[2,6,10,16] (Extended Data Fig. 1). These two genes also overlap with several broader, and more heterogeneous, populations of cNTS neurons that have been independently implicated in the control of feeding (described in Extended Data Fig. 1 and refs.[4,6,11,17]).

Post-Ingestive learning does not rescue the response of PRLH neurons to sweet taste

To test whether post-ingestive learning can rescue the rapid activation of PRLH neurons by sweet taste, we gave $Prlh^{Cre}$ $Trpm5^{-/-}$ mice access to a glucose solution (24%) overnight twice, with a day of separation in between (Methods). Long-term exposure to glucose increased glucose consumption in a subset of taste-blind mice, consistent with previous reports that these animals acquire preferences for nutritive solutions via post-ingestive feedback[18–21] (Extended Data Fig. 5m). This increase in ingestion rate partially restored the rapid response of PRLH neurons to glucose ingestion (Extended Data Fig. 5n) but did not rescue the neural response per lick, indicating that this requires taste (0.03 ± 0.01 z for learned animals compared to 0.02 ± 0.01 z for naïve animals, $p = 0.5476$; Extended Data Fig. 5o). Thus, the activation of PRLH neurons by sweet substances requires canonical taste signaling.

PRLH neurons receive orosensory feedback

The rapid activation of PRLH neurons during feeding was driven by an interaction between the taste of food and the rate of ingestion. This was unexpected in part because gustatory signals are relayed primarily to the rostral NTS (rNTS)[22], whereas the cNTS is associated with visceral

feedback transmitted by the vagus nerve. However, PRLH neurons receive descending input from several forebrain structures that could contain information about taste and ingestion dynamics, including the central amygdala, paraventricular hypothalamus, and lateral hypothalamus[23]. In addition, PRLH neurons receive a direct projection from the intermediate reticular formation, a structure that contains premotor neurons for licking[6,24]. The function of these centrifugal projections to the cNTS has received limited attention and dissecting these pathways will be an important area for future investigation.

PRLH neurons do not appear to control sensory-specific satiety

The control of feeding behavior by PRLH neurons appears be unrelated to sensory-specific satiety[25], the process by which repeated exposure to a taste within a meal reduces further consumption of that taste, but does not affect consumption of different tastes. This is because PRLH neuron responses do not increase as the meal progresses (Fig. 1-2) and because blocking PRLH neuron activation does not increase total consumption (Fig. 4).

PRLH neuron activation is gated by behavioral state

PRLH neurons receive abundant feedback from the vagus nerve[5,13] and remain activated for tens of minutes after nutrient infusion into the stomach (Fig. 1b). Thus, the fact that PRLH neurons track orosensory cues during normal ingestion (Fig. 2) implies that their activation by visceral feedback is in some way suppressed during normal feeding. Indeed, we found that the sustained activation of PRLH neurons observed after IG infusion was absent during normal feeding, even when the amount and duration of food consumption/infusion were matched (Fig. 1 and Extended Data Fig. 3h-j). Consistently, the hormone CCK was required for PRLH neuron activation during IG infusion of fat (CCK) but became dispensable during oral ingestion of the same nutrient (Fig. 1g-h). How this hierarchy of sensory responses is established is unknown but could involve the filtering of predicted visceral inputs by cNTS interneurons[26,27] to shift learned neural responses forward in time, as is observed in motivational circuits[28,29]. In this regard, the sensory signals generated during a meal occur in a specific sequence (from mouth to stomach to intestines), and they may be interpreted correctly by the brain only when this natural progression is respected[30]. Alternatively, there may be a signal from mouth-to-gut that suppresses certain GI signals during normal ingestion The ability to monitor cNTS dynamics during behavior will enable investigation of these basic questions about how signals from the mouth and gut interact during a meal.

Microendoscopic imaging of cNTS neurons in awake behaving mice

In this study, we developed a preparation for recording the single-cell activity of PRLH neurons during ingestion. We implanted an angled GRIN lens above the cNTS (Fig. 3e) and first attempted to perform imaging in freely moving animals, which was associated with severe brainstem motion (Fig. 3e; Supplementary Video 4). This motion was reduced by head-fixing, but not to the extent that it was feasible to track the dynamics of single cells (Fig. 3e; Supplementary Video 5). This is consistent with previous reports of unsuccessful attempts to perform cNTS imaging in awake animals[26]. However, we noticed that the largest motion artifacts were correlated with lower body movements of the mouse, which could be eliminated by further restraining the torso of the head-fixed animals (Fig. 3e). This head-fixed, restrained preparation enabled stable calcium recordings while awake mice consumed liquid diets (Supplementary Video 6).

PRLH neurons do not control motor circuits directly

In Figure 4, we investigated the mechanism by which PRLH neuron activity decreases the size of individual lick bouts. It seemed unlikely that PRLH neurons control motor circuits directly, because manipulating PRLH neuron activity did not change the inter-lick interval (ILI) during a bout (Extended Data Fig. 7i-l), a parameter which is determined by central motor pattern generators[31–33] and is invariant during normal behavior. In addition, we failed to observe fictive feeding behaviors (e.g. orofacial movements) in response to PRLH neuron stimulation or silencing in the absence of food, as has been observed for circuits that directly control motor outputs[34].

Disentangling the effects of nutrients from GI distension

In Figure 5, the activation of GCG neurons by post-ingestive feedback could be due to signals of GI stretch, nutrient sensing, or both[35–37]. Of note, because calories and food volume are highly correlated[38], and because calories can increase gastric distension by delaying gastric emptying[39], the feeding experiments described in Figure 5 cannot distinguish between these two mechanisms. To test the sufficiency of GI stretch, we infused into the stomach the non-nutritive sugar mannitol, which is not absorbed from the intestines, and therefore induces significant intestinal distension[38]. Mannitol infusion strongly activated of GCG neurons (5.0 ± 1.7 z, $p =$ 0.0002 compared to baseline; Extended Data Fig. 9k), but not PRLH neurons (0.6 ± 0.6 z, $p =$ 0.7082 compared to baseline; Extended Data Fig. 9l), and the magnitude of this activation was at least as large as that caused by equi-osmotic and equi-volemic infusions of glucose and

Ensure (3.2 ± 0.6 z for Ensure infusion, $p = 0.6282$ compared to mannitol infusion; Extended Data Fig. 9k). To present a pure mechanosensory signal to the stomach, we also performed IG infusions of air (1.0 mL), which activated GCG neurons (2.0 ± 0.3 z, $p < 0.0001$ compared to baseline) but not PRLH neurons (-0.1 ± 0.2 z, $p = 0.8353$ compared to baseline; Extended Data Fig. 9q). Together, these data indicate that GI stretch is sufficient to activate GCG neurons but has little effect on PRLH neurons.

GCG neuron stimulation triggers long-lasting satiety

In Figure 6, we found that GCG neuron pre-stimulation in the absence of food caused a striking, long-lasting suppression of the initiation of new feeding bouts (Fig. 6f). The fact that this response was dose-dependent (Fig. 6g and Extended Data Fig. 10g) suggests that GCG neuron activity can be integrated over time in downstream circuits, likely via release of GLP-1[7], to produce satiety that lasts much longer. Of note, this provides a mechanism to link the amount of food consumed to the time interval before initiation of the next meal. A similar signal integration mechanism has been proposed for hunger-promoting AgRP neurons[40], which promote long-lasting hunger via changes in downstream circuits caused by neuropeptide release[41]. It is likely that competition between these and other neuropeptides, which are released in proportion to the duration of food deprivation or ingestion, is an important part of the mechanism that controls the long-lasting transitions between hunger and satiety.

References

1. Holt, M. K. *et al.* Preproglucagon Neurons in the Nucleus of the Solitary Tract Are the Main Source of Brain GLP-1, Mediate Stress-Induced Hypophagia, and Limit Unusually Large Intakes of Food. *Diabetes* **68**, 21–33 (2019).
2. Brierley, D. I. *et al.* Central and peripheral GLP-1 systems independently suppress eating. *Nat. Metab.* **3**, 258–273 (2021).
3. D'Agostino, G. *et al.* Appetite controlled by a cholecystokinin nucleus of the solitary tract to hypothalamus neurocircuit. *eLife* **5**, e12225 (2016).
4. Cheng, W. *et al.* Leptin receptor-expressing nucleus tractus solitarius neurons suppress food intake independently of GLP1 in mice. *JCI Insight* **5**, 134359 (2020).

5. Cheng, W. *et al.* Calcitonin Receptor Neurons in the Mouse Nucleus Tractus Solitarius Control Energy Balance via the Non-aversive Suppression of Feeding. *Cell Metab.* **31**, 301-312.e5 (2020).
6. Cheng, W. *et al.* NTS Prlh overcomes orexigenic stimuli and ameliorates dietary and genetic forms of obesity. *Nat. Commun.* **12**, 5175 (2021).
7. Liu, J. *et al.* Enhanced AMPA Receptor Trafficking Mediates the Anorexigenic Effect of Endogenous Glucagon-like Peptide-1 in the Paraventricular Hypothalamus. *Neuron* **96**, 897-909.e5 (2017).
8. Roman, C. W., Derkach, V. A. & Palmiter, R. D. Genetically and functionally defined NTS to PBN brain circuits mediating anorexia. *Nat. Commun.* **7**, 11905 (2016).
9. Roman, C. W., Sloat, S. R. & Palmiter, R. D. A tale of two circuits: CCKNTS neuron stimulation controls appetite and induces opposing motivational states by projections to distinct brain regions. *Neuroscience* **358**, 316–324 (2017).
10. Qui, W. *et al.* Multiple NTS Neuron Populations Synergistically Suppress Physiologic Food Intake but are Dispensable for the Response to VSG. 2022.12.23.521804 Preprint at https://doi.org/10.1101/2022.12.23.521804 (2022).
11. Ludwig, M. Q. *et al.* A genetic map of the mouse dorsal vagal complex and its role in obesity. *Nat. Metab.* **3**, 530–545 (2021).
12. Maniscalco, J. W., Kreisler, A. D. & Rinaman, L. Satiation and stress-induced hypophagia: examining the role of hindbrain neurons expressing prolactin-releasing Peptide or glucagon-like Peptide 1. *Front. Neurosci.* **6**, 199 (2012).
13. Appleyard, S. M. *et al.* Visceral afferents directly activate catecholamine neurons in the solitary tract nucleus. *J. Neurosci. Off. J. Soc. Neurosci.* **27**, 13292–13302 (2007).
14. Holt, M. K. *et al.* Synaptic Inputs to the Mouse Dorsal Vagal Complex and Its Resident Preproglucagon Neurons. *J. Neurosci. Off. J. Soc. Neurosci.* **39**, 9767–9781 (2019).

15. Cheng, W. *et al.* NTS Prlh overcomes orexigenic stimuli and ameliorates dietary and genetic forms of obesity. *Nat. Commun.* **12**, 5175 (2021).

16. Gaykema, R. P. *et al.* Activation of murine pre-proglucagon-producing neurons reduces food intake and body weight. *J. Clin. Invest.* **127**, 1031–1045 (2017).

17. Rinaman, L. Hindbrain noradrenergic A2 neurons: diverse roles in autonomic, endocrine, cognitive, and behavioral functions. *Am. J. Physiol. Regul. Integr. Comp. Physiol.* **300**, R222-235 (2011).

18. Zukerman, S., Ackroff, K. & Sclafani, A. Post-oral appetite stimulation by sugars and nonmetabolizable sugar analogs. *Am. J. Physiol. Regul. Integr. Comp. Physiol.* **305**, R840-853 (2013).

19. Sclafani, A., Zukerman, S., Glendinning, J. I. & Margolskee, R. F. Fat and carbohydrate preferences in mice: the contribution of alpha-gustducin and Trpm5 taste-signaling proteins. *Am. J. Physiol. Regul. Integr. Comp. Physiol.* **293**, R1504-1513 (2007).

20. Sclafani, A. & Ackroff, K. Flavor preference conditioning by different sugars in sweet ageusic Trpm5 knockout mice. *Physiol. Behav.* **140**, 156–163 (2015).

21. de Araujo, I. E. *et al.* Food reward in the absence of taste receptor signaling. *Neuron* **57**, 930–941 (2008).

22. Hamilton, R. B. & Norgren, R. Central projections of gustatory nerves in the rat. *J. Comp. Neurol.* **222**, 560–577 (1984).

23. Gasparini, S., Howland, J. M., Thatcher, A. J. & Geerling, J. C. Central afferents to the nucleus of the solitary tract in rats and mice. *J. Comp. Neurol.* **528**, 2708–2728 (2020).

24. Dempsey, B. *et al.* A medullary centre for lapping in mice. *Nat. Commun.* **12**, 6307 (2021).

25. Rolls, B. J., Rolls, E. T., Rowe, E. A. & Sweeney, K. Sensory specific satiety in man. *Physiol. Behav.* **27**, 137–142 (1981).

26. Ran, C., Boettcher, J. C., Kaye, J. A., Gallori, C. E. & Liberles, S. D. A brainstem map for visceral sensations. *Nature* **609**, 320–326 (2022).

27. Bailey, T. W., Appleyard, S. M., Jin, Y.-H. & Andresen, M. C. Organization and properties of GABAergic neurons in solitary tract nucleus (NTS). *J. Neurophysiol.* **99**, 1712–1722 (2008).

28. Ljungberg, T., Apicella, P. & Schultz, W. Responses of monkey dopamine neurons during learning of behavioral reactions. *J. Neurophysiol.* **67**, 145–163 (1992).

29. Cohen, J. Y., Haesler, S., Vong, L., Lowell, B. B. & Uchida, N. Neuron-type-specific signals for reward and punishment in the ventral tegmental area. *Nature* **482**, 85–88 (2012).

30. Antin, J., Gibbs, J. & Smith, G. P. Intestinal satiety requires pregastric food stimulation. *Physiol. Behav.* **18**, 421–425 (1977).

31. Boughter Jr, J. D., Baird, J.-P., Bryant, J., St. John, S. J. & Heck, D. C57BL/6J and DBA/2J mice vary in lick rate and ingestive microstructure. *Genes Brain Behav.* **6**, 619–627 (2007).

32. Travers, J. B., Dinardo, L. A. & Karimnamazi, H. Motor and premotor mechanisms of licking. *Neurosci. Biobehav. Rev.* **21**, 631–647 (1997).

33. Nakamura, Y. & Katakura, N. Generation of masticatory rhythm in the brainstem. *Neurosci. Res.* **23**, 1–19 (1995).

34. Han, W. *et al.* Integrated Control of Predatory Hunting by the Central Nucleus of the Amygdala. *Cell* **168**, 311-324.e18 (2017).

35. Vrang, N., Phifer, C. B., Corkern, M. M. & Berthoud, H.-R. Gastric distension induces c-Fos in medullary GLP-1/2-containing neurons. *Am. J. Physiol. Regul. Integr. Comp. Physiol.* **285**, R470-478 (2003).

36. Tsang, A. H., Nuzzaci, D., Darwish, T., Samudrala, H. & Blouet, C. Nutrient sensing in the nucleus of the solitary tract mediates non-aversive suppression of feeding via inhibition of AgRP neurons. *Mol. Metab.* **42**, 101070 (2020).

37. Tolhurst, G., Reimann, F. & Gribble, F. M. Intestinal sensing of nutrients. *Handb. Exp. Pharmacol.* 309–335 (2012) doi:10.1007/978-3-642-24716-3_14.

38. Bai, L. *et al.* Genetic Identification of Vagal Sensory Neurons That Control Feeding. *Cell* **179**, 1129-1143.e23 (2019).

39. Calbet, J. A. & MacLean, D. A. Role of caloric content on gastric emptying in humans. *J. Physiol.* **498 (Pt 2)**, 553–559 (1997).

40. Chen, Y., Lin, Y.-C., Zimmerman, C. A., Essner, R. A. & Knight, Z. A. Hunger neurons drive feeding through a sustained, positive reinforcement signal. *eLife* **5**, e18640 (2016).

41. Chen, Y. *et al.* Sustained NPY signaling enables AgRP neurons to drive feeding. *eLife* **8**, e46348 (2019).

Supplementary Table 1 | Statistics for photometry and microendoscopy data

Figure	Experiment	Cohort	n	p	test
Fig 1b	Saline IG 1.5 mL 0-30 min	PRLH	6	>0.9999	Šidák's multiple comparisons test
Fig 1b	Ensure IG 1.5 mL 0-30 min	PRLH	3	0.0003	Šidák's multiple comparisons test
Fig 1b	Glucose IG 1.5 mL 0-30 min	PRLH	5	0.0037	Šidák's multiple comparisons test
Fig 1b	MDG IG 1.5 mL 0-30 min	PRLH	6	0.0262	Šidák's multiple comparisons test
Fig 1b	Intralipid IG 1.5 mL 0-30 min	PRLH	6	0.0002	Šidák's multiple comparisons test
Fig 1b	Glucose vs MDG IG 1.5 mL 0-30 min	PRLH	5 and 6	0.3290	Mann-Whitney test
Fig 1c	Water lick 0-10 min	PRLH	9	0.9999	Šidák's multiple comparisons test
Fig 1c	Dry licking 0-10 min	PRLH	11	0.9998	Šidák's multiple comparisons test
Fig 1c	Saline lick 0-10 min	PRLH	6	0.9089	Šidák's multiple comparisons test
Fig 1c	Ensure lick 0-10 min	PRLH	15	<0.0001	Šidák's multiple comparisons test
Fig 1c	Intralipid lick 0-10 min	PRLH	8	0.0002	Šidák's multiple comparisons test
Fig 1c	Glucose lick 0-10 min	PRLH	12	<0.0001	Šidák's multiple comparisons test
Fig 1d	PCC Cumulative licks vs z-score – Ensure lick	PRLH	15	>0.9999	Šidák's multiple comparisons test
Fig 1d	PCC Cumulative licks vs z-score – glucose lick	PRLH	12	0.0093	Šidák's multiple comparisons test
Fig 1d	PCC Cumulative licks vs z-score – Intralipid lick	PRLH	13	0.7953	Šidák's multiple comparisons test

Fig 1d	PCC Cumulative licks vs z-score – saline lick	PRLH	6	0.9518	Šidák's multiple comparisons test
Fig 1d	PCC Cumulative licks vs z-score – dry licks	PRLH	10	0.0043	Šidák's multiple comparisons test
Fig 1d	PCC Cumulative licks vs z-score – water lick	PRLH	11	0.0001	Šidák's multiple comparisons test
Fig 1e	Time to 50% max z-score vs. percent of total glucose oral ingestion	PRLH	12	<0.0001	Šidák's multiple comparisons test
Fig 1e	Time to 50% max z-score vs. percent of total glucose IG infusion	PRLH	5	0.4712	Šidák's multiple comparisons test
Fig 1f	Time to 50% max z-score vs. percent of total Intralipid oral ingestion	PRLH	13	<0.0001	Šidák's multiple comparisons test
Fig 1f	Time to 50% max z-score vs. percent of total Intralipid IG infusion	PRLH	6	0.2946	Šidák's multiple comparisons test
Fig 1g	Intralipid IG	PRLH	6	0.0313	Wilcoxon signed-rank test
Fig 1h	Intralipid lick	PRLH	6	0.3125	Wilcoxon signed-rank test
Fig 2b	PCC Cumulative licks past 10 s vs z-score	PRLH	15	<0.0001	Šidák's multiple comparisons test
Fig 2b	PCC Cumulative licks past 20 s vs z-score	PRLH	15	<0.0001	Šidák's multiple comparisons test
Fig 2b	PCC Cumulative licks past 30 s vs z-score	PRLH	15	<0.0001	Šidák's multiple comparisons test
Fig 2b	PCC Cumulative licks past 1 min vs z-score	PRLH	15	<0.0001	Šidák's multiple comparisons test
Fig 2b	PCC Cumulative licks past 2 min vs z-score	PRLH	15	<0.0001	Šidák's multiple comparisons test
Fig 2b	PCC Cumulative licks past	PRLH	15	<0.0001	Šidák's multiple comparisons test

	3 min vs z-score				
Fig 2b	PCC Cumulative licks past 4 min vs z-score	PRLH	15	<0.0001	Šidák's multiple comparisons test
Fig 2b	PCC Cumulative licks past 5 min vs z-score	PRLH	15	<0.0001	Šidák's multiple comparisons test
Fig 2b	PCC Cumulative licks past 6 min vs z-score	PRLH	15	0.0002	Šidák's multiple comparisons test
Fig 2b	PCC Cumulative licks past 7 min vs z-score	PRLH	15	0.0007	Šidák's multiple comparisons test
Fig 2b	PCC Cumulative licks past 8 min vs z-score	PRLH	15	0.0018	Šidák's multiple comparisons test
Fig 2b	PCC Cumulative licks past 9 min vs z-score	PRLH	15	0.0063	Šidák's multiple comparisons test
Fig 2b	PCC Cumulative licks past 10 min vs z-score	PRLH	15	0.0251	Šidák's multiple comparisons test
Fig 2b	PCC Cumulative licks past 11 min vs z-score	PRLH	15	0.0396	Šidák's multiple comparisons test
Fig 2b	PCC Cumulative licks past 12 min vs z-score	PRLH	15	0.1620	Šidák's multiple comparisons test
Fig 2b	PCC Cumulative licks past 13 min vs z-score	PRLH	15	0.1329	Šidák's multiple comparisons test
Fig 2b	PCC Cumulative licks past 14 min vs z-score	PRLH	15	0.3121	Šidák's multiple comparisons test
Fig 2b	PCC Cumulative licks past 15 min vs z-score	PRLH	15	0.5512	Šidák's multiple comparisons test
Fig 2b	PCC Cumulative licks past 16 min vs z-score	PRLH	15	0.9059	Šidák's multiple comparisons test
Fig 2b	PCC Cumulative licks past 17 min vs z-score	PRLH	15	0.9685	Šidák's multiple comparisons test
Fig 2b	PCC Cumulative licks past	PRLH	15	0.9919	Šidák's multiple comparisons test

	18 min vs z-score				
Fig 2b	PCC Cumulative licks past 19 min vs z-score	PRLH	15	0.9999	Šidák's multiple comparisons test
Fig 2b	PCC Cumulative licks past 20 min vs z-score	PRLH	15	>0.9999	Šidák's multiple comparisons test
Fig 2b	PCC Cumulative licks past 21 min vs z-score	PRLH	15	>0.9999	Šidák's multiple comparisons test
Fig 2b	PCC Cumulative licks past 22 min vs z-score	PRLH	15	>0.9999	Šidák's multiple comparisons test
Fig 2b	PCC Cumulative licks past 23 min vs z-score	PRLH	15	>0.9999	Šidák's multiple comparisons test
Fig 2b	PCC Cumulative licks past 24 min vs z-score	PRLH	15	>0.9999	Šidák's multiple comparisons test
Fig 2b	PCC Cumulative licks past 25 min vs z-score	PRLH	15	>0.9999	Šidák's multiple comparisons test
Fig 2b	PCC Cumulative licks past 26 min vs z-score	PRLH	15	>0.9999	Šidák's multiple comparisons test
Fig 2b	PCC Cumulative licks past 27 min vs z-score	PRLH	15	>0.9999	Šidák's multiple comparisons test
Fig 2b	PCC Cumulative licks past 28 min vs z-score	PRLH	15	>0.9999	Šidák's multiple comparisons test
Fig 2b	PCC Cumulative licks past 29 min vs z-score	PRLH	15	>0.9999	Šidák's multiple comparisons test
Fig 2b	PCC Cumulative licks past 30 min vs z-score	PRLH	15	>0.9999	Šidák's multiple comparisons test
Fig 2f	Water lick first 10s	PRLH	9	0.7869	Šidák's multiple comparisons test
Fig 2f	Dry licking first 10s	PRLH	11	0.6734	Šidák's multiple comparisons test
Fig 2f	Saline lick first 10s	PRLH	6	0.6260	Šidák's multiple comparisons test
Fig 2f	Ensure lick first 10s	PRLH	15	<0.0001	Šidák's multiple comparisons test

Fig 2f	Intralipid lick first 10s	PRLH	8	<0.0001	Šidák's multiple comparisons test
Fig 2f	Glucose lick first 10s	PRLH	12	<0.0001	Šidák's multiple comparisons test
Fig 2g	PCC Ensure lick rate vs z-score	PRLH	15	<0.0001	Šidák's multiple comparisons test
Fig 2g	PCC Glucose lick rate vs z-score	PRLH	12	<0.0001	Šidák's multiple comparisons test
Fig 2g	PCC Intralipid lick rate vs z-score	PRLH	13	<0.0001	Šidák's multiple comparisons test
Fig 2g	PCC Saline lick rate vs z-score	PRLH	6	0.2164	Šidák's multiple comparisons test
Fig 2g	PCC Dry licks lick rate vs z-score	PRLH	10	0.0004	Šidák's multiple comparisons test
Fig 2g	PCC Water lick rate vs z-score	PRLH	11	<0.0001	Šidák's multiple comparisons test
Fig 2h	Water - bout size vs z-score per bout	PRLH	97 bouts (11 mice)	<0.0001	Simple linear regression
Fig 2h	Intralipid - bout size vs z-score per bout	PRLH	164 bouts (13 mice)	<0.0001	Simple linear regression
Fig 2h	Glucose - bout size vs z-score per bout	PRLH	139 bouts (12 mice)	<0.0001	Simple linear regression
Fig 2h	Coefficient x1 water vs glucose	PRLH	11 and 12	0.0108	Dunn's multiple comparison test
Fig 2h	Coefficient x1 water vs intralipid	PRLH	12 and 13	0.0015	Dunn's multiple comparison test
Fig 2i	Water z-score per lick 0-25 licks	PRLH	11	0.0141	Šidák's multiple comparisons test
Fig 2i	Dry licks z-score per lick 0-25 licks	PRLH	10	0.8302	Šidák's multiple comparisons test
Fig 2i	Saline z-score per lick 0-25 licks	PRLH	7	0.4766	Šidák's multiple comparisons test

Fig 2i	Ensure z-score per lick 0-25 licks	PRLH	5	<0.0001	Šidák's multiple comparisons test
Fig 2i	Intralipid z-score per lick 0-25 licks	PRLH	12	<0.0001	Šidák's multiple comparisons test
Fig 2i	Glucose z-score per lick 0-25 licks	PRLH	11	<0.0001	Šidák's multiple comparisons test
Fig 2i	Water z-score per lick 25-50 licks	PRLH	9	>0.9999	Šidák's multiple comparisons test
Fig 2i	Dry licks z-score per lick 25-50 licks	PRLH	9	>0.9999	Šidák's multiple comparisons test
Fig 2i	Saline z-score per lick 25-50 licks	PRLH	6	0.7362	Šidák's multiple comparisons test
Fig 2i	Ensure z-score per lick 25-50 licks	PRLH	9	<0.0001	Šidák's multiple comparisons test
Fig 2i	Intralipid z-score per lick 25-50 licks	PRLH	13	<0.0001	Šidák's multiple comparisons test
Fig 2i	Glucose z-score per lick 25-50 licks	PRLH	11	<0.0001	Šidák's multiple comparisons test
Fig 2i	Water z-score per lick 50-75 licks	PRLH	6	>0.9999	Šidák's multiple comparisons test
Fig 2i	Dry licks z-score per lick 50-75 licks	PRLH	8	>0.9999	Šidák's multiple comparisons test
Fig 2i	Saline z-score per lick 50-75 licks	PRLH	6	0.6277	Šidák's multiple comparisons test
Fig 2i	Ensure z-score per lick 50-75 licks	PRLH	10	0.0087	Šidák's multiple comparisons test
Fig 2i	Intralipid z-score per lick 50-75 licks	PRLH	10	0.0029	Šidák's multiple comparisons test
Fig 2i	Glucose z-score per lick 50-75 licks	PRLH	9	<0.0001	Šidák's multiple comparisons test

Fig 2i	Water z-score per lick 75-100 licks	PRLH	7	>0.9999	Šidák’s multiple comparisons test
Fig 2i	Dry licks z-score per lick 75-100 licks	PRLH	5	>0.9999	Šidák’s multiple comparisons test
Fig 2i	Saline z-score per lick 75-100 licks	PRLH	2	>0.9999	Šidák’s multiple comparisons test
Fig 2i	Ensure z-score per lick 75-100 licks	PRLH	11	0.1083	Šidák’s multiple comparisons test
Fig 2i	Intralipid z-score per lick 75-100 licks	PRLH	8	0.0132	Šidák’s multiple comparisons test
Fig 2i	Glucose z-score per lick 75-100 licks	PRLH	10	0.0060	Šidák’s multiple comparisons test
Fig 3b	CCK IP	PRLH WT and TRPM5 KO	17 and 6	0.2273	Mann-Whitney test
Fig 3c	Glucose lick WT vs KO first 10s	PRLH WT and TRPM5 KO	12 and 6	0.0004	Mann-Whitney test
Fig 3d	Sucralose lick WT vs KO first 10s	PRLH WT and TRPM5 KO	6 and 6	0.0043	Mann-Whitney test
Fig 3j	Pop-weighted z water	PRLH	4	0.5672	Šidák’s multiple comparisons test
Fig 3j	Pop-weighted z Ensure	PRLH	5	<0.0001	Šidák’s multiple comparisons test
Fig 3j	Pop-weighted z Intralipid	PRLH	3	0.0042	Šidák’s multiple comparisons test
Fig 3j	Pop-weighted z sucralose	PRLH	5	0.0003	Šidák’s multiple comparisons test
Fig 3j	Pop-weighted z Ensure vs water	PRLH	5 and 4	0.0159	Mann-Whitney test

Fig 3m	Sucralose lick z-score vs IP CCK z-score	PRLH	67 neurons (3 mice)	0.5	Simple linear regression
Fig 3m	Percentage of neurons responding to sucralose or CCK	PRLH	67 neurons (3 mice)	0.784	Fisher's exact test
Fig 5a	Ensure first 5s	PRLH	14	<0.0001	Šidák's multiple comparisons test
Fig 5a	Ensure first 5s	GCG	7	0.9794	Šidák's multiple comparisons test
Fig 5a	Intralipid first 5s	PRLH	13	<0.0001	Šidák's multiple comparisons test
Fig 5a	Intralipid first 5s	GCG	6	0.6888	Šidák's multiple comparisons test
Fig 5a	Glucose first 5s	PRLH	12	<0.0001	Šidák's multiple comparisons test
Fig 5a	Glucose first 5s	GCG	6	>0.9999	Šidák's multiple comparisons test
Fig 5b	Water lick 0-30 min	GCG	5	0.9947	Šidák's multiple comparisons test
Fig 5b	Dry licking 0-30 min	GCG	8	0.9985	Šidák's multiple comparisons test
Fig 5b	Saline lick 0-30 min	GCG	4	>0.9999	Šidák's multiple comparisons test
Fig 5b	Sucralose lick 0-30 min	GCG	5	0.3867	Šidák's multiple comparisons test
Fig 5b	Ensure lick 0-30 min	GCG	7	<0.0001	Šidák's multiple comparisons test
Fig 5b	Intralipid lick 0-30 min	GCG	6	<0.0001	Šidák's multiple comparisons test
Fig 5b	Glucose lick 0-30 min	GCG	6	0.0075	Šidák's multiple comparisons test
Fig 5c	Ensure brief access 5s z-score vs trial #	GCG	50 (5 mice)	0.98	Simple linear regression
Fig 5d	Ensure brief access 60s z-score vs trial #	GCG	50 (5 mice)	<0.0001	Simple linear regression
Fig 5e	Chow and HFD 10 min access - kcal	GCG	11	<0.0001	Simple linear regression
Fig 5f	Chow and HFD 10 min access - kcal	PRLH	12	0.2314	Simple linear regression
Ex. Data	PCC IG infusion vs z-score	PRLH	3	<0.0001	Šidák's multiple comparisons test

Fig 3d	- Ensure				
Ex. Data Fig 3d	PCC IG infusion vs z-score - Intralipid	PRLH	6	<0.0001	Šidák's multiple comparisons test
Ex. Data Fig 3d	PCC IG infusion vs z-score - Glucose	PRLH	5	<0.0001	Šidák's multiple comparisons test
Ex. Data Fig 3d	PCC IG infusion vs z-score - MDG	PRLH	6	<0.0001	Šidák's multiple comparisons test
Ex. Data Fig 3g	Water lick 0-30 min	PRLH	9	0.9998	Šidák's multiple comparisons test
Ex. Data Fig 3g	Dry licking 0-30 min	PRLH	11	>0.9999	Šidák's multiple comparisons test
Ex. Data Fig 3g	Saline lick 0-30 min	PRLH	6	0.9641	Šidák's multiple comparisons test
Ex. Data Fig 3g	Ensure lick 0-30 min	PRLH	15	<0.0001	Šidák's multiple comparisons test
Ex. Data Fig 3g	Intralipid lick 0-30 min	PRLH	8	0.0005	Šidák's multiple comparisons test
Ex. Data Fig 3g	Glucose lick 0-30 min	PRLH	12	0.0080	Šidák's multiple comparisons test
Ex. Data Fig 3i	Intralipid Oral vs IG during ingestion	PRLH	7	0.2187	Holm-Šidák multiple comparisons test
Ex. Data Fig 3i	Intralipid Oral vs IG post-ingestion	PRLH	7	0.031	Holm-Šidák multiple comparisons test
Ex. Data Fig 3j	Time to 50% - max z-score vs. percent of total Intralipid oral ingestion	PRLH	7	0.0187	Holm-Šidák multiple comparisons test
Ex. Data Fig 3j	Time to 50% - max z-score vs. percent of total Intralipid IG infusion	PRLH	7	0.1905	Holm-Šidák multiple comparisons test

Ex. Data Fig 3l	Empty Cage 10 min	PRLH	11	0.8601	Šidák's multiple comparisons test
Ex. Data Fig 3l	Chow 10 min	PRLH	7	<0.0001	Šidák's multiple comparisons test
Ex. Data Fig 3l	HFD 10 min	PRLH	6	<0.0001	Šidák's multiple comparisons test
Ex. Data Fig 3l	Empty Cage 30 min	PRLH	11	0.5668	Šidák's multiple comparisons test
Ex. Data Fig 3l	Chow 30 min	PRLH	7	<0.0001	Šidák's multiple comparisons test
Ex. Data Fig 3l	HFD 30 min	PRLH	6	<0.0001	Šidák's multiple comparisons test
Ex. Data Fig 3m	Time to 50% Max z-score vs. total bites Chow	PRLH	6	0.0005	Šidák's multiple comparisons test
Ex. Data Fig 3m	Time to 50% Max z-score vs. total bites HFD	PRLH	5	0.0002	Šidák's multiple comparisons test
Ex. Data Fig 3o	PCC Cumulative bites vs z-score - chow	PRLH	5	0.1757	Šidák's multiple comparisons test
Ex. Data Fig 3o	PCC Cumulative bites vs z-score - HFD	PRLH	5	0.1587	Šidák's multiple comparisons test
Ex. Data Fig 3r	CCK IP	PRLH	11	<0.0001	Šidák's multiple comparisons test
Ex. Data Fig 3r	5-HT IP	PRLH	6	0.3999	Šidák's multiple comparisons test
Ex. Data Fig 3r	Amylin IP	PRLH	5	0.9951	Šidák's multiple comparisons test
Ex. Data Fig 3r	Exendin-4 IP	PRLH	6	0.9881	Šidák's multiple comparisons test
Ex. Data	PYY IP	PRLH	5	>0.9999	Šidák's multiple comparisons test

Fig 3r					
Ex. Data Fig 3r	Calcitonin IP	PRLH	6	0.9989	Šidák's multiple comparisons test
Ex. Data Fig 3r	Ghrelin IP	PRLH	5	0.9990	Šidák's multiple comparisons test
Ex. Data Fig 3r	Saline IP	PRLH	11	>0.9999	Šidák's multiple comparisons test
Ex. Data Fig 3s	Intralipid intake	PRLH	6	>0.9999	Wilcoxon signed-rank test
Ex. Data Fig 3t	Tail suspension	PRLH	6	0.4375	Wilcoxon signed-rank test
Ex. Data Fig 3u	Mouse intruder	PRLH	5	0.0625	Wilcoxon signed-rank test
Ex. Data Fig 3v	LiCl IP	PRLH	6	0.7566	Šidák's multiple comparisons test
Ex. Data Fig 3v	LPS IP	PRLH	6	0.8007	Šidák's multiple comparisons test
Ex. Data Fig 4a	Ensure lick first 10s – fasted vs fed	PRLH	15 and 10	0.6433	Mann-Whitney test
Ex. Data Fig 4b	Ensure lick first 10s – early vs late	PRLH	15	0.0676	Wilcoxon signed-rank test
Ex. Data Fig 4c	Ensure z-score per lick early vs late 0-25 licks	PRLH	4 and 1	0.0491	Šidák's multiple comparisons test
Ex. Data Fig 4c	Ensure z-score per lick early vs late 25-50 licks	PRLH	7 and 3	0.9988	Šidák's multiple comparisons test
Ex. Data Fig 4c	Ensure z-score per lick early vs late 50-75 licks	PRLH	5 and 5	>0.9999	Šidák's multiple comparisons test
Ex. Data	Ensure z-score per lick	PRLH	7 and 7	0.9998	Šidák's multiple comparisons test

Fig 4c	early vs late<br>75-100 licks				
Ex. Data<br>Fig 4c	Ensure z-score per lick<br>fasted vs fed<br>0-25 licks	PRLH	5 and 3	0.0798	Šidák's multiple comparisons test
Ex. Data<br>Fig 4c	Ensure z-score per lick<br>fasted vs fed<br>25-50 licks	PRLH	9 and 7	0.9970	Šidák's multiple comparisons test
Ex. Data<br>Fig 4c	Ensure z-score per lick<br>fasted vs fed<br>50-75 licks	PRLH	10 and 8	0.9997	Šidák's multiple comparisons test
Ex. Data<br>Fig 4c	Ensure z-score per lick<br>fasted vs fed<br>75-100 licks	PRLH	11 and 7	>0.9999	Šidák's multiple comparisons test
Ex. Data<br>Fig 4d	Intralipid z-score per lick<br>Early vs late<br>0-25 licks	PRLH	12 and 12	0.9951	Šidák's multiple comparisons test
Ex. Data<br>Fig 4d	Intralipid z-score per lick<br>Early vs late<br>25-50 licks	PRLH	12 and 9	0.7835	Šidák's multiple comparisons test
Ex. Data<br>Fig 4d	Intralipid z-score per lick<br>Early vs late<br>50-75 licks	PRLH	9 and 6	0.9949	Šidák's multiple comparisons test
Ex. Data<br>Fig 4d	Intralipid z-score per lick<br>Early vs late<br>75-100 licks	PRLH	3 and 6	>0.9999	Šidák's multiple comparisons test
Ex. Data<br>Fig 4d	Glucose z-score per lick<br>Early vs late<br>0-25 licks	PRLH	6 and 7	0.9986	Šidák's multiple comparisons test
Ex. Data<br>Fig 4d	Glucose z-score per lick<br>Early vs late<br>25-50 licks	PRLH	10 and 7	0.9939	Šidák's multiple comparisons test
Ex. Data	Glucose z-score per lick	PRLH	9 and 8	0.9998	Šidák's multiple comparisons test

Fig 4d	Early vs late<br>50-75 licks				
Ex. Data Fig 4d	Glucose z-score per lick<br>Early vs late<br>75-100 licks	PRLH	7 and 6	0.8010	Šidák's multiple comparisons test
Ex. Data Fig 4e	Saline z-score per lick<br>Early vs late<br>0-25 licks	PRLH	5 and 5	0.8050	Šidák's multiple comparisons test
Ex. Data Fig 4e	Saline z-score per lick<br>Early vs late<br>25-50 licks	PRLH	5 and 1	0.9116	Šidák's multiple comparisons test
Ex. Data Fig 4e	Saline z-score per lick<br>Early vs late<br>50-75 licks	PRLH	6 and 1	0.8827	Šidák's multiple comparisons test
Ex. Data Fig 4e	Dry licks z-score per lick<br>Early vs late<br>0-25 licks	PRLH	9 and 6	0.9429	Šidák's multiple comparisons test
Ex. Data Fig 4e	Dry licks z-score per lick<br>z-score per lick<br>Early vs late<br>25-50 licks	PRLH	6 and 5	>0.9999	Šidák's multiple comparisons test
Ex. Data Fig 4e	Dry licks z-score per lick<br>z-score per lick<br>Early vs late<br>50-75 licks	PRLH	6 and 3	0.9994	Šidák's multiple comparisons test
Ex. Data Fig 4e	Dry licks z-score per lick<br>z-score per lick<br>Early vs late<br>75-100 licks	PRLH	4 and 3	0.8573	Šidák's multiple comparisons test
Ex. Data Fig 4e	Water z-score per lick<br>Early vs late<br>0-25 licks	PRLH	11 and 5	0.9911	Šidák's multiple comparisons test
Ex. Data	Water z-score per lick	PRLH	8 and 4	0.9669	Šidák's multiple comparisons test

Fig 4e	z-score per lick<br>Early vs late<br>25-50 licks				
Ex. Data<br>Fig 4e	Water z-score per lick<br>z-score per lick<br>Early vs late<br>50-75 licks	PRLH	5 and 2	0.9811	Šidák's multiple comparisons test
Ex. Data<br>Fig 4f	Esophageal distension<br>0-30 s	PRLH	7	0.3750	Wilcoxon signed-rank test
Ex. Data<br>Fig 4j	Saline - bout size vs z-score per bout	PRLH	42 bouts (7 mice)	<0.0001	Simple linear regression
Ex. Data<br>Fig 4j	Dry licks - bout size vs z-score per bout	PRLH	82 bouts (10 mice)	<0.0001	Simple linear regression
Ex. Data<br>Fig 5b	Sucralose vs glucose lick - first 10s	PRLH	12 and 6	0.4371	Mann-Whitney Test
Ex. Data<br>Fig 5c	Sucralose vs glucose<br>z-score per lick<br>Main effect of tastant	PRLH	F (1, 53) = 0.06248	0.8036	Two-way ANOVA
Ex. Data<br>Fig 5c	Sucralose vs glucose<br>z-score per lick<br>0-25 licks	PRLH	4 and 10	0.1209	Šidák's multiple comparisons test
Ex. Data<br>Fig 5c	Sucralose vs glucose<br>z-score per lick<br>25-50 licks	PRLH	6 and 11	0.9294	Šidák's multiple comparisons test
Ex. Data<br>Fig 5c	Sucralose vs glucose<br>z-score per lick<br>50-75 licks	PRLH	4 and 11	0.5578	Šidák's multiple comparisons test
Ex. Data<br>Fig 5c	Sucralose vs glucose<br>z-score per lick<br>75-100 licks	PRLH	6 and 9	0.9992	Šidák's multiple comparisons test
Ex. Data<br>Fig 5d	Sucralose vs glucose<br>0-10 min	PRLH	6 and 12	0.1246	Mann-Whitney test

Ex. Data Fig 5d	Sucralose vs glucose 0-30 min	PRLH	6 and 12	0.8916	Mann-Whitney test
Ex. Data Fig 5e	Sucralose - bout size vs z-score per bout	PRLH	96 bouts (6 mice)	<0.0001	Simple linear regression
Ex. Data Fig 5f	Mean PCC Sucralose vs glucose	PRLH	12 and 6	0.2496	Mann Whitney test
Ex. Data Fig 5h	Sucralose IG 1.5 mL 0-30 min	PRLH	5	0.6250	Wilcoxon signed-rank test
Ex. Data Fig 5k	Glucose WT vs KO z-score per lick Main effect of genotype	PRLH WT and TRPM5 KO	F (1, 45) = 21.13	<0.0001	Two-way ANOVA
Ex. Data Fig 5k	Glucose WT vs KO z-score per lick 0-25 licks	PRLH WT and TRPM5 KO	4 and 10	0.0012	Šidák's multiple comparisons test
Ex. Data Fig 5k	Glucose WT vs KO z-score per lick 25-50 licks	PRLH WT and TRPM5 KO	4 and 11	0.1090	Šidák's multiple comparisons test
Ex. Data Fig 5k	Glucose WT vs KO z-score per lick 50-75 licks	PRLH WT and TRPM5 KO	2 and 11	0.2863	Šidák's multiple comparisons test
Ex. Data Fig 5k	Glucose WT vs KO z-score per lick 75-100 licks	PRLH WT and TRPM5 KO	2 and 9	0.3459	Šidák's multiple comparisons test
Ex. Data Fig 5l	Glucose - bout size vs z-score per bout KO	PRLH TRPM5 KO	32	<0.0001	Simple linear regression
Ex. Data Fig 5m	Cumulative glucose licks – KO (naïve) vs KO (learned)	PRLH TRPM5 KO	6 and 3	0.0238	Mann-Whitney test
Ex. Data Fig 5n	Glucose lick KO (naïve) vs KO (learned) first 10s	PRLH TRPM5 KO	6 and 3	0.0476	Mann-Whitney test
Ex. Data	Glucose z-score per lick	PRLH	6 and 3	0.5476	Mann-Whitney test

Fig 5o	KO (naïve) vs KO (learned)	TRPM5 KO			
Ex. Data Fig 5q	Sucralose WT vs KO z-score per lick Main effect of genotype	PRLH WT and TRPM5 KO	F (1, 26) = 12.20	0.0017	Two-way ANOVA
Ex. Data Fig 5q	Sucralose WT vs KO z-score per lick 0-25 licks	PRLH WT and TRPM5 KO	4 and 4	0.0005	Šidák's multiple comparisons test
Ex. Data Fig 5q	Sucralose WT vs KO z-score per lick 25-50 licks	PRLH WT and TRPM5 KO	6 and 5	0.4672	Šidák's multiple comparisons test
Ex. Data Fig 5q	Sucralose WT vs KO z-score per lick 50-75 licks	PRLH WT and TRPM5 KO	4 and 3	0.9909	Šidák's multiple comparisons test
Ex. Data Fig 5q	Sucralose WT vs KO z-score per lick 75-100 licks	PRLH WT and TRPM5 KO	6 and 2	0.8848	Šidák's multiple comparisons test
Ex. Data Fig 5r	Sucralose - bout size vs z-score per bout KO	PRLH TRPM5 KO	30	<0.0001	Simple linear regression
Ex. Data Fig 5t	Intralipid lick WT vs KO first 10s	PRLH WT and TRPM5 KO	8 and 5	0.2222	Mann-Whitney test
Ex. Data Fig 5u	Intralipid WT vs KO z-score per lick Main effect of genotype	PRLH WT and TRPM5 KO	F (1, 47) = 4.329	0.0429	Two-way ANOVA
Ex. Data Fig 5u	Intralipid WT vs KO z-score per lick 0-25 licks	PRLH WT and TRPM5 KO	12 and 2	0.9419	Šidák's multiple comparisons test
Ex. Data Fig 5u	Intralipid WT vs KO z-score per lick 25-50 licks	PRLH WT and TRPM5 KO	13 and 2	0.7781	Šidák's multiple comparisons test
Ex. Data Fig 5u	Intralipid WT vs KO z-score per lick	PRLH WT and TRPM5	10 and 4	0.4554	Šidák's multiple comparisons test

	50-75 licks	KO			
Ex. Data Fig 5u	Intralipid WT vs KO z-score per lick 75-100 licks	PRLH WT and TRPM5 KO	8 and 4	0.7100	Šidák's multiple comparisons test
Ex. Data Fig 5v	Intralipid - bout size vs z-score per bout KO	PRLH TRPM5 KO	32	<0.0001	Simple linear regression
Ex. Data Fig 5w	Coefficient x1 WT vs KO Main effect of genotype	PRLH WT vs TRPM5 KO	F(1,42) = 18.22	0.0001	Two-way ANOVA
Ex. Data Fig 5w	Coefficient x1 WT vs KO Glucose	PRLH WT vs TRPM5 KO	12 and 6	0.2197	Šidák's multiple comparisons test
Ex. Data Fig 5w	Coefficient x1 WT vs KO Sucralose	PRLH WT vs TRPM5 KO	6 and 6	0.0062	Šidák's multiple comparisons test
Ex. Data Fig 5w	Coefficient x1 WT vs KO Intralipid	PRLH WT vs TRPM5 KO	13 and 5	0.0943	Šidák's multiple comparisons test
Ex. Data Fig 6e	Pop-weighted z Ensure all bouts	PRLH	KW stat = 6.726	0.0812	Kruskal-Wallis test
Ex. Data Fig 6i	Pop-weighted z Intralipid all bouts	PRLH	KW stat = 1.051	0.8395	Kruskal-Wallis test
Ex. Data Fig 6m	Pop-weighted z Sucralose all bouts	PRLH	KW stat = 1.537	0.6737	Kruskal-Wallis test
Ex. Data Fig 6q	Pop-weighted z Water all bouts	PRLH	KW stat = 0.9926	0.8309	Kruskal-Wallis test
Ex. Data Fig 8e	Ensure lick PRLH vs GCG – first 10s	PRLH and GCG	15 and 7	0.0029	Mann-Whitney test
Ex. Data Fig 8f	Ensure lick PRLH vs GCG – 15s after bout	PRLH and GCG	15 and 7	0.0213	Mann-Whitney test
Ex. Data	PCC Ensure lick rate vs z-	PRLH and	15 and 7	0.0137	Mann-Whitney test

Fig 8g	score – PRLH and GCG	GCG			
Ex. Data Fig 8i	Dry licking first 10s	GCG	8	>0.9999	Šidák's multiple comparisons test
Ex. Data Fig 8i	Water lick first 10s	GCG	5	0.9841	Šidák's multiple comparisons test
Ex. Data Fig 8i	Saline lick first 10s	GCG	4	>0.9999	Šidák's multiple comparisons test
Ex. Data Fig 8i	Sucralose lick first 10s	GCG	5	0.4132	Šidák's multiple comparisons test
Ex. Data Fig 8i	Ensure lick first 10s	GCG	7	0.0005	Šidák's multiple comparisons test
Ex. Data Fig 8i	Intralipid lick first 10s	GCG	6	0.0042	Šidák's multiple comparisons test
Ex. Data Fig 8i	Glucose lick first 10s	GCG	6	0.0065	Šidák's multiple comparisons test
Ex. Data Fig 8k	PCC Cumulative licks past 10 s vs z-score	GCG	7	0.3051	Šidák's multiple comparisons test
Ex. Data Fig 8k	PCC Cumulative licks past 20 s vs z-score	GCG	7	0.5777	Šidák's multiple comparisons test
Ex. Data Fig 8k	PCC Cumulative licks past 30 s vs z-score	GCG	7	0.2699	Šidák's multiple comparisons test
Ex. Data Fig 8k	PCC Cumulative licks past 1 min vs z-score	GCG	7	0.2209	Šidák's multiple comparisons test
Ex. Data Fig 8k	PCC Cumulative licks past 2 min vs z-score	GCG	7	0.1756	Šidák's multiple comparisons test
Ex. Data Fig 8k	PCC Cumulative licks past 3 min vs z-score	GCG	7	0.0666	Šidák's multiple comparisons test
Ex. Data Fig 8k	PCC Cumulative licks past 4 min vs z-score	GCG	7	0.0344	Šidák's multiple comparisons test
Ex. Data	PCC Cumulative licks past	GCG	7	0.0247	Šidák's multiple comparisons test

Fig 8k	5 min vs z-score				
Ex. Data Fig 8k	PCC Cumulative licks past 6 min vs z-score	GCG	7	0.0331	Šidák’s multiple comparisons test
Ex. Data Fig 8k	PCC Cumulative licks past 7 min vs z-score	GCG	7	0.0318	Šidák’s multiple comparisons test
Ex. Data Fig 8k	PCC Cumulative licks past 8 min vs z-score	GCG	7	0.0193	Šidák’s multiple comparisons test
Ex. Data Fig 8k	PCC Cumulative licks past 9 min vs z-score	GCG	7	0.0212	Šidák’s multiple comparisons test
Ex. Data Fig 8k	PCC Cumulative licks past 10 min vs z-score	GCG	7	0.0398	Šidák’s multiple comparisons test
Ex. Data Fig 8k	PCC Cumulative licks past 11 min vs z-score	GCG	7	0.0581	Šidák’s multiple comparisons test
Ex. Data Fig 8k	PCC Cumulative licks past 12 min vs z-score	GCG	7	0.1041	Šidák’s multiple comparisons test
Ex. Data Fig 8k	PCC Cumulative licks past 13 min vs z-score	GCG	7	0.2045	Šidák’s multiple comparisons test
Ex. Data Fig 8k	PCC Cumulative licks past 14 min vs z-score	GCG	7	0.3496	Šidák’s multiple comparisons test
Ex. Data Fig 8k	PCC Cumulative licks past 15 min vs z-score	GCG	7	0.5980	Šidák’s multiple comparisons test
Ex. Data Fig 8k	PCC Cumulative licks past 16 min vs z-score	GCG	7	0.6601	Šidák’s multiple comparisons test
Ex. Data Fig 8k	PCC Cumulative licks past 17 min vs z-score	GCG	7	0.6944	Šidák’s multiple comparisons test
Ex. Data Fig 8k	PCC Cumulative licks past 18 min vs z-score	GCG	7	0.9107	Šidák’s multiple comparisons test
Ex. Data Fig 8k	PCC Cumulative licks past 19 min vs z-score	GCG	7	0.9582	Šidák’s multiple comparisons test
Ex. Data	PCC Cumulative licks past	GCG	7	0.9765	Šidák’s multiple comparisons test

Fig 8k	20 min vs z-score				
Ex. Data Fig 8k	PCC Cumulative licks past 21 min vs z-score	GCG	7	>0.9999	Šidák's multiple comparisons test
Ex. Data Fig 8k	PCC Cumulative licks past 22 min vs z-score	GCG	7	>0.9999	Šidák's multiple comparisons test
Ex. Data Fig 8k	PCC Cumulative licks past 23 min vs z-score	GCG	7	>0.9999	Šidák's multiple comparisons test
Ex. Data Fig 8k	PCC Cumulative licks past 24 min vs z-score	GCG	7	>0.9999	Šidák's multiple comparisons test
Ex. Data Fig 8k	PCC Cumulative licks past 25 min vs z-score	GCG	7	>0.9999	Šidák's multiple comparisons test
Ex. Data Fig 8k	PCC Cumulative licks past 26 min vs z-score	GCG	7	>0.9999	Šidák's multiple comparisons test
Ex. Data Fig 8k	PCC Cumulative licks past 27 min vs z-score	GCG	7	>0.9999	Šidák's multiple comparisons test
Ex. Data Fig 8k	PCC Cumulative licks past 28 min vs z-score	GCG	7	>0.9999	Šidák's multiple comparisons test
Ex. Data Fig 8k	PCC Cumulative licks past 29 min vs z-score	GCG	7	>0.9999	Šidák's multiple comparisons test
Ex. Data Fig 8k	PCC Cumulative licks past 30 min vs z-score	GCG	7	>0.9999	Šidák's multiple comparisons test
Ex. Data Fig 8l	Water z-score per lick	GCG	5	0.0024	Šidák's multiple comparisons test
Ex. Data Fig 8l	Saline z-score per lick	GCG	4	>0.9999	Šidák's multiple comparisons test
Ex. Data Fig 8l	Dry licks z-score per lick	GCG	8	0.9760	Šidák's multiple comparisons test
Ex. Data Fig 8l	Sucralose z-score per lick	GCG	5	0.9691	Šidák's multiple comparisons test
Ex. Data	Ensure z-score per lick	GCG	7	0.0124	Šidák's multiple comparisons test

Fig 8l					
Ex. Data Fig 8l	Glucose z-score per lick	GCG	6	0.0120	Šidák's multiple comparisons test
Ex. Data Fig 8l	Intralipid z-score per lick	GCG	6	0.1276	Šidák's multiple comparisons test
Ex. Data Fig 8m	Water z-score per lick 0-25 licks	GCG	5 and 5	>0.9999	Šidák's multiple comparisons test
Ex. Data Fig 8m	Dry licks z-score per lick 0-25 licks	GCG	7 and 7	0.9793	Šidák's multiple comparisons test
Ex. Data Fig 8m	Saline z-score per lick 0-25 licks	GCG	4 and 4	>0.9999	Šidák's multiple comparisons test
Ex. Data Fig 8m	Sucralose z-score per lick 0-25 licks	GCG	4 and 4	>0.9999	Šidák's multiple comparisons test
Ex. Data Fig 8m	Ensure z-score per lick 0-25 licks	GCG	4 and 4	>0.9999	Šidák's multiple comparisons test
Ex. Data Fig 8m	Intralipid z-score per lick 0-25 licks	GCG	2 and 2	>0.9999	Šidák's multiple comparisons test
Ex. Data Fig 8m	Glucose z-score per lick 0-25 licks	GCG	5 and 5	0.3913	Šidák's multiple comparisons test
Ex. Data Fig 8m	Water z-score per lick 25-50 licks	GCG	5 and 5	>0.9999	Šidák's multiple comparisons test
Ex. Data Fig 8m	Dry licks z-score per lick 25-50 licks	GCG	6 and 6	>0.9999	Šidák's multiple comparisons test
Ex. Data Fig 8m	Saline z-score per lick 25-50 licks	GCG	4 and 4	>0.9999	Šidák's multiple comparisons test
Ex. Data Fig 8m	Sucralose z-score per lick 25-50 licks	GCG	4 and 4	0.9994	Šidák's multiple comparisons test
Ex. Data Fig 8m	Ensure z-score per lick 25-50 licks	GCG	2 and 2	0.9488	Šidák's multiple comparisons test

Ex. Data Fig 8m	Intralipid z-score per lick 25-50 licks	GCG	5 and 5	0.8862	Šidák's multiple comparisons test
Ex. Data Fig 8m	Glucose z-score per lick 25-50 licks	GCG	4 and 4	0.9967	Šidák's multiple comparisons test
Ex. Data Fig 8m	Water z-score per lick 50-75 licks	GCG	2 and 2	>0.9999	Šidák's multiple comparisons test
Ex. Data Fig 8m	Dry licks z-score per lick 50-75 licks	GCG	4 and 4	>0.9999	Šidák's multiple comparisons test
Ex. Data Fig 8m	Saline z-score per lick 50-75 licks	GCG	2 and 2	>0.9999	Šidák's multiple comparisons test
Ex. Data Fig 8m	Sucralose z-score per lick 50-75 licks	GCG	4 and 4	>0.9999	Šidák's multiple comparisons test
Ex. Data Fig 8m	Ensure z-score per lick 50-75 licks	GCG	5 and 5	>0.9999	Šidák's multiple comparisons test
Ex. Data Fig 8m	Intralipid z-score per lick 50-75 licks	GCG	3 and 3	>0.9999	Šidák's multiple comparisons test
Ex. Data Fig 8m	Glucose z-score per lick 50-75 licks	GCG	3 and 3	0.9916	Šidák's multiple comparisons test
Ex. Data Fig 8m	Water z-score per lick 75-100 licks	GCG	1 and 1	>0.9999	Šidák's multiple comparisons test
Ex. Data Fig 8m	Dry licks z-score per lick 75-100 licks	GCG	5 and 5	>0.9999	Šidák's multiple comparisons test
Ex. Data Fig 8m	Saline z-score per lick 75-100 licks	GCG	1 and 1	>0.9999	Šidák's multiple comparisons test
Ex. Data Fig 8m	Sucralose z-score per lick 75-100 licks	GCG	4 and 4	>0.9999	Šidák's multiple comparisons test
Ex. Data Fig 8m	Ensure z-score per lick 75-100 licks	GCG	7 and 7	0.9983	Šidák's multiple comparisons test
Ex. Data	Intralipid z-score per lick	GCG	4 and 4	>0.9999	Šidák's multiple comparisons test

Fig 8m	75-100 licks				
Ex. Data Fig 8m	Glucose z-score per lick 75-100 licks	GCG	2 and 2	>0.9999	Šidák's multiple comparisons test
Ex. Data Fig 8n	Glucose WT vs KO z-score per lick Main effect of genotype	GCG WT and TRPM5 KO	F (1, 19) = 0.9139	0.3511	Two-way ANOVA
Ex. Data Fig 8n	Sucralose WT vs KO z-score per lick Main effect of genotype	GCG WT and TRPM5 KO	F (1, 23) = 0.3817	0.5470	Two-way ANOVA
Ex. Data Fig 8n	Intralipid WT vs KO z-score per lick Main effect of genotype	GCG WT and TRPM5 KO	F (1, 18) = 0.037	0.8493	Two-way ANOVA
Ex. Data Fig 8o	Esophageal distension 0-30 s	GCG	6	0.0625	Wilcoxon signed-rank test
Ex. Data Fig 8r	Empty Cage 10 min	GCG	7	0.9988	Šidák's multiple comparisons test
Ex. Data Fig 8r	Chow 10 min	GCG	7	0.0079	Šidák's multiple comparisons test
Ex. Data Fig 8r	HFD 10 min	GCG	7	<0.0001	Šidák's multiple comparisons test
Ex. Data Fig 8r	Empty Cage 30 min	GCG	7	0.9995	Šidák's multiple comparisons test
Ex. Data Fig 8r	Chow 30 min	GCG	7	0.0917	Šidák's multiple comparisons test
Ex. Data Fig 8r	HFD 30 min	GCG	7	0.0002	Šidák's multiple comparisons test
Ex. Data Fig 9a	Tail suspension	GCG	5	>0.9999	Wilcoxon signed-rank test
Ex. Data Fig 9b	Mouse intruder	PRLH	5	0.0625	Wilcoxon signed-rank test
Ex. Data	LiCl IP	GCG	5	0.9351	Šidák's multiple comparisons test

Fig 9c					
Ex. Data Fig 9c	LPS IP	GCG	5	0.3885	Šidák's multiple comparisons test
Ex. Data Fig 9d	5HT IP	GCG	5	0.0225	Šidák's multiple comparisons test
Ex. Data Fig 9d	CCK IP	GCG	6	0.9853	Šidák's multiple comparisons test
Ex. Data Fig 9d	Amylin IP	GCG	4	0.9538	Šidák's multiple comparisons test
Ex. Data Fig 9d	Exendin-4 IP	GCG	5	0.5400	Šidák's multiple comparisons test
Ex. Data Fig 9d	PYY IP	GCG	6	0.8831	Šidák's multiple comparisons test
Ex. Data Fig 9d	Calcitonin IP	GCG	4	>0.9999	Šidák's multiple comparisons test
Ex. Data Fig 9d	Ghrelin IP	GCG	5	0.0955	Šidák's multiple comparisons test
Ex. Data Fig 9d	Saline IP	GCG	8	>0.9999	Šidák's multiple comparisons test
Ex. Data Fig 9e	Ensure brief access 5s z-score	GCG	5	0.7365	Šidák's multiple comparisons test
Ex. Data Fig 9e	Ensure brief access 60s z-score	GCG	5	0.0003	Šidák's multiple comparisons test
Ex. Data Fig 9f	Chow during ingestion	GCG	5	0.3174	Šidák's multiple comparisons test
Ex. Data Fig 9f	HFD during ingestion	GCG	6	0.0007	Šidák's multiple comparisons test
Ex. Data Fig 9f	Chow post-ingestion	GCG	5	0.9995	Šidák's multiple comparisons test
Ex. Data	HFD post-ingestion	GCG	6	0.0132	Šidák's multiple comparisons test

Fig 9f					
Ex. Data Fig 9g	Chow and HFD 10 min access - grams	GCG	11	0.0005	Simple linear regression
Ex. Data Fig 9h	Chow during ingestion	PRLH	6	0.0237	Šidák's multiple comparisons test
Ex. Data Fig 9h	HFD during ingestion	PRLH	6	0.0005	Šidák's multiple comparisons test
Ex. Data Fig 9h	Chow post-ingestion	PRLH	6	0.7238	Šidák's multiple comparisons test
Ex. Data Fig 9h	HFD post-ingestion	PRLH	6	0.6745	Šidák's multiple comparisons test
Ex. Data Fig 9i	Chow and HFD 10 min access - grams	PRLH	12	0.28	Simple linear regression
Ex. Data Fig 9k	Saline IG 1 mL 0-10 min	GCG	5	0.9996	Šidák's multiple comparisons test
Ex. Data Fig 9k	Ensure IG 1 mL 0-10 min	GCG	6	0.0005	Šidák's multiple comparisons test
Ex. Data Fig 9k	Glucose IG 1 mL 0-10 min	GCG	6	0.0012	Šidák's multiple comparisons test
Ex. Data Fig 9k	Mannitol IG 1 mL 0-10 min	GCG	7	<0.0001	Šidák's multiple comparisons test
Ex. Data Fig 9k	Saline IG 1 mL 0-30 min	GCG	5	>0.9999	Šidák's multiple comparisons test
Ex. Data Fig 9k	Ensure IG 1 mL 0-30 min	GCG	6	0.0429	Šidák's multiple comparisons test
Ex. Data Fig 9k	Glucose IG 1 mL 0-30 min	GCG	6	0.0219	Šidák's multiple comparisons test
Ex. Data Fig 9k	Mannitol IG 1 mL 0-30 min	GCG	7	0.0002	Šidák's multiple comparisons test
Ex. Data	Ensure vs Mannitol IG	GCG	7 and 6	0.6282	Mann-Whitney test

Fig 9k	0-30 min				
Ex. Data Fig 9k	Glucose IG 1 mL 0-30 min	GCG and PRLH	5 and 6	0.0173	Mann-Whitney test
Ex. Data Fig 9l	Saline IG 1 mL 0-10 min	PRLH	8	0.7914	Šidák’s multiple comparisons test
Ex. Data Fig 9l	Ensure IG 1 mL 0-10 min	PRLH	6	<0.0001	Šidák’s multiple comparisons test
Ex. Data Fig 9l	Glucose IG 1 mL 0-10 min	PRLH	5	0.1160	Šidák’s multiple comparisons test
Ex. Data Fig 9l	Mannitol IG 1 mL 0-10 min	PRLH	6	0.0304	Šidák’s multiple comparisons test
Ex. Data Fig 9l	Saline IG 1 mL 0-30 min	PRLH	8	0.2897	Šidák’s multiple comparisons test
Ex. Data Fig 9l	Ensure IG 1 mL 0-30 min	PRLH	6	0.0005	Šidák’s multiple comparisons test
Ex. Data Fig 9l	Glucose IG 1 mL 0-30 min	PRLH	5	0.9277	Šidák’s multiple comparisons test
Ex. Data Fig 9l	Mannitol IG 1 mL 0-30 min	PRLH	6	0.7082	Šidák’s multiple comparisons test
Ex. Data Fig 9m	PCC IG infusion vs z-score - Ensure	GCG	6	<0.0001	Šidák’s multiple comparisons test
Ex. Data Fig 9m	PCC IG infusion vs z-score – Glucose	GCG	6	<0.0001	Šidák’s multiple comparisons test
Ex. Data Fig 9m	PCC IG infusion vs z-score - Intralipid	GCG	6	0.0051	Šidák’s multiple comparisons test
Ex. Data Fig 9n	Oral vs IG Glucose During Ingestion	GCG	5	0.6836	Holm-Šidák multiple comparisons test
Ex. Data Fig 9n	Oral vs IG Glucose Post Ingestion	GCG	5	0.8125	Holm-Šidák multiple comparisons test
Ex. Data	Oral vs IG Intralipid	GCG	5	0.1211	Holm-Šidák multiple comparisons test

Fig 9o	During Ingestion				
Ex. Data Fig 9o	Oral vs IG Intralipid Post Ingestion	GCG	5	0.3125	Holm-Šidák multiple comparisons test
Ex. Data Fig 9p	Intralipid IG	GCG TRPM5 KO	5	<0.0001	Šidák's multiple comparisons test
Ex. Data Fig 9p	Saline IG	GCG TRPM5 KO	5	0.9699	Šidák's multiple comparisons test
Ex. Data Fig 9q	IG air 1 mL 0-10 min	GCG	3	<0.0001	Šidák's multiple comparisons test
Ex. Data Fig 9q	IG air 1 mL 0-10 min	PRLH	7	0.8353	Šidák's multiple comparisons test
Ex. Data Fig 9q	IG air 1 mL 0-30 min	GCG	3	0.0165	Šidák's multiple comparisons test
Ex. Data Fig 9q	IG air 1 mL 0-30 min	PRLH	7	0.4037	Šidák's multiple comparisons test
Ex. Data Fig 9r	Intralipid IG	GCG	4	>0.9999	Wilcoxon signed-rank test
Ex. Data Fig 9s	Intralipid lick	GCG	5	0.8125	Wilcoxon signed-rank test
Ex. Data Fig 9t	Intralipid intake	GCG	5	0.8125	Wilcoxon signed-rank test

Statistics are shown for each variable quantified during a given experiment in each figure. The number of mice (n) recorded in the experimental cohort are given alongside the p-value measured by the given statistical test. Cohorts of mice are separated by their cell type or genotype.

Supplementary Table 2 | Statistics for optogenetic data

Figure	Experiment	Cohort	n	p	test
Ex. Data Fig 3a	Continuous Stim FD Chow	PRLH-ChR2	8	0.0011	Šidák's multiple comparisons test
Ex. Data Fig 3a	Continuous Stim FD Chow	PRLH-ChR2 Control	5	0.7463	Šidák's multiple comparisons test
Ex. Data Fig 3b	Continuous Stim Dark Phase Ensure Intake	PRLH-ChR2	7	0.0403	Šidák's multiple comparisons test
Ex. Data Fig 3b	Continuous Stim Dark Phase Ensure Intake	PRLH-ChR2 Control	5	0.8723	Šidák's multiple comparisons test
Ex. Data Fig 3b	Continuous Stim Dark Phase Ensure Bout size	PRLH-ChR2	7	0.0222	Šidák's multiple comparisons test
Ex. Data Fig 3b	Continuous Stim Dark Phase Ensure Bout size	PRLH-ChR2 Control	5	0.8434	Šidák's multiple comparisons test
Ex. Data Fig 3b	Continuous Stim Dark Phase Ensure Bout number	PRLH-ChR2	7	0.0323	Šidák's multiple comparisons test
Ex. Data Fig 3b	Continuous Stim Dark Phase Ensure Bout number	PRLH-ChR2 Control	5	0.7656	Šidák's multiple comparisons test
Ex. Data Fig 3c	Continuous Stim WD Water	PRLH-ChR2	6	0.9336	Šidák's multiple comparisons test
Ex. Data Fig 3c	Continuous Stim WD Water	PRLH-ChR2 Control	5	0.8350	Šidák's multiple comparisons test
Fig 4b;	Closed Loop Stim	PRLH-	6	0.0007	Šidák's multiple comparisons test

Ex. Data Fig 7b	Dark Phase Ensure Intake	ChR2			
Fig 4b; Ex. Data Fig 7b	Closed Loop Stim Dark Phase Ensure Intake	PRLH-ChR2 Control	5	0.9986	Šidák's multiple comparisons test
Fig 4b; Ex. Data Fig 7b	Closed Loop Stim Dark Phase Ensure Bout size	PRLH-ChR2	6	<0.0001	Šidák's multiple comparisons test
Fig 4b; Ex. Data Fig 7b	Closed Loop Stim Dark Phase Ensure Bout size	PRLH-ChR2 Control	5	0.8369	Šidák's multiple comparisons test
Fig 4b; Ex. Data Fig 7b	Closed Loop Stim Dark Phase Ensure Bout number	PRLH-ChR2	6	0.2153	Šidák's multiple comparisons test
Fig 4b; Ex. Data Fig 7b	Closed Loop Stim Dark Phase Ensure Bout number	PRLH-ChR2 Control	5	0.9154	Šidák's multiple comparisons test
Ex. Data Fig 7c	Closed Loop Stim Dark Phase Ensure Intake – not licking	PRLH-ChR2	5	0.9149	Šidák's multiple comparisons test
Ex. Data Fig 7c	Closed Loop Stim Dark Phase Ensure Intake – not licking	PRLH-ChR2 Control	5	0.5699	Šidák's multiple comparisons test
Ex. Data Fig 7c	Closed Loop Stim Dark Phase Ensure Bout size – not licking	PRLH-ChR2	5	0.4443	Šidák's multiple comparisons test
Ex. Data Fig 7c	Closed Loop Stim Dark Phase Ensure Bout size – not licking	PRLH-ChR2 Control	5	0.2431	Šidák's multiple comparisons test
Ex. Data Fig 7c	Closed Loop Stim Dark Phase Ensure Bout number – not licking	PRLH-ChR2	5	0.9702	Šidák's multiple comparisons test
Ex. Data	Closed Loop Stim	PRLH-	5	0.4143	Šidák's multiple comparisons test

Fig 7c	Dark Phase Ensure Bout number – not licking	ChR2 Control			
Fig 4c; Ex. Data Fig 7f	Closed Loop Inhibition Dark Phase Ensure Intake	PRLH-GtACR	6	0.1568	Šidák's multiple comparisons test
Fig 4c; Ex. Data Fig 7f	Closed Loop Inhibition Dark Phase Ensure Intake	PRLH-GtACR Control	5	0.9529	Šidák's multiple comparisons test
Fig 4c; Ex. Data Fig 7f	Closed Loop Inhibition Dark Phase Ensure Bout size	PRLH-GtACR	6	0.0010	Šidák's multiple comparisons test
Fig 4c; Ex. Data Fig 7f	Closed Loop Inhibition Dark Phase Ensure Bout size	PRLH-GtACR Control	5	0.9909	Šidák's multiple comparisons test
Fig 4c; Ex. Data Fig 7f	Closed Loop Inhibition Dark Phase Ensure Bout number	PRLH-GtACR	6	0.2158	Šidák's multiple comparisons test
Fig 4c; Ex. Data Fig 7f	Closed Loop Inhibition Dark Phase Ensure Bout number	PRLH-GtACR Control	5	0.5366	Šidák's multiple comparisons test
Fig 4g; Ex. Data Fig 7m	Preference ratio for bottle 1 – day 1 vs day 2	PRLH-ChR2	5	<0.0001	Dunnett's multiple comparisons test
Fig 4g; Ex. Data Fig 7m	Preference ratio for bottle 1 – day 1 vs day 3	PRLH-ChR2	5	0.4533	Dunnett's multiple comparisons test
Fig 4g; Ex. Data Fig 7m	Preference ratio for bottle 1 – day 1 vs day 2	PRLH-ChR2 Control	5	0.9136	Dunnett's multiple comparisons test
Fig 4g; Ex. Data Fig 7m	Preference ratio for bottle 1 – day 2 vs day 3	PRLH-ChR2 Control	5	0.8281	Dunnett's multiple comparisons test
Fig 4g;	Total Licks – day 1 vs day	PRLH-	5	0.7087	Dunnett's multiple comparisons test

Ex. Data Fig 7n	2	ChR2			
Fig 4g; Ex. Data Fig 7n	Total Licks – day 1 vs day 3	PRLH-ChR2	5	0.8851	Dunnett's multiple comparisons test
Fig 4g; Ex. Data Fig 7n	Total Licks – day 1 vs day 2	PRLH-ChR2 Control	5	0.4092	Dunnett's multiple comparisons test
Fig 4g; Ex. Data Fig 7n	Total Licks – day 1 vs day 3	PRLH-ChR2 Control	5	0.4224	Dunnett's multiple comparisons test
Fig 4h; Ex. Data Fig 7p	Preference ratio for bottle 1 – day 1 vs day 2	PRLH-GtACR	7	0.0092	Dunnett's multiple comparisons test
Fig 4h; Ex. Data Fig 7p	Preference ratio for bottle 1 – day 1 vs day 3	PRLH-GtACR	7	0.3847	Dunnett's multiple comparisons test
Fig 4h; Ex. Data Fig 7p	Preference ratio for bottle 1 – day 1 vs day 2	PRLH-GtACR Control	5	0.8266	Dunnett's multiple comparisons test
Fig 4h; Ex. Data Fig 7p	Preference ratio for bottle 1 – day 2 vs day 3	PRLH-GtACR Control	5	0.0942	Dunnett's multiple comparisons test
Fig 4h; Ex. Data Fig 7q	Total Licks – day 1 vs day 2	PRLH-GtACR	7	0.4187	Dunnett's multiple comparisons test
Fig 4h; Ex. Data Fig 7q	Total Licks – day 1 vs day 3	PRLH-GtACR	7	0.8714	Dunnett's multiple comparisons test
Fig 4h; Ex. Data Fig 7q	Total Licks – day 1 vs day 2	PRLH-GtACR Control	5	0.2897	Dunnett's multiple comparisons test
Fig 4h;	Total Licks – day 1 vs day	PRLH-	5	0.7846	Dunnett's multiple comparisons test

Ex. Data Fig 7q	3	GtACR Control			
Fig 6c; Ex. Data Fig 10c	Continuous Stim Dark Phase Ensure Intake	GCG-ChR2	7	0.0008	Šidák's multiple comparisons test
Fig 6c; Ex. Data Fig 10c	Continuous Stim Dark Phase Ensure Intake	GCG-ChR2 Control	7	0.9953	Šidák's multiple comparisons test
Ex. Data Fig 10c	Continuous Stim Dark Phase Ensure Bout size	GCG-ChR2	7	0.0023	Šidák's multiple comparisons test
Ex. Data Fig 10c	Continuous Stim Dark Phase Ensure Bout size	GCG-ChR2 Control	7	0.8232	Šidák's multiple comparisons test
Ex. Data Fig 10c	Continuous Stim Dark Phase Ensure Bout number	GCG-ChR2	7	0.0304	Šidák's multiple comparisons test
Ex. Data Fig 10c	Continuous Stim Dark Phase Ensure Bout number	GCG-ChR2 Control	7	0.7259	Šidák's multiple comparisons test
Fig 6c; Ex. Data Fig 10b	Continuous Stim FD Chow	GCG-ChR2	7	0.0004	Šidák's multiple comparisons test
Fig 6c; Ex. Data Fig 10b	Continuous Stim FD Chow	GCG-ChR2 Control	5	0.9775	Šidák's multiple comparisons test
Fig 6c; Ex. Data Fig 10d	Continuous Stim WD Water	GCG-ChR2	5	0.7512	Šidák's multiple comparisons test
Fig 6c; Ex. Data Fig 10d	Continuous Stim WD Water	GCG-ChR2 Control	5	0.4300	Šidák's multiple comparisons test
Fig 6f;	Pre-stim 1 hr	GCG-ChR2	7	0.0008	Šidák's multiple comparisons test

Ex. Data Fig 10f	Dark Phase Ensure Intake				
Fig 6f; Ex. Data Fig 10f	Pre-stim 1 hr Dark Phase Ensure Intake	GCG-ChR2 Control	5	0.8841	Šidák’s multiple comparisons test
Fig 6f; Ex. Data Fig 10f	Pre-stim 1 hr Dark Phase Ensure Bout size	GCG-ChR2	5	0.7828	Šidák’s multiple comparisons test
Fig 6f; Ex. Data Fig 10f	Pre-stim 1 hr Dark Phase Ensure Bout size	GCG-ChR2 Control	5	0.9796	Šidák’s multiple comparisons test
Fig 6f; Ex. Data Fig 10f	Pre-stim 1 hr Dark Phase Ensure Bout number	GCG-ChR2	7	0.0298	Šidák’s multiple comparisons test
Fig 6f; Ex. Data Fig 10f	Pre-stim 1 hr Dark Phase Ensure Bout number	GCG-ChR2 Control	5	0.9174	Šidák’s multiple comparisons test
Fig 6g	Chow Intake vs. Pre-stim duration	GCG-ChR2	6	0.0035	Simple linear regression
Fig 6h; Ex. Data Fig 10i	Pre-stim 1 hr Dark Phase Ensure Intake	PRLH-ChR2	7	0.7007	Šidák’s multiple comparisons test
Fig 6h; Ex. Data Fig 10i	Pre-stim 1 hr Dark Phase Ensure Intake	PRLH-ChR2 Control	5	0.9468	Šidák’s multiple comparisons test
Fig 6h; Ex. Data Fig 10i	Pre-stim 1 hr Dark Phase Ensure Bout size	PRLH-ChR2	7	0.8131	Šidák’s multiple comparisons test
Fig 6h; Ex. Data Fig 10i	Pre-stim 1 hr Dark Phase Ensure Bout size	PRLH-ChR2 Control	5	0.9558	Šidák’s multiple comparisons test
Fig 6h; Ex. Data	Pre-stim 1 hr Dark Phase Ensure	PRLH-ChR2	7	0.9976	Šidák’s multiple comparisons test

Fig 10i	Bout number				
Fig 6h; Ex. Data Fig 10i	Pre-stim 1 hr Dark Phase Ensure Bout number	PRLH-ChR2 Control	5	0.8067	Šidák's multiple comparisons test
Ex. Data Fig 7d	Number of laser pulses - closed loop stim licking vs not licking	PRLH-ChR2	5 and 6	0.0043	Mann Whitney test
Ex. Data Fig 7g	Closed Loop Inhibition Dark Phase Ensure Bout duration	PRLH-GtACR	6	0.0055	Holm-Šidák's multiple comparisons test
Ex. Data Fig 7g	Closed Loop Inhibition Dark Phase Ensure Bout duration	PRLH-GtACR Control	5	0.8496	Holm-Šidák's multiple comparisons test
Ex. Data Fig 7h	Closed Loop Inhibition Dark Phase Intralipid Intake	PRLH-GtACR	6	0.9959	Šidák's multiple comparisons test
Ex. Data Fig 7h	Closed Loop Inhibition Dark Phase Intralipid Intake	PRLH-GtACR Control	5	0.9758	Šidák's multiple comparisons test
Ex. Data Fig 7h	Closed Loop Inhibition Dark Phase Intralipid Bout size	PRLH-GtACR	6	0.0460	Šidák's multiple comparisons test
Ex. Data Fig 7h	Closed Loop Inhibition Dark Phase Intralipid Bout size	PRLH-GtACR Control	5	0.4356	Šidák's multiple comparisons test
Ex. Data Fig 7h	Closed Loop Inhibition Dark Phase Intralipid Bout number	PRLH-GtACR	6	0.3942	Šidák's multiple comparisons test
Ex. Data Fig 7h	Closed Loop Inhibition Dark Phase Intralipid Bout number	PRLH-GtACR Control	5	0.9994	Šidák's multiple comparisons test
Ex. Data Fig 7j	Mu1 - Ensure	PRLH-GtACR	6	0.6875	Wilcoxon Signed-Rank test

Ex. Data Fig 7j	Mu2 - Ensure	PRLH-GtACR	6	0.5625	Wilcoxon Signed-Rank test
Ex. Data Fig 7l	Mu1 - Intralipid	PRLH-GtACR	7	0.9375	Wilcoxon Signed-Rank test
Ex. Data Fig 7l	Mu2 - Intralipid	PRLH-GtACR	7	0.6875	Wilcoxon Signed-Rank test
Ex. Data Fig 7o	Bout size for bottle 1 vs bottle 2 - day 1	PRLH-ChR2	5	0.2613	Šidák's multiple comparisons test
Ex. Data Fig 7o	Bout size for bottle 1 vs bottle 2 - day 2	PRLH-ChR2	5	0.0001	Šidák's multiple comparisons test
Ex. Data Fig 7o	Bout size for bottle 1 vs bottle 2 - day 3	PRLH-ChR2	5	0.0011	Šidák's multiple comparisons test
Ex. Data Fig 7o	Bout size for bottle 1 vs bottle 2 - day 1	PRLH-ChR2 Control	5	0.9213	Šidák's multiple comparisons test
Ex. Data Fig 7o	Bout size for bottle 1 vs bottle 2 - day 2	PRLH-ChR2 Control	5	0.7198	Šidák's multiple comparisons test
Ex. Data Fig 7o	Bout size for bottle 1 vs bottle 2 - day 3	PRLH-ChR2 Control	5	0.9361	Šidák's multiple comparisons test
Ex. Data Fig 7o	Bout number for bottle 1 vs bottle 2 - day 1	PRLH-ChR2	5	0.0016	Šidák's multiple comparisons test
Ex. Data Fig 7o	Bout number for bottle 1 vs bottle 2 - day 2	PRLH-ChR2	5	0.0049	Šidák's multiple comparisons test
Ex. Data Fig 7o	Bout number for bottle 1 vs bottle 2 - day 3	PRLH-ChR2	5	0.0010	Šidák's multiple comparisons test
Ex. Data Fig 7o	Bout number for bottle 1 vs bottle 2 - day 1	PRLH-ChR2 Control	5	-.4951	Šidák's multiple comparisons test
Ex. Data	Bout number for bottle 1 vs	PRLH-	5	0.9780	Šidák's multiple comparisons test

Fig 7o	bottle 2 - day 2	ChR2 Control			
Ex. Data Fig 7o	Bout number for bottle 1 vs bottle 2 - day 3	PRLH-ChR2 Control	5	0.6056	Šidák's multiple comparisons test
Ex. Data Fig 7o	Licks for bottle 1 vs bottle 2 – day 1	PRLH-ChR2	5	0.0067	Šidák's multiple comparisons test
Ex. Data Fig 7o	Licks for bottle 1 vs bottle 2 – day 2	PRLH-ChR2	5	0.0015	Šidák's multiple comparisons test
Ex. Data Fig 7o	Licks for bottle 1 vs bottle 2 – day 3	PRLH-ChR2	5	0.0013	Šidák's multiple comparisons test
Ex. Data Fig 7o	Licks for bottle 1 vs bottle 2 – day 1	PRLH-ChR2 Control	5	0.1646	Šidák's multiple comparisons test
Ex. Data Fig 7o	Licks for bottle 1 vs bottle 2 – day 2	PRLH-ChR2 Control	5	0.7801	Šidák's multiple comparisons test
Ex. Data Fig 7o	Licks for bottle 1 vs bottle 2 – day 3	PRLH-ChR2 Control	5	0.5431	Šidák's multiple comparisons test
Ex. Data Fig 7r	Bout size for bottle 1 vs bottle 2 - day 1	PRLH-GtACR	7	0.3787	Šidák's multiple comparisons test
Ex. Data Fig 7r	Bout size for bottle 1 vs bottle 2 - day 2	PRLH-GtACR	7	0.0011	Šidák's multiple comparisons test
Ex. Data Fig 7r	Bout size for bottle 1 vs bottle 2 - day 3	PRLH-GtACR	7	<0.0001	Šidák's multiple comparisons test
Ex. Data Fig 7r	Bout size for bottle 1 vs bottle 2 - day 1	PRLH-GtACR Control	5	0.9350	Šidák's multiple comparisons test
Ex. Data Fig 7r	Bout size for bottle 1 vs bottle 2 - day 2	PRLH-GtACR Control	5	0.4474	Šidák's multiple comparisons test

Ex. Data Fig 7r	Bout size for bottle 1 vs bottle 2 - day 3	PRLH-GtACR Control	5	0.9918	Šidák's multiple comparisons test
Ex. Data Fig 7r	Bout number for bottle 1 vs bottle 2 - day 1	PRLH-GtACR	7	0.0724	Šidák's multiple comparisons test
Ex. Data Fig 7r	Bout number for bottle 1 vs bottle 2 - day 2	PRLH-GtACR	7	0.9856	Šidák's multiple comparisons test
Ex. Data Fig 7r	Bout number for bottle 1 vs bottle 2 - day 3	PRLH-GtACR	7	0.1658	Šidák's multiple comparisons test
Ex. Data Fig 7r	Bout number for bottle 1 vs bottle 2 - day 1	PRLH-GtACR Control	5	0.0581	Šidák's multiple comparisons test
Ex. Data Fig 7r	Bout number for bottle 1 vs bottle 2 - day 2	PRLH-GtACR Control	5	0.8784	Šidák's multiple comparisons test
Ex. Data Fig 7r	Bout number for bottle 1 vs bottle 2 - day 3	PRLH-GtACR Control	5	0.6297	Šidák's multiple comparisons test
Ex. Data Fig 7r	Licks for bottle 1 vs bottle 2 – day 1	PRLH-GtACR	7	0.1157	Šidák's multiple comparisons test
Ex. Data Fig 7r	Licks for bottle 1 vs bottle 2 – day 2	PRLH-GtACR	7	0.4394	Šidák's multiple comparisons test
Ex. Data Fig 7r	Licks for bottle 1 vs bottle 2 – day 3	PRLH-GtACR	7	0.0014	Šidák's multiple comparisons test
Ex. Data Fig 7r	Licks for bottle 1 vs bottle 2 – day 1	PRLH-GtACR Control	5	0.1447	Šidák's multiple comparisons test
Ex. Data Fig 7r	Licks for bottle 1 vs bottle 2 – day 2	PRLH-GtACR Control	5	0.3942	Šidák's multiple comparisons test
Ex. Data Fig 7r	Licks for bottle 1 vs bottle 2 – day 3	PRLH-GtACR	5	0.7291	Šidák's multiple comparisons test

		Control			
Ex. Data Fig 7s	Licks for bottle 1 vs 2 - sucralose	PRLH-GtACR	7	0.0310	Holm-Šidák's multiple comparisons test
Ex. Data Fig 7s	Licks for bottle 1 vs 2 - sucralose	PRLH-GtACR Control	4	0.6250	Holm-Šidák's multiple comparisons test
Ex. Data Fig 7s	Bout size for bottle 1 vs 2 - sucralose	PRLH-GtACR	7	0.0310	Holm-Šidák's multiple comparisons test
Ex. Data Fig 7s	Bout size for bottle 1 vs 2 - sucralose	PRLH-GtACR Control	4	0.6250	Holm-Šidák's multiple comparisons test
Ex. Data Fig 7s	Bout number for bottle 1 vs 2 - sucralose	PRLH-GtACR	7	0.1211	Holm-Šidák's multiple comparisons test
Ex. Data Fig 7s	Bout number for bottle 1 vs 2 - sucralose	PRLH-GtACR Control	4	0.3750	Holm-Šidák's multiple comparisons test
Ex. Data Fig 7t	Licks for bottle 1 vs 2 - water	PRLH-GtACR	7	0.9960	Holm-Šidák's multiple comparisons test
Ex. Data Fig 7t	Licks for bottle 1 vs 2 - water	PRLH-GtACR Control	5	>0.9999	Holm-Šidák's multiple comparisons test
Ex. Data Fig 7t	Bout size for bottle 1 vs 2 - water	PRLH-GtACR	7	0.8125	Holm-Šidák's multiple comparisons test
Ex. Data Fig 7t	Bout size for bottle 1 vs 2 - water	PRLH-GtACR Control	5	0.3398	Holm-Šidák's multiple comparisons test
Ex. Data Fig 7t	Bout number for bottle 1 vs 2 - water	PRLH-GtACR	7	0.9648	Holm-Šidák's multiple comparisons test
Ex. Data Fig 7t	Bout number for bottle 1 vs 2 - water	PRLH-GtACR Control	5	0.9648	Holm-Šidák's multiple comparisons test

Ex. Data Fig 10e	Closed Loop Stim Dark Phase Ensure Intake	GCG-ChR2	5	0.0042	Šidák's multiple comparisons test
Ex. Data Fig 10e	Closed Loop Stim Dark Phase Ensure Intake	GCG-ChR2 Control	7	0.6416	Šidák's multiple comparisons test
Ex. Data Fig 10e	Closed Loop Stim Dark Phase Ensure Bout size	GCG-ChR2	5	0.0013	Šidák's multiple comparisons test
Ex. Data Fig 10e	Closed Loop Stim Dark Phase Ensure Bout size	GCG-ChR2 Control	7	0.9815	Šidák's multiple comparisons test
Ex. Data Fig 10e	Closed Loop Stim Dark Phase Ensure Bout number	GCG-ChR2	5	0.0206	Šidák's multiple comparisons test
Ex. Data Fig 10e	Closed Loop Stim Dark Phase Ensure Bout number	GCG-ChR2 Control	7	0.5267	Šidák's multiple comparisons test
Ex. Data Fig 10g	Pre-stim 15 min FD Chow	GCG-ChR2	6	0.3567	Šidák's multiple comparisons test
Ex. Data Fig 10g	Pre-stim 15 min FD Chow	GCG-ChR2 Control	4	0.9422	Šidák's multiple comparisons test
Ex. Data Fig 10g	Pre-stim 30 min FD Chow	GCG-ChR2	6	0.0002	Šidák's multiple comparisons test
Ex. Data Fig 10g	Pre-stim 30 min FD Chow	GCG-ChR2 Control	4	0.9670	Šidák's multiple comparisons test
Ex. Data Fig 10g	Pre-stim 1 hr FD Chow	GCG-ChR2	7	<0.0001	Šidák's multiple comparisons test
Ex. Data Fig 10g	Pre-stim 1 hr FD Chow	GCG-ChR2 Control	5	0.7001	Šidák's multiple comparisons test
Ex. Data Fig 10h	Pre-stim 1 hr FD Chow	PRLH-ChR2	7	0.5483	Šidák's multiple comparisons test

Ex. Data Fig 10h	Pre-stim 1 hr FD Chow	PRLH-ChR2 Control	5	0.8474	Šidák’s multiple comparisons test

Statistics are show for each variable quantified during a given experiment in each figure. The number of mice (n) recorded in the experimental cohort are given alongside the p-value measured by the given statistical test. Cohorts of mice are separated by their genotype.
