# Supplementary material for: Sequential appetite suppression by oral and visceral feedback to the brainstem
Source: Nature. 2023 Nov 22;624(7990):130–7. doi: 10.1038/s41586-023-06758-2 (PMC10700140; doi:10.1038/s41586-023-06758-2)
Supplement: Supplementary file 2 — Reporting Summary [file 41586_2023_6758_MOESM2_ESM.pdf]

nature portfolio

Corresponding author(s): Zachary Knight

Last updated by author(s): Sep 22, 2023

Reporting Summary

Nature Portfolio wishes to improve the reproducibility of the work that we publish. This form provides structure for consistency and transparency in reporting. For further information on Nature Portfolio policies, see our Editorial Policies and the Editorial Policy Checklist.

Statistics

For all statistical analyses, confirm that the following items are present in the figure legend, table legend, main text, or Methods section.

n/a	Confirmed	
☐	☒	The exact sample size (*n*) for each experimental group/condition, given as a discrete number and unit of measurement
☐	☒	A statement on whether measurements were taken from distinct samples or whether the same sample was measured repeatedly
☐	☒	The statistical test(s) used AND whether they are one- or two-sided<br>*Only common tests should be described solely by name; describe more complex techniques in the Methods section.*
☐	☒	A description of all covariates tested
☐	☒	A description of any assumptions or corrections, such as tests of normality and adjustment for multiple comparisons
☐	☒	A full description of the statistical parameters including central tendency (e.g. means) or other basic estimates (e.g. regression coefficient) AND variation (e.g. standard deviation) or associated estimates of uncertainty (e.g. confidence intervals)
☐	☒	For null hypothesis testing, the test statistic (e.g. *F*, *t*, *r*) with confidence intervals, effect sizes, degrees of freedom and *P* value noted<br>*Give P values as exact values whenever suitable.*
☒	☐	For Bayesian analysis, information on the choice of priors and Markov chain Monte Carlo settings
☒	☐	For hierarchical and complex designs, identification of the appropriate level for tests and full reporting of outcomes
☒	☐	Estimates of effect sizes (e.g. Cohen's *d*, Pearson's *r*), indicating how they were calculated

Our web collection on statistics for biologists contains articles on many of the points above.

Software and code

Policy information about availability of computer code

Data collection	Synapse (TDT), Inscopix Data Acquisition Software (v151), Coulbourn Graphic State (v4.2)
Data analysis	GraphPad Prism (v9.0), Inscopix Data-Processing Software (v1.3.1), MathWorks Matlab (vR2017a), MOSAIC Data Processing Software (v.1.7), CNMF-E pipeline (http://www.github.com/zhoupc/cnmfe), BORIS (www.boris.unito.it)

For manuscripts utilizing custom algorithms or software that are central to the research but not yet described in published literature, software must be made available to editors and reviewers. We strongly encourage code deposition in a community repository (e.g. GitHub). See the Nature Portfolio guidelines for submitting code & software for further information.

Data

Policy information about availability of data

All manuscripts must include a data availability statement. This statement should provide the following information, where applicable:

- Accession codes, unique identifiers, or web links for publicly available datasets
- A description of any restrictions on data availability
- For clinical datasets or third party data, please ensure that the statement adheres to our policy

Data that support the findings of this study are available from the corresponding author upon reasonable request.

Human research participants

Policy information about studies involving human research participants and Sex and Gender in Research.

Reporting on sex and gender	*Use the terms sex (biological attribute) and gender (shaped by social and cultural circumstances) carefully in order to avoid confusing both terms. Indicate if findings apply to only one sex or gender; describe whether sex and gender were considered in study design whether sex and/or gender was determined based on self-reporting or assigned and methods used. Provide in the source data disaggregated sex and gender data where this information has been collected, and consent has been obtained for sharing of individual-level data; provide overall numbers in this Reporting Summary. Please state if this information has not been collected. Report sex- and gender-based analyses where performed, justify reasons for lack of sex- and gender-based analysis.*
Population characteristics	*Describe the covariate-relevant population characteristics of the human research participants (e.g. age, genotypic information, past and current diagnosis and treatment categories). If you filled out the behavioural & social sciences study design questions and have nothing to add here, write "See above."*
Recruitment	*Describe how participants were recruited. Outline any potential self-selection bias or other biases that may be present and how these are likely to impact results.*
Ethics oversight	*Identify the organization(s) that approved the study protocol.*

Note that full information on the approval of the study protocol must also be provided in the manuscript.

Field-specific reporting

Please select the one below that is the best fit for your research. If you are not sure, read the appropriate sections before making your selection.

☒ Life sciences ☐ Behavioural & social sciences ☐ Ecological, evolutionary & environmental sciences

For a reference copy of the document with all sections, see nature.com/documents/nr-reporting-summary-flat.pdf

Life sciences study design

All studies must disclose on these points even when the disclosure is negative.

Sample size	Power calculations were used to predetermine sample size when prediction of effect size was possible based on previous experiments. Otherwise, we based sample sizes on previous studies from our lab and others.
Data exclusions	No data were excluded except in the following experiments. Photometry recordings were removed if there was a technical issue with the lickometer (i.e. did not track licks). For Inscopix recordings, videos of cell activity were removed if excessive motion occurred (larger than the diameter of the average neuron in any direction).
Replication	We confirmed our main findings by repeating recordings and behavioral experiments in multiple animals, and repeating all recordings in the same animal whenever possible. All attempts at replication were successful.
Randomization	All functional experiments were counterbalanced. Recordings were not randomized, but within-animal controls were used whenever possible.
Blinding	Data analysis was performed automatically using the same Matlab scripts for each experimental group.

Reporting for specific materials, systems and methods

We require information from authors about some types of materials, experimental systems and methods used in many studies. Here, indicate whether each material, system or method listed is relevant to your study. If you are not sure if a list item applies to your research, read the appropriate section before selecting a response.

Materials & experimental systems

n/a	Involved in the study
☐	☒ Antibodies
☒	☐ Eukaryotic cell lines
☒	☐ Palaeontology and archaeology
☐	☒ Animals and other organisms
☒	☐ Clinical data
☒	☐ Dual use research of concern

Methods

n/a	Involved in the study
☒	☐ ChIP-seq
☒	☐ Flow cytometry
☒	☐ MRI-based neuroimaging

Antibodies

Antibodies used	chicken anti-GFP (Abcam, ab13970, 1:1000), rabbit anti-TH (Millipore, AB152), Alexa Fluor 488 goat anti-chicken (Life Technologies a11039, 1:500 or 1:1000).
Validation	Antibodies were validated for use in mouse brain sections in pilot experiments in our lab and by the manufacturers.

Animals and other research organisms

Policy information about studies involving animals; ARRIVE guidelines recommended for reporting animal research, and Sex and Gender in Research

Laboratory animals	We obtained wild-type (C57BL/6J, Jackson cat.no.000664), Gcg-iCre (B6;129S-Gcgtm1.1(icre)Gkg/J, Jackson cat.no.030663), Ai14 (B6.Cg-Gt(ROSA)26Sortm14(CAG-tdTomato)Hze/J, Jackson cat.no.007914), Ai213 (B6; 129S6-Igs7tm213(CAG-EGFP,CAG-mOrange2,CAG-mKate2)Hze/J, Jackson cat.no.034113), Trpm5-/- (B6.129P2-Trpm5tm1Dgen/J, Jackson cat.no.005848), Ai32 (B6.Cg-Gt(ROSA)26Sortm32(CAG-COP4*H134R/EYFP)Hze/J, Jackson cat.no.024109), and R26-LNL-GtACR1-Fred-Kv2.1 (B6;129S6-Gt(ROSA)26Sortm3Ksvo/J, Jackson cat.no.033089) mice from Jackson Labs. We obtained GCG-GFP mice as a gift from Hayashi Yoshitaka. Nano-L10 mice have been described previously (Ektrand et al. 2014). We obtained Dbh2A-FlpO (B6.129S7(FVB)-Dbhem2.1(flpo)Rray/Mmucd) mice from MMRRC (041575-UCD). Prlh-Cre knock-in mice were crossed to Ai14, GCG-GFP, and Nano-L10 mice to generate quadruple mutants. Prlh-Cre knock-in mice were crossed with Dbh2A-FlpO and Ai213 mice to generate triple mutants. Prlh-Cre knock-in mice or Gcg-iCre mice were crossed with Trpm5-/- mice to generate triple mutants (Prlh-Cre Trpm5-/- and Gcg-icre Trpm5-/- mice). Prlh-Cre knock-in mice and Gcg-icre mice were crossed with Ai32 mice to generate double mutants. Prlh-Cre knock-in mice were crossed with R26-LNL-GtACR1-Fred-Kv2.1 mice to generate double mutants.
Wild animals	No wild animals were used.
Reporting on sex	Adult mice (>6 weeks old) of both sexes were used for experiments. We obtained similar experimental results from both sexes and pooled the data.
Field-collected samples	No field-collected samples were used.
Ethics oversight	Experimental protocols were approved by the University of California, San Francisco IACUC following the NIH Guide for the Care and Use of Laboratory Animals.

Note that full information on the approval of the study protocol must also be provided in the manuscript.
